# MASTRO I: Meta-Analysis and Systematic Review of thrombectomy stent retriever outcomes: comparing functional, safety and recanalization outcomes between EmboTrap, Solitaire and Trevo in acute ischemic stroke

**DOI:** 10.57264/cer-2023-0001

**Published:** 2023-04-11

**Authors:** Osama O Zaidat, Shelly Ikeme, Sunil A Sheth, Shinichi Yoshimura, Xin-guang Yang, Waleed Brinjikji, David F Kallmes, Patrick Brouwer, John Pederson, Ranita Tarchand, Annie Steffenson, Kevin M Kallmes, Jillienne Touchette, Tommy Andersson

**Affiliations:** 1 Mercy St Vincent Medical Center, Toledo, OH 43608, USA; 2 Cardiovascular & Specialty Solutions Group, CERENOVUS, Irvine, CA 92618, USA; 3 Department of Neurology, UTHealth McGovern Medical School, Houston, TX 77030, USA; 4 Department of Neurosurgery, Hyogo College of Medicine, Hyogo, 663-8131, Japan; 5 Sun Yat-sen Memorial Hospital, Sun Yat-sen University, Guangzhou, Guangdong Province, 510123, China; 6 Department of Radiology, Mayo Clinic, Rochester, MN 55902, USA; 7 Superior Medical Experts, St. Paul, MN 55117, USA; 8 Nested Knowledge, Inc., St. Paul, MN 55117, USA; 9 Medical Imaging, AZ Groeninge, 8500, Kortrijk, Belgium; 10 Neuroradiology, Karolinska University Hospital & Clinical Neuroscience Karolinska Institute, 171 77, Stockholm, Sweden

**Keywords:** acute ischemic stroke, endovascular treatment, reperfusion, stent retriever, thrombectomy

## Abstract

**Aims:**

Stent-retriever (SR) thrombectomy has demonstrated superior outcomes in patients with acute ischemic stroke compared with medical management alone, but differences among SRs remain unexplored. We conducted a Systematic Review/Meta-Analysis to compare outcomes between three SRs: EmboTrap^®^, Solitaire™, and Trevo^®^.

**Methods:**

We conducted a PRISMA-compliant Systematic Review among English-language studies published after 2014 in PubMed/MEDLINE that reported SRs in ≥25 patients. Functional and safety outcomes included 90-day modified Rankin scale (mRS 0-2), mortality, symptomatic intracranial hemorrhage (sICH), and embolization to new territory (ENT). Recanalization outcomes included modified thrombolysis in cerebral infarction (mTICI) and first-pass recanalization (FPR). We used a random effects Meta-Analysis to compare outcomes; subgroup and outlier-influencer analysis were performed to explore heterogeneity.

**Results:**

Fifty-one articles comprising 9,804 patients were included. EmboTrap had statistically significantly higher rates of mRS 0-2 (57.4%) compared with Trevo (50.0%, p = 0.013) and Solitaire (45.3%, p < 0.001). Compared with Solitaire (20.4%), EmboTrap (11.2%, p < 0.001) and Trevo (14.5%, p = 0.018) had statistically significantly lower mortality. Compared with Solitaire (7.7%), EmboTrap (3.9%, p = 0.028) and Trevo (4.6%, p = 0.049) had statistically significantly lower rates of sICH. There were no significant differences in ENT rates across all three devices (6.0% for EmboTrap, 5.3% for Trevo, and 7.7% for Solitaire, p = 0.518). EmboTrap had numerically higher rates of recanalization; however, no statistically significant differences were found.

**Conclusion:**

The results of our Systematic Review/Meta-Analysis suggest that EmboTrap may be associated with significantly improved functional outcomes compared with Solitaire and Trevo. EmboTrap and Trevo may be associated with significantly lower rates of sICH and mortality compared with Solitaire. No significant differences in recanalization and ENT rates were found. These conclusions are tempered by limitations of the analysis including variations in thrombectomy techniques in the field, highlighting the need for multi-arm RCT studies comparing different SR devices to confirm our findings.

## Plain language summary

**What is this article about?:**

The standard treatment for acute stroke involves using minimally invasive stent-retrievers to remove clots. Many studies have reported outcomes for three commonly used stent-retrievers (EmboTrap^®^, Solitaire™, and Trevo^®^), but there are few studies comparing these devices directly. In the MASTRO I study, we systematically reviewed studies reporting stroke outcomes with these devices in order to combine and compare findings to determine whether any of them performed better in their ability to improve functional deficits, remove clots successfully or mitigate patient safety, such as hemorrhage in the brain, formation of distal clot fragments, and death.

**What were the results?:**

We found 51 studies reporting 9,804 patients treated for acute stroke with one of these three devices. When the 51 studies from the three devices were combined and compared, none of them did a significantly better job based on clot removal rates but EmboTrap had significantly better rates of achieving good neurological function than either Trevo or Solitaire. Also, with respect to safety, Solitaire had significantly higher rates of hemorrhage in the brain, and higher rates of death than either EmboTrap or Trevo. The rate of formation of distal clot fragments did not seem to differ among the three devices.

**What do the results mean?:**

The information from this comparative research review can help physicians with evidence-based decision making on which devices to use when treating acute stroke. It is worth noting that there was a potential for bias in the 51 studies combined in this review due to the high risk of comparing patient groups and treatment approaches that may not be similar, hence we highlight the fact that more randomized trials that control for any differences in study parameters are needed to confirm our results. There are two such randomized controlled trials that are ongoing, and we look forward to including these and any other new studies in future updates to this review in MASTRO II.

## Tweetable abstract

In the MASTRO I Meta-Analysis of stent-retrievers for AIS, the authors found higher rates of good neurological outcome for EmboTrap over Solitaire and Trevo, and improvements in sICH and mortality for EmboTrap and Trevo over Solitaire.

## Graphical and video abstract

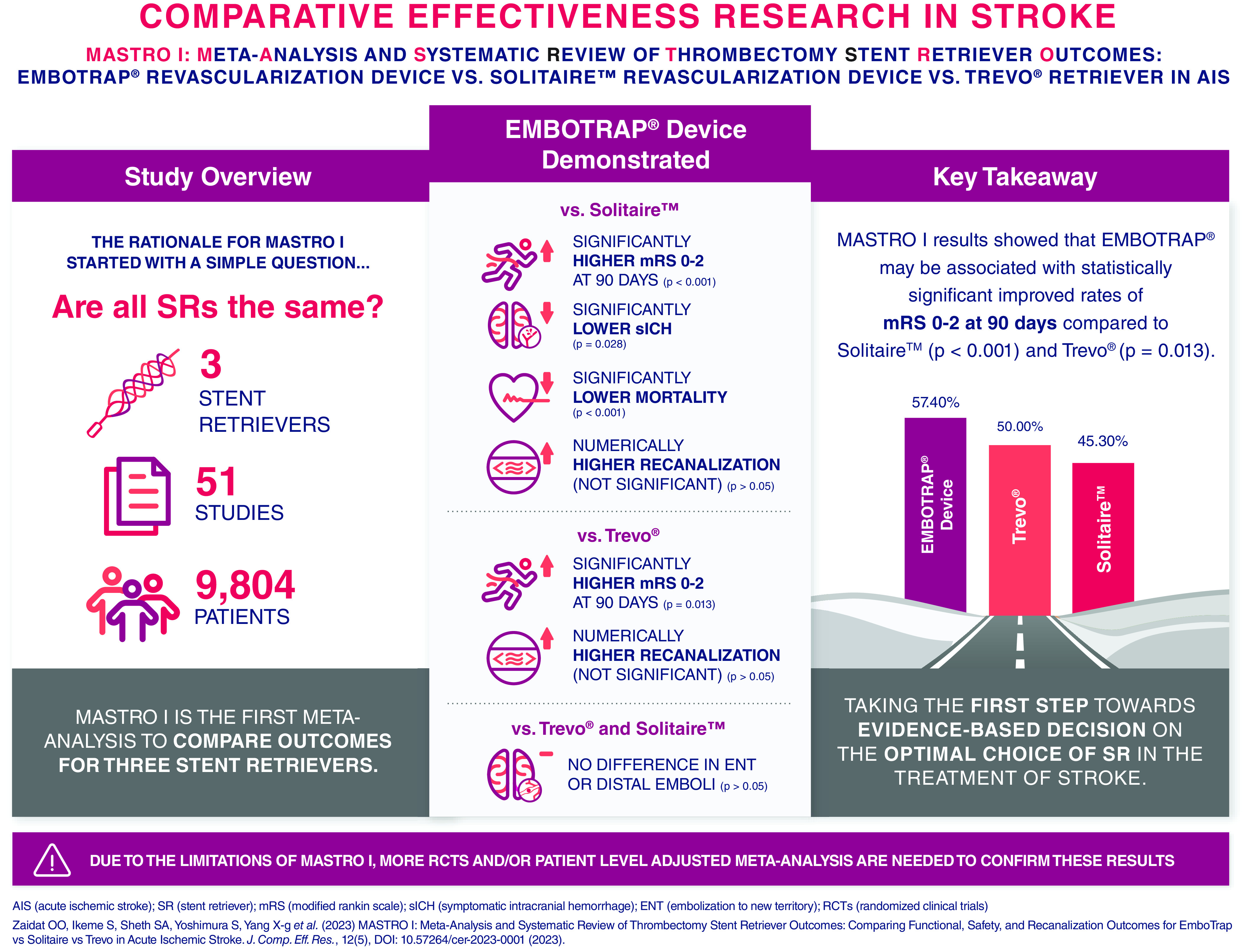

Mechanical thrombectomy (MT) using a stent-retriever (SR) has become the standard of care in patients with acute ischemic stroke (AIS) due to emergent large vessel occlusion (ELVO) and results in better outcomes than medical therapy alone [1–3]. While similar in their overall function, various thrombectomy devices have distinct features and mechanisms of action that may carry clinical consequences [4].

Solitaire™ (Solitaire, Medtronic, CA, USA) is a second-generation nitinol SR with a single-layer, overlapping parametric, closed-cell structure and peak-to-peak connections. It was frequently used in the major randomized controlled trials (RCTs) in 2015 that proved the efficacy of MT over best medical management (BMM), and has gone through four design iterations since the first-generation Solitaire AB; the current device generation is the Solitaire X.

Trevo^®^ (Trevo, CA, USA) is also a second-generation nitinol SR with a single-layer, closed-cell structure, with the major design differences from Solitaire being a non-overlapping design, the presence of braided tungsten/platinum wire, and a sodium hyaluronate coating. Like Solitaire, Trevo was used in the major RCTs in 2015, and was also found to improve clinical outcomes in an RCT against the first-generation SR, Merci device [5].

The EmboTrap^®^ Revascularization Device (EmboTrap, CERENOVUS, Johnson & Johnson Medical Devices, CA, USA) is a dual-layer segmented SR designed to entrap a broad range of clot compositions and stay apposed to the vessel wall during retrieval. The major design difference is the presence of a closed cell inner channel within the outer cage and a closed cell distal mesh at the tip [6]. EmboTrap has not been studied in any RCT, but was reported in an initial prospective, multi-center study to have high reperfusion rates (mTICI ≥2b and mTICI 2c/3 of 80.2 and 64.8%, respectively) and a high modified first pass recanalization (mFPR) mTICI ≥2b rate of 51.5% [7].

Currently, studies directly comparing SRs are scarce. Most studies of MT have compared strategies and techniques, such as direct aspiration first-pass techniques versus SRs, rather than assessing the relative advantages of different devices [8]. While a pre-clinical Systematic Review of four studies comparing EmboTrap and Solitaire predicted that EmboTrap could lead to better recanalization outcomes [9], these pre-clinical studies are exploratory, and the only way to determine whether these mechanical differences lead to differing outcomes is through clinical comparisons.

To address this research gap, and in the absence of controlled trials comparing SRs, we conducted a living Systematic Review and Meta-Analysis among studies of patients with AIS treated with the three most studied SRs (EmboTrap, Solitaire and Trevo) to compare functional, safety, and recanalization outcomes, with the hypothesis that outcomes will not differ across devices.

## Methods

The authors declare that all supporting data are available within the article and its Supplement.

### Literature search & study selection

This study adhered to Preferred Reporting Items for Systematic Reviews and Meta-Analyses (PRISMA) reporting of search and screening [10] and to the Meta-analysis of Observational Studies in Epidemiology (MOOSE) Guidelines. The initial search was completed on the PubMed database using the AutoLit platform in Nested Knowledge and identified relevant articles published between 1 January 2015 and 11 March 2022 [11] (see search strings in Supplementary Table 1). Identified studies were evaluated for inclusion criteria, which included English-language studies reporting adult patients with AIS due to ELVO who were treated with EmboTrap^®^, Solitaire™, or Trevo^®^ SRs and reported functional, safety and recanalization outcomes.

Studies were excluded if they: did not use a device of interest, did not report any outcomes of interest, only reported combination treatment (SR used with an aspiration device), did not separate patient outcomes by technique or device used, had <25 patients, did not relate to AIS, were the wrong study type (*in vivo/in vitro* study, symposium/conference, case report, qualitative review, letter of correspondence, *in silico* study/mathematical model, guideline article, technical note, editorial/opinion, cost-effectiveness study, survey study, economic study without angiographic or clinical outcomes, Meta-Analysis/Systematic Review, secondary analysis, protocol or interim analysis), did not report patients treated with MT, reported a biased subset of the stroke population (such as reporting only posterior-location strokes) or full text was unavailable. The reference lists of identified articles were also screened for potentially relevant papers.

The justification for these exclusion reasons was that they narrow to only clinical studies reporting substantial results for one or more of the three candidate devices, while limiting population bias. For instance, while we did not require studies to be anterior-only, we excluded studies that reported only posterior strokes, as this subset of strokes have demonstrably worse outcomes. Where possible, we excluded studies reporting the use of SR in combination with aspiration catheters to narrow in on the impact of the SRs outside of the context of aspiration-with-SR. One author screened the studies for inclusion and two authors independently reviewed inclusion. Detailed results of the study search, screening, and data extraction process are hosted on the Nested Knowledge website (www.nested-knowledge.com) [11]. Additionally, the living Systematic Review database hosted on the Nested Knowledge platform will be continually updated to inform future publication of additional findings. Updated results are anticipated in 18 to 24 months.

### Data extraction

Functional, safety and recanalization outcomes reported by underlying articles with sufficient reporting frequency were extracted from all included articles. Data extraction from selected studies was completed by one author and independently confirmed for accuracy by two additional authors. Patient baseline characteristics included Alberta Stroke Program Early CT Scores (ASPECTS) [12], pre-operative mRS, National Institutes of Health Stroke Scale (NIHSS) score, clot location and age, as well as procedural practices: use of a balloon guide catheter (BGC) and use of intravenous tissue-type plasminogen activator (IV-tPA). Efficacy outcomes included recanalization rates, using the thrombolysis in cerebral infarction (TICI) scale and its modifications (modified TICI [mTICI] and expanded TICI [eTICI]). Outcomes were FPR mTICI ≥2c (complete or near-complete FPR), mFPR mTICI ≥2b (successful FPR), final TICI (complete recanalization; inclusive of TICI complete recanalization equivalents TICI 3, mTICI 3, and eTICI 3) and final mTICI ≥2b (successful recanalization). Functional and safety and outcomes included 90-day modified Rankin Scale (mRS), 90-day mortality, symptomatic intracranial hemorrhage (sICH) and embolization to new territory (ENT).

If data were available in secondary analyses, these data were added to that collected from the primary publication of a study or trial. If any data element was reported by both primary and secondary studies, the data from the primary study was preferred. Secondary analyses were otherwise excluded to prevent overlap in patient populations.

### Statistical analysis

A separate hierarchical random effects model was fit for each outcome measure, with random effects models run with respect to each study and all intervention(s) nested within studies. Event counts and corresponding sample sizes were recorded for dichotomous outcomes. As appropriate, the Haldane-Anscambe correction was conditionally applied to correct for zero-cell counts in dichotomous data [13,14]. Due to their skewed nature, data were logarithmically transformed prior to analysis to produce an approximately normal distribution and to decrease the influence of high leverage data points. Transformed proportions and corresponding standard errors were pooled using the generic inverse variance method and using the DerSimonian-Laird procedure [15]. Outcome rates from dichotomous data were reported as percentages (i.e., predicted events per 100 observations), and pooled percentages are reported as random effects estimates. Ninety-five percent confidence intervals (CI) of the pooled results of each random effects Meta-Analysis were computed [16]. For comparisons of ordinal scale mRS data, pooled median mRS scores and corresponding 95% CIs were derived via random effects models using methods described by McGrath *et al.* [17]. To aid in interpretation, the logarithmically transformed pooled results were back-transformed to pooled percentages or median values. Higgin's I^2^ statistics were used to estimate the percentage of variability in effect estimates due to heterogeneity rather than sampling error [18]. Additionally, 95% CIs around individual study estimates were computed using the Clopper–Pearson exact binomial interval. No network meta-analytical methods were used since most included studies were single-arm and non-comparative.

Statistical significance of between-group comparisons was determined by p-values <0.05. Statistics were performed in RStudio (Version 1.3.959) using the ‘meta’ [19] and ‘metamedian’ packages [20].

Within each complete-case analysis, exploratory subgroup analysis was completed on core lab-adjudicated studies and on prospective-only studies, using the same statistical methods outlined above.

### Risks of selection bias

In any review of observational studies, a risk of bias due to the selection of patients can arise (both in which patients' outcomes were published and in selection among published articles). As the common risk of bias tools such as Cochrane's RoB2 are generally only applicable to multi-armed studies, other approaches to mitigate risk of bias were performed in this review. First, specific reasons for potential population-related and procedure-related bias were pre-determined and applied as part of the screening process; for all studies excluded based on potential bias, specific reasons were recorded in the Results. Furthermore, a statistical outlier-influencer analysis was performed to detect impact of heterogenous findings on an outcome-by-outcome basis.

### Heterogeneity & outlier-influencer analysis

To explore patterns of effect sizes and heterogeneity in outcome data, an outlier-influencer analysis was undertaken. Briefly, graphic displays of study heterogeneity (GOSH) plots were generated after performing all 2^k^-1 possible study combinations, or 1 million randomly selected iterations for analyses that exceeded 1 million possible models. In a perfectly homogenous hypothetical study population, all iterations displayed in the GOSH plot should converge on a singular distribution with one peak. We used a three-model cross validation technique using unsupervised machine learning algorithms (including k-means clustering, density based spatial clustering of applications with noise [DBSCAN], and Gaussian mixture model [GMM] clustering) to detect clusters in GOSH plot data and determine which studies contributed the most to between-study heterogeneity.

The three-clustering technique calculates cluster imbalance of a specific study using the difference between the expected proportion of subsets containing a specific study, given that the cluster composition is purely random, and the actual proportion of subsets containing a specific study within a given cluster. An outlier/influential case was defined as a study with a Cook's distance three-times above the mean across the generated clusters. Outlier and influence analyses were only performed if there were at least five studies per treatment group that reported the variable of interest. Studies that were detected as outliers on all three algorithms were excluded in sub-analyses to explore patterns in effect sizes and heterogeneity impacted by high-leverage outliers (i.e., studies with extreme values that differs significantly from the overall effect) and influential cases (i.e., studies that have a large impact on the pooled effect or heterogeneity, regardless of how high or low the individual study estimate was).

## Results

**Figure 1.**
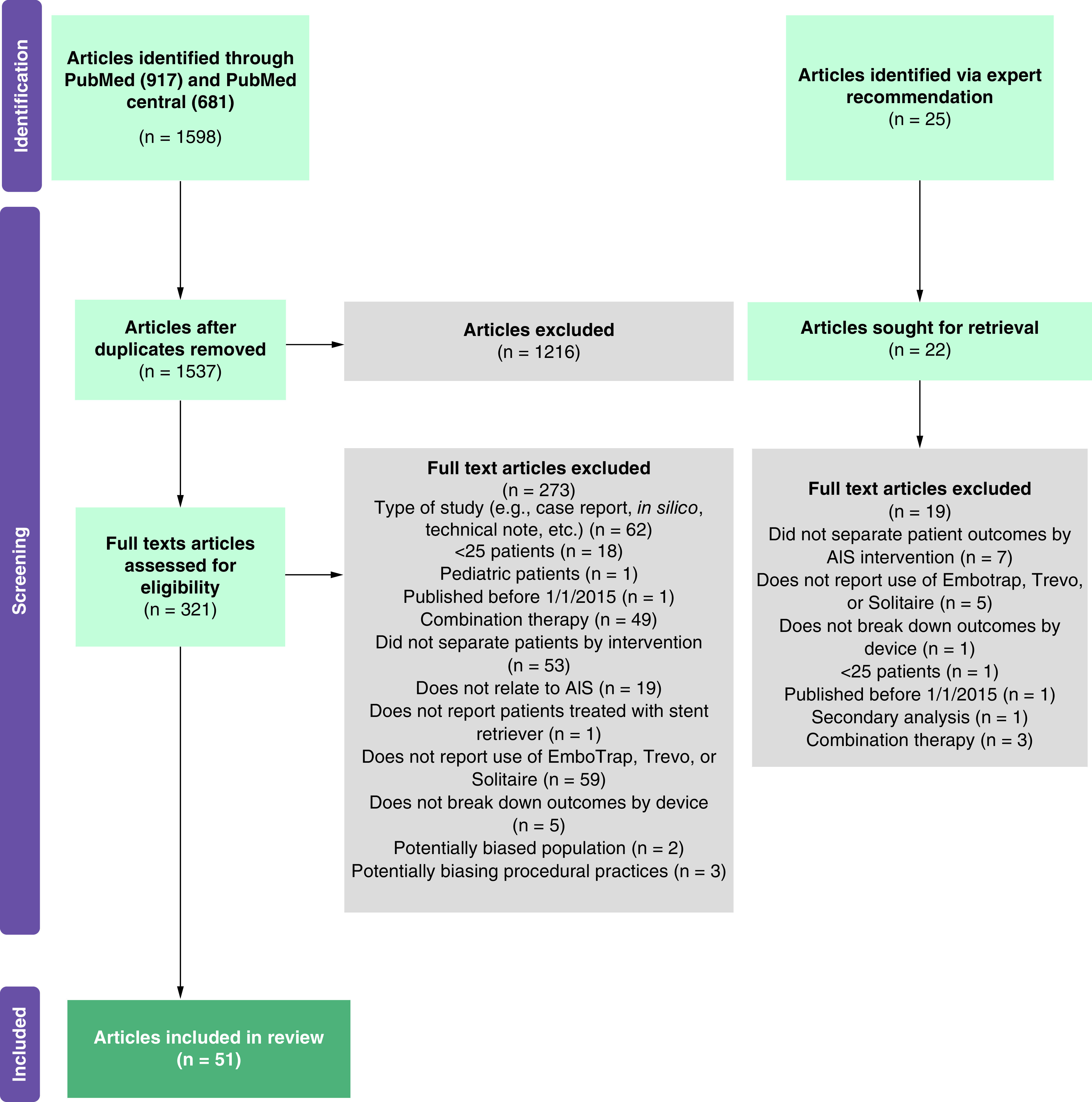
PRISMA study flow diagram. Articles identified by expert recommendation were identified by the authors by searching the bibliographies of previous Systematic Reviews on related topics. Articles with potentially biasing populations (n = 22) were excluded at both the abstract (n = 20) and full text (n = 2) stages of review.

The search identified 1598 records; after removing duplicates, 1537 articles remained. Based on title/abstract review, 1216 articles were excluded, leaving 321 articles for full-text review. An additional 25 articles were identified via expert recommendation, of which three were excluded in title/abstract review and 22 were sent forward for full-text review. A PRISMA diagram of study attrition with reasons for exclusion is shown in Figure 1; for the 22 studies that were excluded due to potentially biased populations (abstract review n = 20 and full text review n = 2), the reasons were: vertebrobasilar occlusions only (9), Intracranial Atherosclerotic Disease (ICAD) patients only (4), reperfused patients only (3), Posterior Inferior Cerebellar Artery (PICA) occlusions only (1), craniocervical occlusions only (1), very late window (>24 h since onset; 1), cancer patients only (1), tandem occlusions only (1), stroke as a complication of carotid endarterectomy (1) (to see all excluded records, Supplementary Table 2).

Ultimately, 51 studies were included in the Meta-Analysis (Supplementary Table 3). Study types included five randomized control trials [21–25], seven registry studies [26–32], and nine prospective cohort studies [7,33–40]; the remaining 30 studies were retrospective [34,41–70].

### Background characteristics & procedural details

The total sample comprised 9804 patients. Studies reporting treatment with each device included seven studies of EmboTrap comprising 687 patients [7,30,33,35,43,53,63], thirty-four studies of Solitaire comprising 5,690 patients, and fourteen studies of Trevo comprising 3427 patients. Eleven studies reported core-lab adjudicated angiographic results, including two EmboTrap studies [7,33], seven Solitaire studies [21–23,25,32,39,45], and two Trevo studies [24,26].

The mean age of patients in the EmboTrap, Solitaire and Trevo groups were 69.2, 66.4 and 68.7 years, respectively. The baseline mean NIHSS scores were 16.1 for EmboTrap, 16.8 for Solitaire, and 16.1 for Trevo. The mean ASPECTS scores were 9.1 for EmboTrap, 9.5 for Solitaire, but not reported in underlying studies for Trevo. The median ASPECTS scores were 10 for EmboTrap, 8 for Solitaire and 8 for Trevo. For EmboTrap, 648/687 (94.3%) of occlusions were in the anterior circulation and 39/687 (5.7%) were in the posterior circulation, while for Trevo, 3101/3427 (90.5%) of occlusions were anterior and 253/3427 (7.4%) were posterior, and for Solitaire, 5428/5611 (96.7%) were anterior and 144/5611 (2.6%) were posterior.

BGC use patterns were reported in 5/7 (71.4%) EmboTrap study arms, in 8/14 (57.1%) study arms for Trevo, and in 16/34 (47.1%) study arms for Solitaire. Among studies that reported BGC use, rates were 60.8% (271/446; 95% CI: 56.1–65.3%) for EmboTrap, 54.6% (1663/3048; 95% CI: 52.8–56.3%) for Trevo, and 49.6% (906/1826; 95% CI: 47.3–51.9%) for Solitaire. The EmboTrap group had a statistically significant higher rate of BGC use compared with both the Trevo group (p = 0.014) and the Solitaire group (p < 0.001) and the Trevo group had a significantly higher rate of BGC use compared with the Solitaire group (p < 0.001). Of study arms that reported IV-tPA use (7/7 [100%] of EmboTrap study arms, 13/14 [92.9%] for Trevo, and 30/34 [88.2%] for Solitaire), rates were 52.7% (362/687; 95% CI: 48.9–56.5%) for EmboTrap, 49.1% (1646/3354; 95% CI: 47.4–50.8%) for Trevo, and 38.4% (1434/3730; 95% CI: 36.9–40.0%) for Solitaire. The EmboTrap group had a statistically significant higher rate of IV-tPA use compared with the Solitaire group (p < 0.001) and trended toward a higher rate compared with the Trevo group, but the difference was not statistically significant (p = 0.086). Additionally, the Trevo group had a statistically significant higher rate of IV-tPA use compared with the Solitaire group (p < 0.001). See Table 1 for pooled summary statistics of baseline characteristics and Supplementary Table 3 for a complete list of study and patient baseline characteristics at the study level.

**Table 1.** Pooled summary statistics of background characteristics across EmboTrap, Solitaire and Trevo.

Studies reporting (Per device: [ET, TR, SO])	Patient characteristics	EmboTrap	Trevo	Solitaire
34 (5, 7, 24)	Age, mean (SD), n^†^	69.2 (13.5), 567	68.7 (14.3), 2971	66.4 (13.1), 2802
12 (1, 5, 6)	Age, median, n^‡^	72.0, 80	74.6, 379	71.4, 395
47 (7, 13, 30)	Use of IVT, n/n (%) [95% CI]^§^ {p-value}	362/687 (52.7%) [48.9–56.5] {vs Trevo: 0.086 vs Solitaire: <0.001}	1646/3354 (49.1%) [47.4–50.8] {vs Solitaire: <0.001}	1434/3730 (38.4%) [36.9–40.0]
28 (5, 8, 16)	BGC Utilization – n/n (%) [95% CI]^§^ {p-value}	271/446 (60.8%) [56.1–65.3] {vs Trevo: 0.014 vs Solitaire: <0.001}	1663/3048 (54.6%) [52.8–56.3] {vs Solitaire: <0.001}	906/1826 (49.6%) [47.3–51.9]
37 (5, 8, 24)	sICH Definitions	ECASS III – 4 studies SITS-MOST – 1 Not defined – 2 sICH not reported - 0	ECASS II – 7 studies ECASS III – 4 SITS-MOST – 3 Heidelberg Bleeding Classification – 4 SWIFT – 4 PROACT II – 2 Not defined – 6 sICH not reported - 5	ECASS II – 1 study ECASS III – 6 SITS-MOST – 1 *Not defined – 2* *sICH not reported - 4*
24 (4, 6, 19)	Preoperative NIHSS, mean (SD), n^†^	16.1 (5.4), 366	16.1 (6.8), 2797	16.8 (5.9), 1995
25 (4, 8, 14)	Preoperative NIHSS, median^‡^	15.4, 548	16.5, 541	17.0, 1624
6 (2, 0, 4)	ASPECTS score, mean (SD), n^†^	9.1 (1.5), 256	N/A	9.5 (1.8), 482
16 (2, 5, 9)	ASPECTS score, median^‡^	10.0, 307	8.0, 2280	8.0, 665
46 (7, 13, 29)	Stroke location, n/n (%)	–	–	–
	Anterior	648/687 (94.3%)	3101/3427 (90.5%)	5428/5611 (96.7%)
	Posterior	39/687 (5.7%)	253/3427 (7.4%)	144/5611 (2.6%)
	Tandem	14/486 (3.7%)	11/3372 (0.3%)	67/3095 (2.1%)
	ACA (any location)	1/687 (0.1%)	39/3342 (1.2%)	7/3012 (0.2%)
	A1	0/486 (0%)	4/2733 (0.1%)	1/2880 (<0.1%)
	A2	0/486 (0%)	19/2733 (0.7%)	0/2880 (0%)
	A3	0/486 (0%)	11/2733 (0.4%)	0/2880 (0%)
	BA	5/687 (0.7%)	64/3296 (1.9%)	126/3398 (3.7%)
	ICA	134/687 (19.5%)	560/3372 (16.6%)	993/3666 (27.1%)
	MCA (any location)	520/687 (75.7%)	2482/3342 (74.3%)	2287/3536 (64.7%)
	M1	238/406 (58.6%)	1868/3372 (55.4%)	1163/2071 (56.2%)
	M2	78/406 (19.2%)	595/3372 (17.6%)	188/1717 (10.9%)
	M3	2/486 (0.4%)	42/3342 (1.3%)	2/2279 (<0.1%)
	PCA	34/687 (4.9%)	15/3342 (0.4%)	3/3012 (<0.1%)
	VA	0/687 (0%)	174/3342 (5.2%)	13/3012 (0.4%)

† Calculated using a simple weighted mean from studies reporting mean, standard deviation and sample size.

‡ Calculated using a weighted median of medians approach in the Nested Knowledge software.

§ Dichotomous outcomes reported as n/N (random effects estimate) (95% CI). CIs calculated using an exact binomial interval.

ACA: Anterior cerebral artery; ASPECTS: Alberta Stroke Program Early Computed Tomography Score; BA: Basilar artery; BGC: Balloon guide catheter; ET: EmboTrap^®^; ICA: Internal carotid artery; IVT: Intravenous thrombolysis; NIHSS: National Institutes of Health Stroke Scale; MCA: Middle cerebral artery; PCA: Posterior cerebral artery; TR: Trevo; SO: Solitaire; SD: Standard deviation; VA: Vertebral artery.

### Functional & safety outcomes

#### Modified rankin scale (mRS) score at 90 Days

Among the 44 studies with sufficient data, pooled rates of mRS 0-2 for EmboTrap (57.4%, 95% CI: 50.2–64.4) were significantly higher compared with both Trevo (50.0%, 95% CI: 46.5–53.5; p = 0.013) and Solitaire (45.3%, 95% CI: 43.0–47.7; p < 0.001) (Table 2). No significant differences were found between Solitaire vs Trevo.

**Table 2.** Comparison of functional, safety, and recanalization outcomes between EmboTrap, Trevo, and Solitaire, with pooled random effects estimates.

Outcomes	Studies reporting outcome (n)	Study arms reporting outcome (n)	EmboTrap^†^	Trevo^†^	Solitaire^†^	p-value (EmboTrap vs Trevo)	p-value (EmboTrap vs Solitaire)	p-value (Trevo vs Solitaire)	I^2^ (EmboTrap + Trevo) (95% CI) p-value	I^2^ (EmboTrap + Solitaire) (95% CI) p-value	I^2^ (Trevo + Solitaire) (95% CI) p-value	Overall I^2^ (95% CI) p-value
mRS 0-2 at 90 days	44	47	374/633 (57.4%) [50.2–64.4]	1667/3160 (50.0%) [46.5–53.5]	2258/5118 (45.3%) [43.0–47.7]	0.013	<0.001	0.056	59.3% (32.5–75.4) <0.001	71.0% (59.3–79.4) <0.001	70.4% (59.2–78.5) <0.001	74.1% (65.6–80.5) <0.001
mRS at 90 days	22	23	1.63 (0.94–2.32) [475]	2.45 (1.93–2.98) [2598]	2.73 (2.30–3.17) [2893]	0.118	0.018	0.441	93.1% (81.8–98.2) <0.001	92.2% (85.2–96.7) <0.001	91.6% (83.7–96.5) <0.001	93.4% (87.9–96.9) <0.001
0	.	.	100 (21.1%)	497 (19.1%)	360 (12.5%)	.	.	.	.	.	.	.
1	.	.	92 (19.4%)	508 (19.6%)	479 (16.6%)	.	.	.	.	.	.	.
2	.	.	98 (20.6%)	387 (14.9%)	448 (15.5%)	.	.	.	.	.	.	.
3	.	.	48 (10.1%)	332 (12.8%)	401 (13.9%)	.	.	.	.	.	.	.
4	.	.	63 (13.3%)	346 (13.3%)	354 (12.2%)	.	.	.	.	.	.	.
5	.	.	19 (4.0%)	128 (4.9%)	177 (6.1%)	.	.	.	.	.	.	.
6	.	.	55 (11.6%)	400 (15.4%)	673 (23.3%)	.	.	.	.	.	.	.
Mortality at 90 days	43	46	71/658 (11.2%) [8.9–13.9]	485/3270 (14.5%) [11.4–18.4]	933/4122 (20.4%) [17.9–23.1]	0.127	<0.001	0.018	68.1% (49.2–79.9) <0.001	65.9% (48.5–77.4) <0.001	79.8% (73.0–84.9) <0.001	79.8% (73.6–84.6) <0.001
ENT or distal emboli	27	28	35/607 (6.0%) [2.8–12.1]	96/2872 (5.3%) [3.1–8.9]	214/2162 (7.7%) [5.2–11.3]	0.816	0.536	0.276	80.5% (67.5–88.3) <0.001	84.0% (76.5–89.1) <0.001	89.9% (86.2–92.6) <0.001	88.5% (84.6–91.4) <0.001
sICH	44	46	22/687 (3.9%) [2.3–6.6]	102/3084 (4.6%) [2.6–8.1]	420/5257 (7.7%) [6.2–9.4]	0.514	0.028	0.049	72.4% (55.1–83.0) <0.001	69.7% (57.5–78.4) <0.001	80.7% (74.2–85.5) <0.001	78.7% (72.1–83.8) <0.001
FPR mTICI ≥2c	5	6	91/227 (40.1%) [N/A]^‡^	406/1493 (23.1%) [13.9–36.0]	123/381 (32.4%) [27.9–37.3]	N/A	N/A	0.220	89.3% (71.0–96.1) <0.001	46.3% (0.0–82.1) 0.134	55.9% (0.0–83.7) 0.060	77.5% (50.0–89.9) <0.001
mFPR mTICI ≥2b	11	12	156/307 (50.8%) [45.2–56.4]	131/304 (42.1%) [27.4–58.3]	409/997 (41.0%) [35.8–46.3]	0.328	0.197	0.796	80.5% (57.9–91.0) <0.001	67.8% (32.2–84.7) 0.003	74.3% (52.0–86.3) <0.001	74.9% (55.7–85.7) <0.001
TICI 3	28	29	163/307 (53.1%) [47.5–58.6]	699/2566 (47.0%) [29.3–65.6]	1449/3247 (46.5%) [40.0–53.1]	0.637	0.617	0.997	97.8% (97.0–98.4) <0.001	88.2% (88.3–91.6) <0.001	95.8% (94.8–96.7) <0.001	95.7% (94.6–96.5) <0.001
mTICI ≥2b	31	32	479/548 (86.6%) [80.1–91.3]	2109/2569 (82.8%) [80.3–85.0]	3300/4018 (81.7%) [78.1–84.8]	0.526	0.111	0.248	57.1% (18.3–77.4) 0.007	79.5% (70.1–85.9) <0.001	74.1% (62.5–82.1) <0.001	74.6% (64.1–82.0) <0.001

† Dichotomous data for individual subgroups are expressed as n/n (%) [95% CI]; % calculated represents pooled random effects estimate, not fixed percentage).

‡ Since only one study has the reported outcome and the between-study variance component cannot be estimated, 95% CIs for pooled outcomes and pairwise comparisons between different interventions are not reported.

Ordinal data for individual subgroups are expressed as pooled median (95% CI) [n], along with raw frequency counts and percentages for each ordinal score. All pooled estimates for dichotomous data are derived from random-effects models using the DerSimonian-Laird procedure for estimation of between-study variance [15]; 95% CIs of the pooled results were computed using the Jackson method [16]. Pooled medians and corresponding 95% CIs were derived via random effects models using methods described by McGrath *et al.* [17] P-values for each pairwise comparison are provided using separate meta-regression analyses, considering the intervention as a categorical moderator. P-values for the overall heterogeneity (i.e., statistical heterogeneity) among the included studies are obtained from Q-tests of heterogeneity. The estimated percentage of variability in effect size estimates that is due to heterogeneity rather than sampling error is given by I^2^ statistics and their corresponding 95% CIs [18]. I^2^ values are given for each subgroup comparison and for the overall study population.

CI: Confidence interval; ENT: Embolization to new territory; FPR: First pass recanalization; mRS: Modified Rankin Scale; mTICI: Modified thrombolysis in cerebral infarction; sICH: Symptomatic intracranial hemorrhage; TICI: Thrombolysis in cerebral infarction.

After excluding four outlier studies [7,26,38,47], 41 studies had sufficient data for comparisons of mRS 0-2 at 90 days. Patients treated with EmboTrap had a significantly higher pooled rate of mRS 0-2 at 90 days (54.9%, 95% CI: 48.7–61.1) compared with Solitaire (46.2%, 95% CI: 43.8–48.6; p = 0.008, Supplementary Figures 1–3). No significant differences were found when compared with Trevo; pooled rates for Solitaire and Trevo (50.0%, 95% CI: 46.5–53.5) did not differ significantly, but the directionality and magnitude of effect estimates were similar to the complete-case analysis (Table 3). Overall, compared with the complete-case analysis, there was a 12% relative reduction in statistical heterogeneity (I^2^ = 74.1%, 95% CI: 65.6–80.5 vs I^2^ = 65.2%, 95% CI: 52.2–74.7).

**Table 3.** Comparison of functional and safety outcomes between EmboTrap, Trevo, and Solitaire after removing outlier studies, with pooled random effects estimates.

Clinical and safety outcomes	Studies reporting outcomes (n)	Study arms reporting outcome (n)	EmboTrap^†^	Trevo^†^	Solitaire^†^	p-value (EmboTrap vs Trevo)	p-value (EmboTrap vs Solitaire)	p-value (Trevo vs Solitaire)	I^2^ (EmboTrap + Trevo) (95% CI) p-value	I^2^ (EmboTrap + Solitaire) (95% CI) p-value	I^2^ (Trevo + Solitaire) (95% CI) p-value	Overall I^2^ (95% CI) p-value
mRS 0-2 at 90 days	41	44	228/416 (54.9%) [48.7–61.1]	1667/3160 (50.0%) [46.5–53.5]	2018/4493 (46.2%) [43.8–48.6]	0.157	0.008	0.085	39.0% (0.0–65.2) 0.047	52.7% (29.2–68.4) <0.001	66.3% (52.6–76.0) <0.001	65.2% (52.2–74.7) <0.001
mRS at 90 days	20	21	1.63 (0.94–2.32) [475]	2.45 (1.93–2.98) [2598]	2.73 (2.26–3.20) [2893]	0.118	0.018	0.441	85.7% (57.2–96.9) <0.001	89.5% (79.2–96.0) <0.001	91.8% (83.9–96.7) <0.001	93.4% (87.9–96.9) <0.001
0	.	.	100 (21.1%)	497 (19.1%)	360 (12.5%)	.	.	.	.	.	.	.
1	.	.	92 (19.4%)	508 (19.6%)	479 (16.6%)	.	.	.	.	.	.	.
2	.	.	98 (20.6%)	387 (14.9%)	448 (15.5%)	.	.	.	.	.	.	.
3	.	.	48 (10.1%)	332 (12.8%)	401 (13.9%)	.	.	.	.	.	.	.
4	.	.	63 (13.3%)	346 (13.3%)	354 (12.2%)	.	.	.	.	.	.	.
5	.	.	19 (4.0%)	128 (4.9%)	177 (6.1%)	.	.	.	.	.	.	.
6	.	.	55 (11.6%)	400 (15.4%)	673 (23.3%)	.	.	.	.	.	.	.
Mortality at 90 days	38	41	51/436 (12.2%) [9.4–15.7]	481/3172 (15.7%) [12.4–19.6]	679/3254 (18.5%) [16.0–21.3]	0.159	0.023	0.260	62.6% (37.8–77.5) <0.001	73.0% (62.3–80.7) <0.001	71.6% (61.2–79.2) <0.001	71.6% (61.2–79.2) <0.001
ENT or distal emboli	24	25	24/537 (4.5%) [2.4–8.4]	50/875 (6.4%) [4.3–9.5]	157/1961 (7.3%) [5.5–9.6]	0.366	0.167	0.624	40.9% (0.0–70.8) 0.077	62.0% (36.7–77.2) <0.001	58.6% (33.0–74.5) <0.001	59.1% (36.5–73.7) <0.001
sICH	42	44	22/687 (3.9%) [2.3–6.6]	68/1076 (6.7%) [5.3–8.4]	319/4629 (7.6%) [6.4–8.9]	0.169	0.015	0.265	9.2% (0.0–46.1) 0.348	45.0% (17.9–63.1) 0.003	33.2% (0.0–55.4) 0.028	36.6% (8.4–56.1) 0.009

† Dichotomous data for individual subgroups are expressed as n/n (%) [95% CI]; % calculated represents pooled random effects estimate, not fixed percentage).

Note: Outlier and influencer analyses were only performed if there were at least 5 studies per treatment group that reported the variable of interest. As such, outlier and influencer analyses were only performed for comparisons of mRS 0-2 at 90 days, mortality at 90 days, sICH, and ENT.

Ordinal data for individual subgroups are expressed as pooled median (95% CI) [n], along with raw frequency counts and percentages for each ordinal score. All pooled estimates for dichotomous data are derived from random-effects models using the DerSimonian-Laird procedure for estimation of between-study variance [15]; 95% CIs of the pooled results were computed using the Jackson method [16]. Pooled medians and corresponding 95% CIs were derived via random effects models using methods described by McGrath *et al.* [17] P-values for each pairwise comparison are provided using separate meta-regression analyses, considering the intervention as a categorical moderator. P-values for the overall heterogeneity (i.e., statistical inconsistency) among the included studies are obtained from Q-tests of heterogeneity. The estimated percentage of variability in effect size estimates that is due to heterogeneity rather than sampling error is given by I^2^ statistics and their corresponding 95% CIs [18]. I^2^ values are given for each subgroup comparison and for the overall study population.

CI: Confidence interval; ENT: Embolization to new territory; FPR: First pass recanalization; mRS: Modified Rankin Scale; mTICI: Modified thrombolysis in cerebral infarction; sICH: Symptomatic intracranial hemorrhage; TICI: Thrombolysis in cerebral infarction.

Ordinal mRS scores are reported and compared in Supplementary Results Section 1 & Supplementary Table 4.

#### Mortality at 90 days

Among the 43 studies with sufficient data, the pooled rate of mortality at 90 days for Solitaire (20.4%, 95% CI: 17.9–23.1) was significantly higher compared with EmboTrap (11.2%, 95% CI: 8.9–13.9; p < 0.001) and Trevo (14.5%, 95% CI: 11.4–18.4; p = 0.018). There was no statistically significant difference between EmboTrap and Trevo with respect to mortality (p = 0.127) (Table 2).

After excluding five outlier studies, 38 studies had sufficient data for comparisons of 90-day mortality rates. EmboTrap (12.2%, 95% CI: 9.4–15.7%) had a significantly lower pooled 90-day mortality rate compared with Solitaire (18.5%, 95% CI: 16.0–21.3%; p = 0.023, Supplementary Figures 4–6); pooled rates for Trevo (15.7%, 95% CI: 12.4–19.6%) did not differ significantly from the other devices (Table 3). Overall, the directionality and magnitude of effect estimates were similar to the complete-case analysis, but there was a 10% relative reduction in statistical heterogeneity (I^2^ = 79.8%; 95% CI: 73.6–84.6 vs I^2^ = 71.6%; 95% CI: 61.2–79.2).

#### Embolization to new territory (ENT) or distal emboli

Among the 27 studies with sufficient data, pooled rates of emboli (reflecting total reporting of ENT and distal emboli) did not differ significantly according to the omnibus test of subgroup differences (p = 0.518) and were 6.0% (95% CI: 2.8–12.1) for EmboTrap, 5.3% (95% CI: 3.1–8.9) for Trevo, and 7.7% (95% CI: 5.2–11.3) for Solitaire (Table 2).

After excluding 3 outlier findings [26,28,30], 24 studies had sufficient data for comparisons of ENT rates. Pooled rates of ENT and distal emboli did not differ significantly between EmboTrap (4.5%, 95% CI: 2.4–8.4), Trevo (6.4%, 95% CI: 4.3–9.5), and Solitaire (7.3%, 95% CI: 5.5–9.6; Supplementary Figures 7 & 8). Overall, results did not substantially differ compared with the complete-case analysis, but directionality of outcome comparisons changed, with results favoring Trevo before removing outliers and results favoring the EmboTrap group after outlier removal. There was a 33% relative reduction in statistical heterogeneity compared with the complete-case analysis (I^2^ = 88.5%; 95% CI: 84.6–91.4 vs I^2^ = 59.1%, 95% CI: 36.5–73.7). Outcome comparisons after outlier removal for ENT/distal emboli and other functional and safety outcomes are presented in Table 3.

#### Symptomatic intracranial hemorrhage (sICH)

Among the 44 studies with sufficient data, pooled rates of sICH for Solitaire (7.7%, 95% CI: 6.2–9.4) were significantly higher compared with both EmboTrap (3.9%, 95% CI: 2.3–6.6; p = 0.028) and Trevo (4.6%, 95% CI: 2.6–8.1; p = 0.049) (Table 2). No significant differences were found in sICH rates between EmboTrap and Trevo (p = 0.514).

After excluding two outlier studies [26,66], 42 studies had sufficient data for comparisons of sICH rates. EmboTrap (3.9%, 95% CI: 2.3–6.6) maintained significantly lower pooled rate of sICH compared with Solitaire (7.6%, 95% CI: 6.4–8.9%; p = 0.015; Supplementary Figures 9 & 10); pooled rates of sICH for Trevo were 6.7% (95% CI: 5.3–8.4) and did not differ significantly from the other devices (Table 3). Overall, outcome comparisons did not differ between the complete-case analysis and the outlier analysis, and the observed statistical heterogeneity had a relative reduction of 54% (I^2^ = 78.7%; 95% CI: 72.1–83.8 vs I^2^ = 36.6%; 95% CI: 8.4–56.1).

Studies differed in their definitions of sICH; where defined, studies used one of the following definitions: ECASS II, ECASS III, PROACT II, SWIFT, SITS-MOST, or the Heidelberg Bleeding Classification. For a full breakdown of sICH definitions by device, see Table 1, and for a breakdown by study – with the exact wording of each sICH definition – Supplementary Table 3.

### Recanalization outcomes

#### Complete or near-complete recanalization on first pass (FPR mTICI ≥2c)

Among the 5 studies with sufficient data, pooled rates of FPR mTICI ≥2c were 40.1% (95% CI not available) for EmboTrap, 23.1% (95% CI: 13.9–36.0) for Trevo, and 32.4% (95% CI: 27.9–37.3) for Solitaire (Table 2). A formal statistical comparison between EmboTrap and other devices was not performed as only one EmboTrap study reported this outcome, hence between-study variance could not be estimated. Pooled rates of FPR mTICI ≥2c did not differ significantly between Solitaire and Trevo (p = 0.220; Supplementary Figure 11). Outlier-influencer analyses were not performed for FPR mTICI ≥2c and subsequent recanalization outcomes due to limited number of studies reporting data.

#### Successful recanalization on first pass (mFPR mTICI ≥2b)

Among the 11 studies with sufficient data, pooled rates of mFPR mTICI ≥2b were 50.8% (95% CI: 45.2–56.4) for EmboTrap, 42.1% (95% CI: 27.4–58.3%) for Trevo, and 41.0% (95% CI: 35.8–46.3) for Solitaire; these rates did not differ significantly (p = 0.430; Supplementary Figure 12 & Table 2).

#### Final complete recanalization (TICI 3)

Among the 28 studies with sufficient data, pooled rates of TICI 3 did not differ significantly (p = 0.879, Supplementary Figure 13) between EmboTrap (53.1%, 95% CI: 47.5–58.6), Trevo (47.0%, 95% CI: 29.3–65.6), and Solitaire (46.5%, 95% CI: 40.0–53.1) when used as first line therapy (Table 2). Reported rates of TICI 3 varied substantially between studies, with an estimated 95.7% (95% CI: 94.6–96.5) of the variability attributable to heterogeneity rather than sampling error.

#### Final successful recanalization (mTICI ≥2b)

Among the 31 studies with sufficient data, pooled rates of mTICI ≥2b did not differ according to the omnibus test of subgroup differences (p = 0.202; Supplementary Figure 14) and were 86.6% (95% CI: 80.1–91.3%) for EmboTrap, 82.8% (95% CI: 80.3–85.0%) for Trevo, and 81.7% (95% CI: 78.1–84.8%) for Solitaire (Table 2).

### Subanalyses on core lab adjudicated & prospective-only studies

In order to identify and address sources of heterogeneity, the authors completed pre-specified subanalyses on studies where recanalization outcomes were core lab adjudicated and on studies with prospective designs. Overall, compared with the complete-case analysis there was no significant difference. The full results of these subanalyses can be found in Supplementary Results 2 & 3, in Supplementary Tables 5 & 6 & Supplementary Figures 15–25.

## Discussion

In our analysis, EmboTrap demonstrated significantly higher rates of mRS 0-2 at 90-days compared with both Solitaire and Trevo and lower rates of 90-day mortality and sICH compared with Solitaire. When Trevo and Solitaire were compared, Trevo demonstrated significantly lower rates of mortality and sICH, though improvements in sICH were not robust following outlier analysis. No significant differences in ENT rates were found. Final recanalization outcomes (successful recanalization mTICI ≥2b and complete recanalization TICI 3) for EmboTrap trended numerically higher compared with Solitaire and Trevo, but these differences in recanalization rates were not statistically significant in the complete-case analysis, nor in the subgroup analyses of core lab-adjudicated and prospective-only studies. Similarly, mFPR and FPR for EmboTrap trended numerically higher compared with Solitaire and Trevo; however due to a dearth of studies reporting FPR mTICI ≥2c, a formal statistical analysis, including outlier and influence analysis could not be performed. The dearth of FPR data in stroke studies was expected, as FPR was first defined and reported in 2018 by Zaidat *et al.* [71]

 Though there were inherent limitations to our Systematic Review and Meta-Analysis (discussed below), our analysis identified several clinical outcomes for which a significant difference was found between the three SR devices. These findings will need to be confirmed through controlled studies that directly compare approaches; we have noted the recent registration of three RCT studies: the SOLTRAP study comparing Solitaire to EmboTrap (NCT05518240), the ENVI RCT study comparing Envi™-SR to Solitaire/Trevo (NCT05107206) study, and the PROST study comparing Solitaire to the pRESET Thrombectomy device (NCT03994822) as active ongoing studies with direct comparison of SR devices. The pRESET and ENVI RCT studies are FDA registrational studies establishing a new scientific benchmark for stroke device trials. Furthermore, we adopted living Systematic Review methods for this review, which may help address this gap as we intend to update this Review and Meta-Analysis in MASTRO II as more evidence emerges.

While these findings are informative, they must be seen in the context of a potentially heterogenous patient population. Mean age, NIHSS, and ASPECTS, where reported, were relatively consistent across underlying different SR studies. In terms of stroke location, posterior strokes have been shown to have consistently worse clinical outcomes, hence the exclusion of studies that reported only posterior strokes. In this review, however, the device with the lowest proportion of posterior strokes, Solitaire, did not have improved clinical outcomes over the other devices; this does not indicate that stroke location had no impact on outcomes, but rather a greater proportion of anterior strokes did not lead to improved clinical performance for Solitaire.

Furthermore, differences in the procedural workflow beyond SR device choice may have contributed to differences in outcomes. For instance, the fact that pooled rates of BGC use and IV-tPA use were significantly higher for EmboTrap and Trevo compared with Solitaire may have contributed to more favorable outcomes [72,73]. As none of the studies included in our Meta-Analysis reported outcomes based on BGC/IV-tPA use, it was impractical to methodologically determine if BGC or IV-tPA use influenced outcomes. While variation in procedural practices were narrowed as much as possible – for instance, excluding techniques combining SRs with aspiration—other differences in procedural practice, such as differences in the sizes and lengths of each device, and differences in operator experience or change in practices over the period of this Meta-Analysis, were impossible to control for in our Meta-Analysis. Lastly, while most outcomes had standardized definitions, sICH definitions varied across studies, with ECASS III used by the majority of both EmboTrap (four studies) and Trevo (seven studies), while Solitaire had a wider range of definitions used, potentially impacting the reported rates of sICH across studies.

The limitations in controlling for background and procedural variables in our review, and the moderate to high heterogeneity in many of the comparisons in this Meta-Analysis, made mitigating heterogeneity a priority in this review. To that regard, in addition to excluding studies based on potentially biasing population characteristics or procedural practices, an outlier-influencer analysis was performed to detect potential sources of heterogeneity that may disproportionately sway results in favor of any given treatment group. Heterogeneity was systematically lower in outlier-adjusted analysis, and both safety and functional outcomes had changes in significant findings after adjustment for outliers. This limitation could be addressed in the future with further multi-arm studies of the devices in question. Our Meta-Analysis found only four comparative studies (all comparing Solitaire to Trevo), so there is a pressing need to further study these devices in a comparative, controlled context such as an RCT in order to determine the true impact of device-based differences in stroke outcomes. A controlled study would also harmonize the differential reporting of certain outcomes, such as emboli (which were reported variously as ENT, distal emboli, or combined in underlying studies) and sICH (which can be defined using a range of different criteria, such as the ECASS II, ECASS III, and Heidelberg definitions, which have shown different sICH findings based on scale used).

Another benefit of further RCT-level comparisons of the devices in question would be the use of core lab adjudication of angiographic results. In this Meta-Analysis, no differences in performance were found when site-adjudicated data were analyzed alongside core, but when the core-adjudicated subset was analyzed, substantially lower rates of mFPR mTICI ≥2b were found for Solitaire and Trevo, and the rate of TICI 3 for Trevo fell by more than half. This reflects potential site-adjudication bias, aligning with previous findings that TICI ratings have been previously found to vary by observer, sometimes leading to inflated reported rates of success. Our core lab subanalysis supported the continued use of core lab adjudication where possible, for the final reporting of any angiographic findings that may be subject to inaccuracy or bias in site-level assessment.

EmboTrap was designed to improve clot engagement in thrombectomy across a range of clots with varying composition [74]. While EmboTrap outperformed other SRs in the current study, to date, no head-to-head RCTs comparing SRs for use in MT for AIS have been published. However, a recent pre-clinical Systematic Review to investigate the impact of device design on MT success found that EmboTrap performed significantly better than Solitaire (p < 0.01), particularly for friable (p < 0.05) and standard (p < 0.05) clot types [9]. In a previous Meta-Analysis of SR alone versus SR plus aspiration (combination technique), Mohammaden *et al.* found that treatment with SR alone or combination technique resulted in similar rates of FPR, mFPR, and final successful reperfusion, as well as comparable functional and safety outcomes in general. However, when the data were stratified by SR device and aspiration catheter size, the authors found that, unlike Solitaire and Trevo, when used in combination with an aspiration catheter with an inner diameter ≥0.068”, EmboTrap was associated with significantly better rates of FPR, mFPR, and recanalization prior to rescue, and attributed this finding to EmboTrap's design/geometry differences [75]. These results may have suggested that design differences in EmboTrap may translate into meaningful differences in recanalization, and when viewed in the context of our analysis and device comparison, these design differences may have also impacted important safety and clinical outcomes.

These promising results must be balanced against the limited number of prospective and large-population studies of EmboTrap. Among the seven studies of EmboTrap included in our review, two were prospective cohort studies, the Analysis of Revascularization in Ischemic Stroke with EmboTrap I and II (ARISE I and ARISE II) [7,35]. These initial studies reported high reperfusion rates (mTICI ≥2b) of 85.0% [35] and 92.5% [7], respectively. As clinical trials have generally been more restrictive, the ARISE clinical trial may have included a less diverse patient and provider population or provided a higher level of clinical and follow-up care. However, real-world studies using EmboTrap have demonstrated similar results. Four European studies have been conducted since 2016; one reported a reperfusion rate of 95.0% (TICI ≥2b) [53], and three documented rates of 84.6–90.0% (mTICI ≥2b) [33,43,63]. A US multi-center registry of patients treated with EmboTrap II, a device iteration that incorporates a double proximal marker for more precise stent placement and an increase from 3 to 5 outer cages, found a similarly high rate of successful reperfusion of 95.7% (TICI ≥2b) [30]. Similarly, the rates of sICH for patients treated with EmboTrap found in the clinical trials are confirmed by real-world EmboTrap studies. ARISE II found that 5.3% of trial patients experienced a sICH, while the rate in real-world studies ranged from 0.0% to 6.3% [30,33,43,63]. These studies were recently published in a single-arm review of EmboTrap treatment that corroborates our EmboTrap-specific findings: Bai *et al.* [76] meta-analyzed the aforementioned studies as well as two publications that report EmboTrap in combination with aspiration catheters [77,78]. Bai *et al.* corroborated the findings of MASTRO I, in that rates of successful mTICI 2b–3 (90%) and mFPR mTICI 2b/3 (43%) were slightly higher than our findings, while FPR mTICI 2c/3 (36%) and mRS 0–2 (53%) were slightly lower. Safety outcomes were slightly better in our analysis than in Bai *et al.*, who found sICH of 5% and mortality of 14%, as well as similar heterogeneity to our findings before the outlier analysis. MASTRO I thus expanded on Bai *et al.* by providing an outlier analysis to limit heterogeneity while also contextualizing EmboTrap findings relative to other leading stent retrievers.

While we noted statistically significantly improved differences in the rate of sICH and mortality among patients treated with Trevo compared with Solitaire, these differences were no longer statistically significant after outlier studies were removed. In the case of sICH, Binning *et al.* [26] found an sICH rate of 1.7% in a sample of 2,008 patients treated with Trevo, which represented an outlier. Hence, this finding substantially shifted results toward a lower sICH pooled rate for Trevo. After removal of the Binning *et al.* results, Trevo lost its significant difference in sICH compared with Solitaire, suggesting that the Trevo results are not robust to the outlier. Among Solitaire studies, Yang *et al.* was an outlier with an sICH rate of 16.1%, perhaps due to the relatively high rate of difficult strokes included in the study sample [65]. However, removal of this study did not have a large impact on Solitaire findings, suggesting that the pooled estimate for Solitaire is relatively robust to the outlier. Overall, after removal of these outliers, reduced heterogeneity (I^2^) [2] was observed and the estimated percentage of variability in effect estimates for sICH was much lower and revealed a more consistent pattern. In the same vein, the ARISE II clinical trial of EmboTrap was also found to be an influential outlier with respect to functional outcomes. The removal of ARISE II, and other outlier studies, reduced I^2^ by 12% and attenuated the difference in 90-day mRS 0-2 found between EmboTrap and other SRs. Although the differences in 90-day mRS 0-2 remained statistically significantly favorable for EmboTrap over Solitaire, the difference compared with Trevo was no longer statistically significant.

While it is not always possible to determine what aspect of a study or patient population impacted outcomes, some potential factors include procedural or operator differences (i.e., use of BGCs), more difficult patient populations (i.e., higher proportion of ICA vs MCA strokes which may be more difficult to treat) [79,80], differences in study design factors (such as age and NIHSS cut-offs), timing of events (patients treated more quickly), timing of study (more modern technologies/standards of care may improve outcomes), and differences in outcome definitions (such as use of ECASS III (European Cooperative Acute Stroke Study) versus the New Heidelberg Bleeding Classification to define sICH) [81]. The impact of outlier-influencer analysis underscores the variability in the studies. While we attempted to identify and reduce this heterogeneity in our analysis, this type of heterogeneity is common in meta-analyses of stroke studies [82], which are mostly observational, and further underscores the need for RCTs in this area.

## Limitations

A limitation of this Meta-Analysis was that single-arm study results are aggregated and then compared, which can lead to differences in outcomes based on population and procedural differences. As such, large differences in outcomes may be attributable to differences within-study rather than differences in the effectiveness of the devices. Furthermore, network Meta-Analysis could not be performed due to the single-arm nature of most included studies. Although subgroup and outlier-influencer analyses were performed, which significantly reduced heterogeneity for some outcome measures assessed, moderate to high heterogeneity between studies was observed. This moderate to high heterogeneity was likely due to differences in study design, hence causal inferences about the comparative effectiveness of the devices should be made with caution. The generalizability of our study findings may be limited by population characteristic bias, differences in procedural practice, or even under-reporting of key variables by the underlying studies.

To address these limitations, the authors limited bias by adhering to MOOSE guidelines, restricting studies with potentially biased populations, and performing rigorous outlier-influencer and subgroup analysis to address heterogeneity. In furtherance, by performing a living Systematic Review the authors aim to strengthen the analysis by updating MASTRO II with new evidence as they become available.

## Conclusion

EmboTrap may be associated with significantly improved functional outcomes compared with Solitaire and Trevo. EmboTrap and Trevo may be associated with significantly lower rates of mortality and sICH compared with Solitaire. No significant differences in recanalization and ENT rates were found. Due to some important limitations, more RCTs and/or patient-level adjusted meta-analyses are needed to confirm these results.

**Summary points**

- We report the MASTRO I living Systematic Review and Meta-Analysis, comparing functional, safety, and recanalization outcomes between three commonly used stent-retrievers (EmboTrap, Solitaire, and Trevo).
- We conducted a MOOSE- and PRISMA-compliant Systematic Review in the Nested Knowledge living review platform. We included 51 English-language studies with 9,804 patients, published from January, 2015 to March, 2022.
- Functional and safety outcomes: EmboTrap had statistically significantly higher rates of mRS 0-2 (57.4%) compared with Trevo (50.0%, p = 0.013) and Solitaire (45.3%, p < 0.001). Compared with Solitaire (20.4%), EmboTrap (11.2%, p < 0.001) and Trevo (14.5%, p = 0.018) had statistically significantly lower mortality. Compared with Solitaire (7.7%), EmboTrap (3.9%, p = 0.028) and Trevo (4.6%, p = 0.049) had statistically significantly lower rates of sICH. There were no significant differences in ENT rates (6.0% for EmboTrap, 5.3% for Trevo, and 7.7% for Solitaire, p = 0.518).
- Recanalization outcomes: EmboTrap had numerically higher rates but no significant differences in first-pass and final recanalization rates across devices for the following: mFPR mTICI ≥2b (50.8% for EmboTrap, 42.1% for Trevo, and 41.0% for Solitaire, p = 0.430), rates of FPR mTICI ≥2c (40.1% for EmboTrap, 23.1% for Trevo, and 32.4% Solitaire, p = 0.220), Final TICI 3 (53.1% for EmboTrap, 47.0% for Trevo, and 46.5% for Solitaire, p = 0.879), and rates of Final mTICI ≥2b (86.6% for EmboTrap, 82.8% for Trevo, and 81.7% for Solitaire p = 0.202).
- In summary, EmboTrap may have been associated with significantly improved mRS 0-2 rates compared with Solitaire and Trevo. EmboTrap and Trevo may have been associated with significantly lower rates of mortality and sICH compared with Solitaire. No significant differences in ENT and recanalization rates were found.
- These conclusions were tempered by limitations of the analysis including potential variations in patient population and thrombectomy techniques, the single-arm nature of most included studies, and the moderate to high heterogeneity for certain outcomes.
- Further randomized studies are needed to confirm the findings of MASTRO I; two such randomized trials are currently underway: the SOLTRAP study (NCT05518240) and the PROST study (NCT03994822).
