# Supplementary material for: MASTRO I: Meta-Analysis and Systematic Review of thrombectomy stent retriever outcomes: comparing functional, safety and recanalization outcomes between EmboTrap, Solitaire and Trevo in acute ischemic stroke
Source: J Comp Eff Res. 2023 Apr 11;12(5):e230001. doi: 10.57264/cer-2023-0001 (PMC10402757; doi:10.57264/cer-2023-0001)

**SUPPLEMENTAL MATERIALS**

**MASTRO I: Meta-Analysis and Systematic Review of Thrombectomy Stent Retriever Outcomes: Comparing Functional, Safety and Recanalization Outcomes between EmboTrap, Solitaire, and Trevo in Acute Ischemic Stroke**

Osama O. Zaidat MS MD^1^, Shelly Ikeme MBA MPH PharmD PhD^2^, Sunil A. Sheth MD^3^, Shinichi Yoshimura MD PhD^4^, Xin-guang Yang PhD^5^, Waleed Brinjikiji MD^6^, David F. Kallmes MD^6^, Patrick Brouwer MS MD^2^, John Pederson BS^7^, Ranita Tarchand MS^8^, Annie Steffenson PhD^7^, Kevin M. Kallmes MA JD^7,8^, Jillienne Touchette PhD^7^, Tommy Andersson MD PhD^9,10^

^1^Mercy St Vincent Medical Center, Toledo, OH

^2^ Cardiovascular & Specialty Solutions Group, CERENOVUS, Irvine, CA

^3^ Department of Neurology, UTHealth McGovern Medical School, Houston, TX

^4^ Department of Neurosurgery, Hyogo College of Medicine, Hyogo, Japan

^5^ Sun Yat-sen Memorial Hospital, Sun Yat-sen University, Guangzhou, China

^6^ Department of Radiology, Mayo Clinic, Rochester, MN

^7^ Superior Medical Experts, St. Paul, MN

^8^ Nested Knowledge, Inc., St. Paul, MN

^9^ Medical Imaging, AZ Groeninge, Kortrijk, Belgium

^10^ Neuroradiology, Karolinska University Hospital and Clinical Neuroscience Karolinska Institutet, Stockholm, Sweden

***Supplementary Material***

Table of Contents:

[Supplementary Table 1. Search terms 7](#_Toc126925782)

[Supplementary Table 2. Studies excluded for potential population selection bias 8](#_Toc126925783)

[Supplementary Table 3. Study and patient baseline characteristics at the study level 9](#_Toc126925784)

[Supplementary Table 4. Combined (EmboTrap, Trevo, and Solitaire) ordinal mRS scores across treatment groups. 12](#_Toc126925785)

[Supplementary Results 1: Ordinal mRS scores at 90 days 13](#_Toc126925786)

[Supplementary Results 2: Core-lab subanalysis: 13](#_Toc126925788)

[Supplementary Results 3: Prospective-only subanalysis: 14](#_Toc126925789)

[Supplementary Table 5. Comparison of recanalization outcomes between EmboTrap^®^, Trevo, and Solitaire among studies with outcomes adjudicated by a core-laboratory. 16](#_Toc126925790)

[Supplementary Table 6. Comparisons of functional, safety, and recanalization outcomes between EmboTrap®, Trevo, and Solitaire among prospective studies. 18](#_Toc126925791)

[Supplementary Figure 1. Outlier and influence analyses of rates of mRS 0-2 at 90 days 20](#_Toc126925792)

[Supplementary Figure 2. Forest plot of comparisons of mRS 0-2 at 90 days. 22](#_Toc126925793)

[Supplementary Figure 3. Forest plot of comparisons of mRS 0-2 at 90 days. . 23](#_Toc126925794)

[Supplementary Figure 4. Outlier and influence analyses of rates of mortality at 90 days 24](#_Toc126925795)

[Supplementary Figure 5. Forest plot of comparisons of mortality at 90 days. 26](#_Toc126925796)

[Supplementary Figure 6. Forest plot of comparisons of mortality at 90 days 27](#_Toc126925797)

[Supplementary Figure 7. Outlier and influence analyses of ENT rates 28](#_Toc126925798)

[Supplementary Figure 8. Forest plot of comparisons of ENT/distal emboli. 30](#_Toc126925799)

[Supplementary Figure 9. Outlier and influence analyses of sICH rates 31](#_Toc126925800)

[Supplementary Figure 10. Forest plot of comparisons of sICH. 33](#_Toc126925801)

[Supplementary Figure 11. Forest plot of comparisons of complete or near-complete recanalization on first pass (FPR mTICI ≥2c). 34](#_Toc126925802)

[Supplementary Figure 12. Forest plot of comparisons of successful recanalization on first pass (mFPR mTICI ≥2b). 35](#_Toc126925803)

[Supplementary Figure 13. Forest plot of comparisons of final complete recanalization (TICI 3). 36](#_Toc126925804)

[Supplementary Figure 14. Forest plot of comparisons of final successful recanalization (mTICI ≥2b). 37](#_Toc126925805)

[Supplementary Figure 15. Forest plot of comparisons of complete or near-complete recanalization on first pass (FPR mTICI ≥2c) among core-lab adjudicated studies. 38](#_Toc126925806)

[Supplementary Figure 16. Forest plot of comparisons of successful recanalization on first pass (mFPR mTICI ≥2b) among core-lab adjudicated studies. 38](#_Toc126925807)

[Supplementary Figure 17. Forest plot of comparisons of final complete recanalization (TICI 3) among core-lab adjudicated studies. 39](#_Toc126925808)

[Supplementary Figure 18. Forest plot of comparisons of final successful recanalization (mTICI ≥2b) among core-lab adjudicated studies 40](#_Toc126925809)

[Supplementary Figure 19. Forest plot of comparisons of successful recanalization on first pass (mFPR mTICI ≥2b) among prospective studies. 41](#_Toc126925810)

[Supplementary Figure 20. Forest plot of comparisons of final complete recanalization (TICI 3) among prospective studies. 42](#_Toc126925811)

[Supplementary Figure 21. Forest plot of comparisons of final successful recanalization (mTICI ≥2b) among prospective studies. 43](#_Toc126925812)

[Supplementary Figure 22. Forest plot of comparisons of ENT/distal emboli among prospective studies. 44](#_Toc126925813)

[Supplementary Figure 23. Forest plot of comparisons of sICH among prospective studies. 45](#_Toc126925814)

[Supplementary Figure 24. Forest plot of comparisons of mRS 0-2 at 90 days among prospective studies. 46](#_Toc126925815)

[Supplementary Figure 25. Forest plot of comparisons of mortality at 90 days among prospective studies. 47](#_Toc126925816)

## **Supplemen**tary Table 1. Search terms

| **Search term** | **Database** |
| --- | --- |
| (AIS OR "ischemic stroke") AND (Solitaire OR Trevo) | PubMed |
| "ischemic stroke" AND (embotrap OR stent-retriever) | PubMed |
| (LVO OR "large vessel occlusion") AND (FPE OR "first pass effect" OR "first pass efficacy" OR "first pass success" OR "first pass recanalization") | PubMed |
| (AIS OR "acute ischemic stroke") AND (FPE OR "first pass effect" OR "first pass efficacy" or "first pass success") | PubMed Central |
| ("cerebral infarction" OR "clot retrieval" OR "balloon guide catheter" OR BGC OR "distal emboli") AND (FPE OR "first pass effect" OR "first pass efficacy" or "first pass success" or "first pass reperfusion" or "first pass recanalization") | PubMed |
| embotrap | PubMed |
| ("cerebral infarction" OR "clot retrieval" OR "balloon guide catheter" OR BGC OR "distal emboli") AND (FPE OR "first pass effect" OR "first pass efficacy" or "first pass success" or "first pass reperfusion" or "first pass recanalization") | PubMed Central |
| (one-pass thrombectomy) OR (first-attempt recanalization) | PubMed Central |
| ("stent-retriever" OR "retriever" OR "thrombectomy") AND (FPE OR "first pass effect" OR "first pass efficacy" OR "first pass success") | PubMed Central |
| (LVO OR "large vessel occlusion" OR reperfusion OR recanalization) AND (FPE OR "first pass effect" OR "first pass efficacy" or "first pass success") | PubMed Central |
| (one-pass thrombectomy) OR (first-attempt recanalization) | PubMed |
| (LVO OR "large vessel occlusion" OR reperfusion OR recanalization) AND (FPE OR "first pass effect" OR "first pass efficacy" or "first pass success") | PubMed |
| ("stent-retriever" OR "retriever" OR "thrombectomy") AND (FPE OR "first pass effect" OR "first pass efficacy" OR "first pass success") | PubMed |
| (AIS OR "acute ischemic stroke") AND (FPE OR "first pass effect" OR "first pass efficacy" or "first pass success") | PubMed |

## Supplementary Table 2. Twenty-two Studies excluded for potential population selection bias [(abstract review (n = 20); full text review (n = 2)]

| **Excluded Study** | **Exclusion Reason** |
| --- | --- |
| Monayao et al. Increased incidence and treatment of intracranial atherosclerotic disease during mechanical thrombectomy is safe, even with an increased number of passes. *Journal of neurointerventional surgery.* 2021. | Intracranial Atherosclerotic Disease (ICAD) patients only |
| Vollherbst et al. Concomitant Acute Ischemic Stroke and Upper Extremity Arterial Occlusion: Feasibility of Mechanical Thrombectomy of the Upper Limb Using Neurointerventional Devices and Techniques. *J Clin Med.* 2021. | Craniocervical occlusions only |
| Onodera et al. A Direct Aspiration First Pass Technique for Vertebra-Basilar Occlusion: A Retrospective Comparison to Stent Retriever. *J Stroke Cerebrovasc Dis.* 2021. | Vertebrobasilar occlusions only |
| Okawa et al. Early Loss of Immediate Reperfusion While Stent Retriever in Place Predicts Successful Final Reperfusion in Acute Ischemic Stroke Patients. *Stroke.* 2015. | Reperfused patients only |
| Fahed et al. A leap forward in the endovascular management of acute basilar artery occlusion since the appearance of stent retrievers: a single-center comparative study. *J Neurosurg.* 2017. | Vertebrobasilar occlusions only |
| Lee et al. Acute Basilar Artery Occlusion: Differences in Characteristics and Outcomes after Endovascular Therapy between Patients with and without Underlying Severe Atherosclerotic Stenosis. *AJNR Am J Neuroradiol.* 2017. | Vertebrobasilar occlusions only |
| Son et al. Comparison of Solitaire thrombectomy and Penumbra suction thrombectomy in patients with acute ischemic stroke caused by basilar artery occlusion. *J Neurointerv Surg.* 2016. | Vertebrobasilar occlusions only |
| Son et al. Emergency carotid artery stenting in patients with acute ischemic stroke due to occlusion or stenosis of the proximal internal carotid artery: a single-center experience. *J Neurointerv Surg.* 2015. | Intracranial Atherosclerotic Disease (ICAD) patients only |
| Bernsen et al. Aspiration Versus Stent Retriever Thrombectomy for Posterior Circulation Stroke. *Stroke.* 2021. | Vertebrobasilar occlusions only |
| Yi et al. Microcatheter "First-Pass Effect" Predicts Acute Intracranial Artery Atherosclerotic Disease-Related Occlusion. *Neurosurgery.* 2019. | Intracranial Atherosclerotic Disease (ICAD) patients only |
| Kim et al. Endovascular Treatment After Stroke Due to Large Vessel Occlusion for Patients Presenting Very Late From Time Last Known Well. *JAMA Neurol.* 2020. | Only late window patients (>24 hours from onset) |
| Ozaki et al. Endovascular Therapy of Acute Ischemic Stroke in Patients with Large-Vessel Occlusion Associated with Active Malignancy. *J Stroke Cerebrovasc Dis.* 2021. | Cancer patients only |
| Aubertin et al. First-Pass Effect in Basilar Artery Occlusions: Insights From the Endovascular Treatment of Ischemic Stroke Registry. *Stroke.* 2021. | Vertebrobasilar occlusions only |
| Song et al. Impact of temporary opening using a stent retriever on clinical outcome in acute ischemic stroke. *PLoS One.* 2015. | Reperfused patients only |
| Diogo C. et al. Functional Independence following Endovascular Treatment for Basilar Artery Occlusion despite Extensive Bilateral Pontine Infarcts on Diffusion-Weighted Imaging: Refuting a Self-Fulfilling Prophecy. *Interventional Neurology.* 2016. | Vertebrobasilar occlusions only |
| Anadani et al. Endovascular therapy with or without intravenous thrombolysis in acute stroke with tandem occlusion. *J Neurointerv Surg.* 2021. | Tandem occlusions only |
| Lareyre et al. Patterns of Acute Ischemic Strokes After Carotid Endarterectomy and Therapeutic Implications. *Vasc Endovascular Surg.* 2017. | Stroke treatment as a complication of Carotid Endarterectomy |
| Gory et al. Predictors for Mortality after Mechanical Thrombectomy of Acute Basilar Artery Occlusion. *Cerebrovasc Dis.* 2018. | Vertebrobasilar occlusions only |
| Huijun et al. Intracranial Atherosclerotic Disease-Related Acute Middle Cerebral Artery Occlusion Can Be Predicted by Diffusion-Weighted Imaging. *Frontiers in Neuroscience.* 2019. | Intracranial Atherosclerotic Disease (ICAD) patients only |
| Baik et al. Mechanical thrombectomy for acute posterior cerebral artery stroke; Feasibility and predictors of outcome. *Neuroradiology.* 2022. | Vertebrobasilar occlusions only |
| Cunha et al. Acute Treatment of Isolated Posterior Cerebral Artery Occlusion: Single Center Experience. *J Stroke Cerebrovasc Dis.* 2022. | Posterior Inferior Cerebellar  Artery occlusions only |
| Kurmann et al. Heterogeneity of the Relative Benefits of TICI 2c/3 over TICI 2b50/2b67: Are there Patients who are less Likely to Benefit?. *Clin Neuroradiol.* 2022. | Reperfused patients only |

## Supplementary Table 3. Study and patient baseline characteristics at the study level

| **Author, Year** | **Study Type** | | | **Core vs. Site** | **Device** | | **N** | **Use of BGCs** | | **Use of IVT** | | **sICH* Definition** | | **Age** | **Baseline ASPECTS** | **Baseline NIHSS** | **Baseline mRS** | | **Stroke Location** | | | | | | | | | | | | | | |
| --- | --- | --- | --- | --- | --- | --- | --- | --- | --- | --- | --- | --- | --- | --- | --- | --- | --- | --- | --- | --- | --- | --- | --- | --- | --- | --- | --- | --- | --- | --- | --- | --- | --- |
|  |  |  |  |  |  |  |  |  |  |  |  |  |  |  |  |  | **0** | **1** | **Anterior** | **Posterior** | **Tandem** | **ACA** | **A1** | **A2** | **A3** | **BA** | **ICA** | **MCA** | **M1** | **M2** | **M3** | **PCA** | **VA** |
| Bourcier et al., 2018[43] | Retrospective | | | Site | Embotrap | | 80 | 37 (46.3%) | | 45 (56.3%) | | ECASS III | | 72 (N/A) | 8 (N/A) | 15 (N/A) | N/A | | 80 (100%) | 0 (0%) | 7 (8.7%) | 0 (0%) | 0 (0%) | 0 (0%) | 0 (0%) | 0 (0%) | 19 (23.7%) | 79 (98.7%) | N/A | N/A | 0 (0%) | 0 (0%) | 0 (0%) |
| Brouwer et al., 2018[33] | Prospective | | | Core | Embotrap | | 201 | N/A | | 95 (47.3%) | | ECASS III | | 67.2±12.7 | N/A | 15 (N/A) | N/A | | 177 (88.1%) | 24 (11.9%) | N/A | 1 (0.5%) | N/A | N/A | N/A | 0 (0%) | 53 (26.4%) | 123 (61.2%) | N/A | N/A | N/A | 24 (11.9%) | 0 (0%) |
| Kabbasch et al., 2016[53] | Retrospective | | | Site | Embotrap | | 40 | 17 (42.5%) | | 26 (65.0%) | | ECASS III | | N/A | N/A | 16 (5-25) | N/A | | 35 (87.5%) | 5 (12.5%) | 8 (20.0%) | 0 (0%) | 0 (0%) | 0 (0%) | 0 (0%) | 4 (10.0%) | 2 (5.0%) | 25 (62.5%) | 23 (57.5%) | 2 (5.0%) | 0 (0%) | 1 (2.5%) | 0 (0%) |
| Mattle et al., 2018[35] | Prospective | | | Site | Embotrap | | 40 | N/A | | 31 (77.5%) | | SITS-MOST | | 64±14.2 | N/A | 15.5±4.52 | N/A | | 40 (100%) | 0 (0%) | 0 (0%) | 0 (0%) | 0 (0%) | 0 (0%) | 0 (0%) | 0 (0%) | 6 (15.0%) | 34 (85.0%) | 29 (72.5%) | 5 (12.5%) | 0 (0%) | 0 (0%) | 0 (0%) |
| Srivatsan et al., 2021[30] | Prospective | | | Site | Embotrap | | 70 | 27 (38.6%) | | 24 (34.3%) | | Not defined in study | | 69.9±16.5 | N/A | 16.3±6.6 | N/A | | 69 (98.6%) | 1 (1.4%) | 3 (4.3%) | 0 (0%) | 0 (0%) | 0 (0%) | 0 (0%) | 1 (1.4%) | 15 (21.4%) | 51 (72.9%) | 38 (54.3%) | 11 (15.7%) | 2 (2.9%) | 0 (0%) | 0 (0%) |
| Valente et al., 2019[63] | Retrospective | | | Site | Embotrap | | 29 | 23 (79.3%) | | 21 (72.4%) | | Not defined in study | | 77±9.39 | 8.1±1.45 | 18.48±5.37 | N/A | | 29 (100%) | 0 (0%) | 0 (0%) | 0 (0%) | 0 (0%) | 0 (0%) | 0 (0%) | 0 (0%) | 4 (13.8%) | 25 (86.2%) | 22 (75.9%) | 3 (10.3%) | 0 (0%) | 0 (0%) | 0 (0%) |
| Zaidat et al., 2018[7] | Prospective | | | Core | Embotrap | | 227 | 167 (73.6%) | | 120 (52.9%) | | ECASS III | | 68±13 | 9.2±1.5  10 (9-10) | 15.8±5  16 (12-19) | 177 (78.0%) | 49 (22.6%) | 218 (96.0%) | 9 (4.0%) | 0 (0%) | 0 (0%) | 0 (0%) | 0 (0%) | 0 (0%) | 0 (0%) | 35 (15.4%) | 183 (80.6%) | 126 (55.5%) | 57 (25.1%) | 0 (0%) | 9 (4.0%) | 0 (0%) |
| Baek et al., 2021[42] | Retrospective | | | Site | Solitaire | | 79 | N/A | | 27 (34.2%) | | ECASS II | | 69±N/A | N/A | 15 (12-19) | N/A | | 79 (100%) | 0 (0%) | 0 (0%) | 0 (0%) | 0 (0%) | 0 (0%) | 0 (0%) | 0 (0%) | 25 (31.6%) | 54 (68.4%) | 46 (58.2%) | 8 (10.1%) | 0 (0%) | 0 (0%) | 0 (0%) |
|  |  |  |  |  | Trevo | | 51 | N/A | | 16 (31.4%) | |  | | 67±N/A | N/A | 14 (11-17) | N/A | | 51 (100%) | 0 (0%) | 0 (0%) | 0 (0%) | 0 (0%) | 0 (0%) | 0 (0%) | 0 (0%) | 16 (31.4%) | 35 (68.6%) | 30 (58.8%) | 5 (9.8%) | 0 (0%) | 0 (0%) | 0 (0%) |
| Liang et al., 2020[57] | Retrospective | | | Site | Solitaire | | 73 | N/A | | N/A | | sICH not reported | | 66.8±14.1 | N/A | 17±7 | N/A | | N/A | N/A | N/A | | | | | | | | | | | | |
|  |  |  |  |  | Trevo | | 55 | N/A | | N/A | |  | | 69.6±16 | N/A | 18±8 | N/A | | N/A | N/A | N/A | | | | | | | | | | | | |
| Pu et al.[36] | Prospective | | | Site | Solitaire | | 32 | N/A | | 32 (100%) | | Not defined in study | | 63.87±4.13 | N/A | N/A | N/A | | 29 (93.5%) | 2 (6.5%) | 0 (0%) | N/A | N/A | N/A | N/A | 2 (6.5%) | 8 (25.8%) | N/A | 16 (51.6%) | 5 (16.1%) | N/A | N/A | N/A |
|  |  |  |  |  | Trevo | | 30 | N/A | | 30 (100%) | |  | | 68.87±7.69 | N/A | N/A | N/A | | 24 (96.0%) | 1 (4.0%) | 0 (0%) | N/A | N/A | N/A | N/A | 1 (4.0%) | 6 (24.0%) | N/A | 15 (60.0%) | 3 (12.0%) | N/A | N/A | N/A |
| Yi et al., 2018[67] | Retrospective | | | Site | Solitaire | | 102 | 67 (65.7%) | | 68 (66.7%) | | sICH not reported | | 64.3± N/A | N/A | 11.3±N/A | N/A | | 102 (100%) | 0 (0%) | 0 (0%) | 0 (0%) | 0 (0%) | 0 (0%) | 0 (0%) | 0 (0%) | 34 (33.3%) | 68 (66.7%) | 60 (58.8%) | 8 (7.8%) | 0 (0%) | 0 (0%) | 0 (0%) |
|  |  |  |  |  | Trevo | | 98 | 69 (70.4%) | | 59 (60.2%) | |  | | 67.9± N/A | N/A | 11.7±N/A | N/A | | 98 (100%) | 0 (0%) | 0 (0%) | 0 (0%) | 0 (0%) | 0 (0%) | 0 (0%) | 0 (0%) | 25 (25.5%) | 73 (74.5%) | 61 (62.2%) | 12 (12.2%) | 0 (0%) | 0 (0%) | 0 (0%) |
| Baek et al., 2017[41] | Retrospective | | | Site | Solitaire | | 165 | 84 (50.9%) | | 44 (26.7%) | | sICH not reported | | 70.3±11.2 | N/A | N/A | N/A | | 141 (85.5%) | 24 (14.5%) | 0 (0%) | 0 (0%) | 0 (0%) | 0 (0%) | 0 (0%) | 23 (13.9%) | 46 (27.9%) | 95 (57.6%) | 81 (49.1%) | 14 (8.5%) | 0 (0%) | 1 (0.6%) | 0 (0%) |
| Cabral et al., 2016[27] | Prospective | | | Site | Solitaire | | 31 | N/A | | 25 (78.1%) | | ECASS II | | 61.7±11.6 | 10 (N/A) | 19 (17-24) | N/A | | 26 (83.9%) | 5 (16.1%) | 0 (0%) | 0 (0%) | 0 (0%) | 0 (0%) | 0 (0%) | 5 (16.1%) | 13 (41.9%) | 13 (41.9%) | 13 (41.9%) | 0 (0%) | 0 (0%) | 0 (0%) | 0 (0%) |
| Campbell et al., 2015[21] | Prospective | | | Core | Solitaire | | 35 | N/A | | 35 (100%) | | SITS-MOST | | 68.6±12.3 | N/A | 17 (13-20) | N/A | | 35 (100%) | 0 (0%) | 0 (0%) | 0 (0%) | 0 (0%) | 0 (0%) | 0 (0%) | 0 (0%) | 11 (31.4%) | 24 (68.6%) | 20 (57.1%) | 4 (11.4%) | 0 (0%) | 0 (0%) | 0 (0%) |
| Cao et al., 2020[22] | Prospective | | | Core | Solitaire | | 69 | N/A | | 23 (33.3%) | | ECASS III | | 67.19±10.23 | N/A | 17.62±4.33 | 66 (95.7%) | 2 (2.9%) | 61 (88.4%) | 8 (11.6%) | 0 (0%) | 0 (0%) | 0 (0%) | 0 (0%) | 0 (0%) | 7 (10.1%) | 9 (13.0%) | 52 (75.4%) | 46 (66.7%) | 6 (8.7%) | 0 (0%) | 0 (0%) | 1 (1.4%) |
| Choi et al., 2015[44] | Retrospective | | | Site | Solitaire | | 32 | 0 (0%) | | 20 (62.5%) | | ECASS III | | 64.19±9.09 | N/A | 14.5 (11-17) | N/A | | 32 (100%) | 0 (0%) | 0 (0%) | 0 (0%) | 0 (0%) | 0 (0%) | 0 (0%) | 0 (0%) | 8 (25.0%) | 24 (75.0%) | N/A | N/A | 0 (0%) | 0 (0%) | 0 (0%) |
| Guo et al., 2019[45] | Retrospective | | | Core | Solitaire | | 251 | N/A | | 82 (32.7%) | | Heidelberg Bleeding Classification | | 64.4±11.8 | N/A | 16 (12-20) | N/A | | 251 (100%) | 0 (0%) | N/A | | | | | | | | | | | | |
| He et al., 2022[47] | Retrospective | | | Site | Solitaire | | 246 | 0 (0%) | | 102 (41.5%) | | Not defined in study | | 71.63±11.65 | 9.63±0.79 | 16.85±5.36 | N/A | | 246 (100%) | 0 (0%) | 0 (0%) | 0 (0%) | 0 (0%) | 0 (0%) | 0 (0%) | 0 (0%) | 77 (31.3%) | 169 (68.7%) | 149 (60.6%) | 20 (8.1%) | 0 (0%) | 0 (0%) | 0 (0%) |
| Hesse et al., 2018[48] | Retrospective | | | Site | Solitaire | | 102 | 102 (100%) | | N/A | | Not defined in study | | 74 (67-80) | 8 (7-9) | 15.5±5.5 | N/A | | 102 (100%) | 0 (0%) | 0 (0%) | 0 (0%) | 0 (0%) | 0 (0%) | 0 (0%) | 0 (0%) | 27 (26.5%) | 75 (73.5%) | 66 (64.7%) | 9 (8.8%) | 0 (0%) | 0 (0%) | 0 (0%) |
| Jiang et al., 2019[34] | Prospective | | | Site | Solitaire | | 37 | 0 (0%) | | 13 (35.1%) | | ECASS III | | 74 (67-80) | 10 (8-10) | 15 (12-19) | N/A | | 37 (100%) | 0 (0%) | 0 (0%) | 0 (0%) | 0 (0%) | 0 (0%) | 0 (0%) | 0 (0%) | 0 (0%) | 37 (100%) | 0 (0%) | 37 (100%) | 0 (0%) | 0 (0%) | 0 (0%) |
| Jiang et al., 2015[51] | Retrospective | | | Site | Solitaire | | 89 | 27 (30.3%) | | 25 (28.1%) | | SWIFT | | 63.12±13.98 | N/A | 19.17±4.64  19 (N/A) | N/A | | 81 (91.0%) | 7 (7.9%) | 16 (18.0%) | 2 (2.2%) | N/A | N/A | N/A | 4 (4.5%) | 4 (4.5%) | 62 (69.7%) | N/A | N/A | 0 (0%) | 0 (0%) | 1 (1.1%) |
| Jiang et al., 2016[82] | Retrospective | | | Site | Solitaire | | 83 | 26 (31.3%) | | 30 (36.1%) | | Not defined in study | | 63.34±13.7 | N/A | 19.12±4.6 | N/A | | 75 (90.4%) | 8 (9.6%) | 12 (14.5%) | 0 (0%) | 0 (0%) | 0 (0%) | 0 (0%) | 2 (2.4%) | 6 (7.2%) | 57 (68.7%) | N/A | N/A | N/A | 0 (0%) | 6 (7.2%) |
| Jovin et al., 2015[23] | Prospective | | | Core | Solitaire | | 103 | 62 (60.2%) | | 70 (68.0%) | | ECASS II | | 65.7±11.3  67 (58-76) | 7 (6-9) | 17 (14-20) | N/A | | 103 (100%) | 0 (0%) | 0 (0%) | 0 (0%) | 0 (0%) | 0 (0%) | 0 (0%) | 0 (0%) | 25 (24.5%) | 73 (71.6%) | 65 (63.7%) | 7 (6.9%) | 1 (1.0%) | 0 (0%) | 0 (0%) |
| Kaesmacher et al., 2019[28] | Prospective | | | Site | Solitaire | | 1616 | N/A | | N/A | | ECASS II | | N/A | N/A | N/A | N/A | | 1616 (100%) | 0 (0%) | N/A | | | | | | | | | | | | |
| Kammerer et al., 2018[54] | Retrospective | | | Site | Solitaire | | 170 | 170 (100%) | | N/A | | Not defined in study | | 71±N/A | N/A | N/A | N/A | | 170 (100%) | 0 (0%) | 22 (15.3%) | N/A | N/A | N/A | N/A | N/A | 4 (2.8%) | 118 (81.9%) | N/A | N/A | 0 (0%) | N/A | N/A |
| Lapergue et al., 2016[29] | Prospective | | | Site | Solitaire | | 119 | 119 (100%) | | N/A | | ECASS II | | 65.5±14.7 | 8 (7-10) | 15.9±6.1 | N/A | | 119 (100%) | 0 (0%) | N/A | 0 (0%) | 0 (0%) | 0 (0%) | 0 (0%) | 0 (0%) | 32 (26.9%) | 87 (73.1%) | N/A | N/A | N/A | 0 (0%) | 0 (0%) |
| Li et al., 2019[56] | Retrospective | | | Site | Solitaire | | 174 | N/A | | 18 (10.3%) | | Heidelberg Bleeding Classification | | 68.63±11.12 | N/A | N/A | N/A | | 174 (100%) | 0 (0%) | 0 (0%) | 0 (0%) | 0 (0%) | 0 (0%) | 0 (0%) | 0 (0%) | 71 (40.8%) | 103 (59.2%) | 103 (59.2%) | 0 (0%) | 0 (0%) | 0 (0%) | 0 (0%) |
| Mokin et al., 2015[58] | Retrospective | | | Site | Solitaire | | 41 | N/A | | 13 (31.7%) | | sICH not reported | | 68.4±12.53 | N/A | 16.75±4.2 | N/A | | 41 (100%) | 0 (0%) | N/A | | | | | | | | | | | | |
| Pfaff et al., 2019[61] | Retrospective | | | Site | Solitaire | | 75 | N/A | | 41 (54.7%) | | Heidelberg Bleeding Classificatio | | 75±12 | 8 (7-9) | 17 (11-21) | N/A | | 75 (100%) | 0 (0%) | 5 (6.7%) | 0 (0%) | 0 (0%) | 0 (0%) | 0 (0%) | 0 (0%) | 12 (16.0%) | 58 (77.3%) | 42 (56.0%) | 16 (21.3%) | 0 (0%) | 0 (0%) | 0 (0%) |
| Sang et al., 2019[37] | Prospective | | | Site | Solitaire | | 48 | 6 (12.5%) | | 10 (20.8%) | | ECASS III | | 70.5 (62-80) | 9 (8-10) | 22 (12.5-26) | N/A | | 0 (0%) | 48 (100%) | 0 (0%) | 0 (0%) | 0 (0%) | 0 (0%) | 0 (0%) | 43 (89.6%) | 0 (0%) | 0 (0%) | 0 (0%) | 0 (0%) | 0 (0%) | 0 (0%) | 5 (10.4%) |
| Saver et al., 2015[25] | Prospective | | | Core | Solitaire | | 98 | N/A | | 98 (100%) | | SWIFT | | 65±12.5 | 8 (7-10) | 16.7±4.5  17 (13-20) | 81 (82.7%) | 15 (15.3%) | 98 (100%) | 0 (0%) | N/A | N/A | N/A | N/A | N/A | N/A | 17 (18.3%) | N/A | 62 (66.7%) | 13 (14.0%) | N/A | N/A | N/A |
| Slezak et al., 2017[38] | Prospective | | | Site | Solitaire | | 401 | N/A | | 122 (30.4%) | | ECASS II and PROACT II | | 70.77±14.23 | N/A | 15.82±6.6 | N/A | | 401 (100%) | 0 (0%) | 0 (0%) | 1 (0.2%) | 1 (0.2%) | 0 (0%) | 0 (0%) | 0 (0%) | 137 (34.2%) | 263 (65.6%) | N/A | N/A | 0 (0%) | 0 (0%) | 0 (0%) |
| Sztajzel et al., 2015[62] | Retrospective | | | Site | Solitaire | | 63 | 63 (100%) | | 63 (100%) | | ECASS II | | 68.3±N/A | N/A | 15.7±N/A | N/A | | 63 (100%) | 0 (0%) | 0 (0%) | 0 (0%) | 0 (0%) | 0 (0%) | 0 (0%) | 0 (0%) | 11 (17.5%) | 52 (82.5%) | 42 (66.7%) | 10 (15.9%) | 0 (0%) | 0 (0%) | 0 (0%) |
| Wiącek et al., 2017[64] | Retrospective | | | Site | Solitaire | | 43 | 37 (86.0%) | | 28 (30.4%) | | SITS-MOST | | 75 (N/A) | N/A | 16.4±N/A | N/A | | 37 (86.0%) | 6 (14.0%) | 1 (2.3%) | 1 (2.3%) | N/A | N/A | N/A | 4 (9.3%) | 6 (13.9%) | 31 (72.0%) | 23 (53.4%) | 7 (16.3%) | 1 (2.3%) | 2 (4.7%) | 0 (0%) |
| Yang et al., 2021[66] | Retrospective | | | Site | Solitaire | | 43 | N/A | | 0 (0%) | | sICH not reported | | 58.7±3.4 | N/A | 10.44±2.21 | N/A | | N/A | N/A | N/A | | | | | | | | | | | | |
| Yang et al., 2017[65] | Retrospective | | | Site | Solitaire | | 628 | N/A | | 200 (31.8%) | | Heidelberg Bleeding Classification | | 66±N/A | N/A | 17 (N/A) | N/A | | 628 (100%) | 0 (0%) | 0 (0%) | 3 (0.5%) | 0 (0%) | 0 (0%) | 0 (0%) | 0 (0%) | 241 (38.4%) | 384 (61.1%) | N/A | N/A | N/A | 0 (0%) | 0 (0%) |
| Yi et al., 2018[68] | Retrospective | | | Site | Solitaire | | 56 | 0 (0%) | | 17 (30.4%) | | SITS-MOST | | 65±11 | N/A | 18±4  18 (10-28) | N/A | | 56 (100%) | 0 (0%) | 0 (0%) | 0 (0%) | 0 (0%) | 0 (0%) | 0 (0%) | 0 (0%) | 25 (44.6%) | 31 (55.4%) | 31 (55.4%) | 0 (0%) | 0 (0%) | 0 (0%) | 0 (0%) |
| Zaidat et al., 2018[31] | Prospective | | | Site | Solitaire | | 354 | N/A | | N/A | | SWIFT | | 67.3±15.2 | N/A | 18.1±6.6 | N/A | | 318 (89.8%) | 36 (10.2%) | N/A | N/A | N/A | N/A | N/A | 36 (10.2%) | 82 (23.2%) | 197 (55.6%) | 197 (55.6%) | N/A | N/A | N/A | N/A |
| Zaidat et al., 2019[32] | Prospective | | | Core | Solitaire | | 62 | 0 (0%) | | 43 (69.4%) | | SWIFT | | 70.1±15.4  70.5 (63-82) | 8.4±1.4  9 (8-9) | 17.4±5.6  17.5 (14-22) | 48 (77.4%) | 10 (16.1%) | 62 (100%) | 0 (0%) | 0 (0%) | 0 (0%) | 0 (0%) | 0 (0%) | 0 (0%) | 0 (0%) | 14 (22.6%) | 48 (77.4%) | 33 (53.2%) | 15 (24.2%) | 0 (0%) | 0 (0%) | 0 (0%) |
| Zhou et al., 2018[69] | Retrospective | | | Site | Solitaire | | 73 | N/A | | 8 (11.0%) | | PROACT II | | 58.72±4.54 | N/A | 17.08±4.02 | N/A | | 73 (100%) | 0 (0%) | 12 (16.4%) | 0 (0%) | 0 (0%) | 0 (0%) | 0 (0%) | 0 (0%) | 26 (35.6%) | 35 (47.9%) | N/A | N/A | N/A | 0 (0%) | 0 (0%) |
| Zhou et al., 2021[39] | Prospective | | | Core | Solitaire | | 97 | N/A | | 23 (23.7%) | | Not defined in study | | 64.57±12.47 | 11.56±2.15 | 15.77±5.17 | N/A | | 97 (100%) | 0 (0%) | 0 (0%) | 0 (0%) | 0 (0%) | 0 (0%) | 0 (0%) | 0 (0%) | 22 (22.7%) | 77 (79.4%) | 68 (70.1%) | 9 (9.3%) | 0 (0%) | 0 (0%) | 0 (0%) |
| Binning et al., 2018[26] | Prospective | | | Core and Site | Trevo | | Total: 2008, Core: 1,599* | 1004 (50.6%) | | 1041 (52.3%) | | ECASS III | | 68±14 | 8 (7-9) | 15.5±6.8 | 1372 (70.5%) | 290 (14.9%) | 1859/2002 (92.9%) | 143/2002  (7.1%) | 0 (0%) | 11 (0.5%) | 3 (0.1%) | 8 (0.4%) | 0 (0%) | 0 (0%) | 356 (17.8%) | 1492 (74.5%) | 1096 (54.7%) | 375 (18.7%) | 21 (1.0%) | 0 (0%) | 143 (7.1%) |
| Haussen et al., 2020[46] | Retrospective | | | Site | Trevo | | 92 | 86 (93.5%) | | 36 (39.1%) | | sICH not reported | | 66 (55-74) | 9 (7-10) | 16 (11-23) | N/A | | 85 (92.3%) | 7 (7.6%) | 5 (5.4%) | 19 (20.7%) | 1 (1.1%) | 7 (7.6%) | 11 (12.0%) | 0 (0%) | 0 (0%) | 61 (66.3%) | 0 (0%) | 51 (55.4%) | 15 (16.3%) | 7 (7.6%) | 0 (0%) |
| Imahori et al., 2017[50] | Prospective | | | Site | Trevo | | 50 | 50 (100%) | | 9 (18.0%) | | ECASS III | | 80 (72-86) | 8 (7-9) | 20 (11-23) | N/A | | 50 (100%) | 0 (0%) | 0 (0%) | 0 (0%) | 0 (0%) | 0 (0%) | 0 (0%) | 0 (0%) | 13 (26.0%) | 37 (74.0%) | 21 (42.0%) | 16 (32.0%) | 0 (0%) | 0 (0%) | 0 (0%) |
| Imahori et al., 2018[49] | Retrospective | | | Site | Trevo | | 50 | 50 (100%) | | 8 (16.0%) | | ECASS III | | 79 (73-85) | 9 (8-10) | 15 (7-22) | N/A | | 50 (100%) | 0 (0%) | 0 (0%) | 0 (0%) | 0 (0%) | 0 (0%) | 0 (0%) | 0 (0%) | 0 (0%) | 50 (100%) | 50 (100%) | 0 (0%) | 0 (0%) | 0 (0%) | 0 (0%) |
| Imahori et al., 2017[40] | Retrospective | | | Site | Trevo | | 80 | 80 (100%) | | 18 (22.5%) | | ECASS III | | 79 (73-85) | 8 (7-10) | 15 (8-22) | N/A | | 80 (100%) | 0 (0%) | 0 (0%) | 0 (0%) | 0 (0%) | 0 (0%) | 0 (0%) | 0 (0%) | 15 (18.8%) | 65 (81.3%) | 41 (51.3%) | 24 (30.0%) | 0 (0%) | 0 (0%) | 0 (0%) |
| Kühn et al., 2017[55] | Retrospective | | | Site | Trevo | | 35 | N/A | | 16 (45.7%) | | Not defined in study | | 66.1±N/A | N/A | 18.23±5.83  19 (15-22.5) | 29 (82.9%) | 3 (8.6%) | 28 (80.0%) | 7 (20%) | 3 (8.6%) | 3 (8.6%) | 0 (0%) | 3 (8.6%) | 0 (0%) | 0 (0%) | 0 (0%) | 28 (80.0%) | 8 (22.9%) | 20 (57.1%) | 0 (0%) | 4 (11.4%) | 3 (8.6%) |
| Kabbasch et al., 2016[52] | Retrospective | | | Site | Trevo | | 76 | N/A | | N/A | | ECASS III | | 75.2±12.9 | N/A | 18 (N/A) | N/A | | 63 (82.9%) | 13 (17.1%) | 0 (0%) | 1 (1.3%) | 0 (0%) | 1 (1.3%) | 0 (0%) | N/A | 10 (13.2%) | 52 (68.4%) | 44 (57.9%) | 8 (10.5%) | 0 (0%) | 0 (0%) | 13 (17.1%) |
| Mokin et al., 2020[59] | Retrospective | | | Site | Trevo | | 609 | 288 (47.3%) | | 307 (50.4%) | | SITS-MOST | | 66.2±14.7 | N/A | 17.33±6.67 | 442 (78.8%) | 53 (9.4%) | 520 (86.2%) | 82 (13.5%) | 6 (1.0%) | 5 (0.8%) | N/A | N/A | N/A | 63 (10.4%) | 97 (16.0%) | 418 (68.8%) | 333 (54.8%) | 79 (13.0%) | 6 (1.0%) | 4 (0.7%) | 15 (2.5%) |
| Nogueira et al., 2018[24] | Prospective | | | Core | Trevo | | 107 | N/A | | 5 (4.6%) | | ECASS III | | 69.4±14.1 | N/A | 17.4±5.1  17 (13-21) | 99 (92.5%) | N/A | 107 (100%) | 0 (0%) | 0 (0%) | 0 (0%) | 0 (0%) | 0 (0%) | 0 (0%) | 0 (0%) | 22 (20.6%) | 85 (79.4%) | 83 (77.6%) | 2 (1.9%) | 0 (0%) | 0 (0%) | 0 (0%) |
| Oliver et al., 2021[60] | Retrospective | | | Site | Trevo | | 86 | 36 (41.9%) | | 32 (37.2%) | | sICH not reported | | 69.6±18.3 | N/A | N/A | N/A | | 86 (100%) | 0 (0%) | 0 (0%) | 0 (0%) | 0 (0%) | 0 (0%) | 0 (0%) | 0 (0%) | 0 (0%) | 86 (100%) | 86 (100%) | 0 (0%) | 0 (0%) | 0 (0%) | 0 (0%) |
|  | |  |  | | |  | |  |  | |  | |  | | | |  |  |  |  |  |  |  |  |  |  |  |  |  |  |  |  |  |

* sICH – symptomatic Intracranial Hemorrhage definitions varied across studies. These were based on previously published definitions:

ECASS II: Any intracranial hemorrhage on any post-treatment image, within seven days of initiating treatment associated with a ≥ four-point deterioration on the NIHSS score from baseline or from the lowest score in seven days, or leading to death.

ECASS III: Any intracranial hemorrhage on follow-up imaging within seven days after stroke onset and an increase of ≥4 points on the NIHSS from baseline or the lowest value within 7 days, or mortality, with physician adjudicators finding a causal connection between the intracranial hemorrhage and neurological deterioration.

SITS-MOST: a local or remote type 2 parenchymal hemorrhage (PH2) on imaging 22–36 h after acute treatment with neurological deterioration of ≥4 points on the NIHSS from baseline or the lowest value between baseline and 24 h, or death within 24 h.

SWIFT: any parenchymal hematoma subarachnoid hemorrhage, or intraventricular hemorrhage associated with a worsening of the NIHSS score by four or more within 24 hours.

Heidelberg Bleeding Classification: new intracranial hemorrhage associated with any of the following: ≥4 point increase in the NIH Stroke Scale (compared to the immediate pre-deterioration status), ≥2 point increase in one NIH Stroke Scale subcategory, leading to major medical/surgical intervention such as intubation, hemicraniectomy, or external ventricular drain placement, in the absence of an alternative explanation for deterioration.

PROACT II: any type of hemorrhage on imaging studies and clinical deterioration of more than 4 points in the NIHSS score or a 1-point reduction in the level of consciousness with the NIHSS.

**Binning et al. only reported Core-lab adjudicated outcomes for 1,599 patients; in this meta-analysis, non-angiographic outcomes were reported for all patients, while angiographic outcomes were reported for 1,599 patients

## Supplementary Table 4. Combined (EmboTrap, Trevo, and Solitaire) ordinal mRS scores across treatment groups.

| **mRS Score** | **Combined Ordinal mRS** | **Percent (Combined Ordinal mRS)** | **EmboTrap** | **Trevo** | **Solitaire** |
| --- | --- | --- | --- | --- | --- |
| 0 | 957 | 16.0% | 100 | 497 | 360 |
| 1 | 1079 | 18.1% | 92 | 508 | 479 |
| 2 | 933 | 15.6% | 98 | 387 | 448 |
| 3 | 781 | 13.1% | 48 | 332 | 401 |
| 4 | 763 | 12.8% | 63 | 346 | 354 |
| 5 | 324 | 5.4% | 19 | 128 | 177 |
| 6 | 1128 | 18.9% | 55 | 400 | 673 |
| **Total Patient Population** | **5965** | **100%** | **475** | **2598** | **2892** |

Data are counts and percentages. mRS=modified Rankin Scale.

## Supplementary Results 1: Ordinal mRS scores at 90 days

## Among the 22 studies with sufficient data to compare ordinal mRS scores at 90 days, pooled median scores were lower for patients treated with EmboTrap (1.63, 95% CI 0.94-2.23) compared to Solitaire (2.73, 95% CI 2.30-3.17; p=0.018); the pooled median mRS score for patients treated with Trevo was 2.45 (95% CI 1.93-2.98) and did not differ significantly from the other devices (p=0.118 vs. EmboTrap, p=0.441 vs. Solitaire; Table 2). A table of combined ordinal mRS scores across treatment groups is shown in Supplementary Table 4.

## Supplementary Results 2: Core-lab subanalysis:

*Recanalization Outcomes*

Results from studies that used an independent corelab to adjudicate recanalization outcomes are presented in **Supplementary Table 5.**

Regarding FPR mTICI ≥2c, rates among core-lab adjudicated studies were 40.1% for EmboTrap, 27.6% for Trevo, and 25.8% for Solitaire (**Supplementary Figure 15**).

Rates of mFPR mTICI ≥2b among core-lab adjudicated studies were 51.5% for EmboTrap, 32.7% for Trevo, and 35.8% for Solitaire; no formal statistical analyses were performed to determine significance due to insufficient number of studies (**Supplementary Figure 16**).

When final recanalization outcomes in core-lab adjudicated studies were analyzed, rates of TICI 3 trended lower for Trevo (14.1%) and Solitaire (35.4%) compared to EmboTrap (52.0%; see **Supplementary Figure 17**), but a formal statistical comparison of treatment groups was not performed due to limited study data.

Among the 11 core-lab adjudicated studies with sufficient data, although not statistically significant, pooled rates of final mTICI ≥2b for EmboTrap trended higher in comparison to Solitaire (89.0%, 95% CI 78.5-94.7 vs. 80.0%, 95% CI 73.4-85.4; p=0.052) and Trevo (81.8%, 95% CI 79.9-83.5; p=0.224); the rate of final mTICI ≥2b did not differ significantly between Trevo and Solitaire (p=0.601; see **Supplementary Figure 18**).

## Supplementary Results 3: Prospective-only subanalysis:

Full results from prospective studies are presented in **Supplementary Table 6.**

**Functional Outcomes**

*Modified Rankin Scale (mRS) Score at 90 Days*

Among the 17 prospective studies with sufficient data, pooled rates of mRS 0-2 for EmboTrap (56.8%, 95% CI 44.7-68.1; p<0.001) and Trevo (55.1%, 95% CI 53.0-57.3; p=0.029) were significantly higher compared to Solitaire (43.4%, 95% CI 40.1-46.8) . No significant difference in mRS 0-2 rates was observed between EmboTrap and Trevo (p=0.398’ see **Supplementary Figure 24**).

Among the 10 prospective studies with sufficient data to compare ordinal mRS scores at 90 days, pooled median scores trended higher for patients treated with Solitaire (2.82, 95% CI 2.24-3.40) compared to EmboTrap (1.50, 95% CI 0.52-2.48; p=0.079) and Trevo 2.00 (95% CI 1.93-2.06; p=0.111), but differences between groups were not statistically significant.

*Mortality*

Among the 15 prospective studies with sufficient data, pooled rates of mortality at 90 days for EmboTrap (11.0%, 95% CI 7.5-15.9; p<0.001) and Trevo (13.6%, 95% CI 8.8-20.3; p<0.001) were significantly lower compared to Solitaire (21.9%, 95% CI 19.0-25.2) were significantly higher compared to both E. No significant difference in mortality at 90 days was found between EmboTrap and Trevo (p=0.215; see **Supplementary Figure 25**).

**Safety Outcomes**

*Embolization to New Territory (ENT) or Distal Emboli*

Among the 11 prospective only studies with sufficient data, pooled rates of ENT did not differ significantly between subgroups according to the omnibus test of subgroup differences (p=0.426; **Supplementary Figure 22**) and were 3.9% (95% CI 1.2-12.1%) for EmboTrap, 2.8% (95% CI 1.3-5.8%) for Trevo, and 5.4% (95% CI 2.8-10.0%) for Solitaire, with a smaller difference but similar direction to the complete-case analysis.

*Symptomatic Intracranial Hemorrhage (sICH)*

Among the 19 prospective only studies with sufficient data, pooled rates of sICH for EmboTrap (2.2%, 95% CI 0.7-7.2; p=0.044) and Trevo (1.7%, 95% CI 1.2-2.3; p=0.004) were significantly lower compared to Solitaire (7.3%, 95% CI 5.0-10.7) No significant difference in sICH rates was observed between EmboTrap and Trevo (p=0.481; see **Supplementary Figure 23**).

**Recanalization Outcomes**

Of note, all studies that evaluated FPR mTICI ≥2c were prospective studies, so a separate subgroup analysis was not necessary.

Rates of mFPR mTICI ≥2b among prospective studies were 51.5% for EmboTrap, 36.3% (95% CI 26.3-47.8) for Trevo, and 47.0% (95% CI 42.0-52.0) for Solitaire. No formal pairwise comparisons relative to the EmboTrap group were performed due to insufficient number of studies. Rates of mFPR mTICI ≥2b among prospective studies trended higher in the Solitaire group compared to the Trevo group but was not statistically significant (p=0.055; **Supplementary Figure 19**).

Evaluating the prospective studies only, the rates of TICI 3 were 52.0% for EmboTrap, 28.8% (95% CI 6.1-71.6) for Trevo, and 44.9% (95% CI 28.5-62.6) for Solitaire. No formal pairwise comparisons relative to the EmboTrap group were performed due to insufficient number of studies. The difference in rates of TICI 3 between the Trevo and Solitaire groups was not statistically significant (p=0.371; see **Supplementary Figure 20**).

Among the 13 prospective only studies with sufficient data, pooled rates of mTICI ≥2b did not differ significantly between subgroups according to the omnibus test of subgroup differences (p=0.849; **Supplementary Figure 21**) and were 88.1% (95% CI 80.5-93.0%) for EmboTrap, 86.4% (95% CI 77.7-92.1%) for Trevo, and 85.7% (95% CI 78.9-90.5%) for Solitaire.

## Supplementary Table 5. Comparison of recanalization outcomes between EmboTrap^®^, Trevo, and Solitaire among studies with outcomes adjudicated by a core-laboratory, with pooled random effects estimates.

| **Recanalization Outcome** | **Number of Studies Reporting Outcome** | **Number of Study Arms Reporting Outcome** | **EmboTrap**^®^***** | **Trevo*** | **Solitaire*** | **P value**  **(EmboTrap**^®^ **vs. Trevo)** | **P value**  **(EmboTrap**^®^ **vs. Solitaire)** | **P value**  **(Trevo vs. Solitaire)** | **I^2^ (EmboTrap**^®^ **+ Trevo)**  **(95% CI)**  **P value** | **I^2^ (EmboTrap**^®^ **+ Solitaire)**  **(95% CI)**  **P value** | **I^2^**  **(Trevo + Solitaire)**  **(95% CI)**  **P value** | **Overall I^2^**  **(95% CI)**  **P value** |
| --- | --- | --- | --- | --- | --- | --- | --- | --- | --- | --- | --- | --- |
| FPR mTICI ≥2c | 3 | 3 | 91/227  (40.1%)  [N/A]** | 397/1438  (27.6%)  [N/A]** | 16/62  (25.8%)  [N/A]** | N/A | N/A | N/A | 93.1%  (77.2 – 97.9)  <0.001 | 76.0%  (0.0 – 96.6)  0.041 | 0.0%  (N/A)  0.756 | 86.5%  (61.3 – 95.3)  <0.001 |
| mFPR mTICI ≥2b | 4 | 4 | 117/227  (51.5%)  [N/A]** | 35/107  (32.7%)  [N/A]** | 56/159  (35.8%)  [25.9 – 47.1] | N/A | N/A | N/A | 90.2%  (64.2 – 97.3)  0.001 | 82.9%  (47.7 – 94.4)  0.003 | 9.0%  (0.0 – 90.5)  0.333 | 82.2%  (54.1 – 93.1)  <0.001 |
| TICI 3 | 8 | 8 | 118/227  (52.0%)  [N/A]** | 225/1599  (14.1%)  [N/A]** | 162/435  (35.4%)  [19.4 – 55.5] | N/A | N/A | N/A | 99.4%  (98.8 – 99.6)  <0.001 | 92.1%  (86.3 – 95.5)  <0.001 | 96.8%  (95.1 – 97.9)  <0.001 | 97.6%  (96.5 – 98.3)  <0.001 |
| mTICI ≥2b | 11 | 11 | 380/428  (89.0%)  [78.5 – 94.7] | 1395/1706  (81.8%)  [79.9 – 83.5] | 538/686  (80.0%)  [73.4 – 85.4] | 0.224 | 0.052 | 0.601 | 81.4%  (51.5 – 92.9)  0.001 | 82.0%  (67.0 – 90.2)  <0.001 | 68.8%  (37.4 – 84.4)  0.001 | 77.8%  (60.6 – 87.5)  <0.001 |

* Dichotomous data for individual subgroups are expressed as n/N (%) [95% CI]; % calculated represents pooled random effects estimate, not fixed percentage).

**Since only one study has the reported outcome and the between-study variance component cannot be estimated, 95% CIs for pooled outcomes and pairwise comparisons between different interventions are not reported.

Ordinal data for individual subgroups are expressed as pooled median (95% CI) [N], along with raw frequency counts and percentages for each ordinal score. All pooled estimates for dichotomous data are derived from random-effects models using the DerSimonian-Laird procedure for estimation of between-study variance[15]; 95% confidence intervals of the pooled results were computed using the Jackson method.[16] Pooled medians and corresponding 95% CIs were derived via random effects models using methods described by McGrath et al.[17] P-values for each pairwise comparison are provided using separate meta-regression analyses, considering the intervention as a categorical moderator. P-values for the overall heterogeneity (i.e., statistical heterogeneity) among the included studies are obtained from Q-tests of heterogeneity. The estimated percentage of variability in effect size estimates that is due to heterogeneity rather than sampling error is given by I^2^ statistics and their corresponding 95% CIs.[18] I^2^ values are given for each subgroup comparison and for the overall study population.

Abbreviations: CI=confidence interval, FPR=first pass recanalization, mFPR = modified first pass recanalization, mTICI=modified Thrombolysis in Cerebral Infarction, TICI=thrombolysis in cerebral infarction.

## Supplementary Table 6. Comparisons of functional, safety, and recanalization outcomes between EmboTrap®, Trevo, and Solitaire among prospective studies, with pooled random effects estimates.

| **Outcome** | **Number of Studies Reporting Outcome** | | **Number of Study Arms Reporting Outcome** | **EmboTrap**^®^***** | **Trevo*** | **Solitaire*** | **P value**  **(EmboTrap**^®^ **vs. Trevo)** | **P value**  **(EmboTrap**^®^ **vs. Solitaire)** | **P value**  **(Trevo vs. Solitaire)** | **I^2^ (EmboTrap**^®^ **+ Trevo)**  **(95% CI)**  **P value** | **I^2^ (EmboTrap**^®^ **+ Solitaire)**  **(95% CI)**  **P value** | **I^2^**  **(Trevo + Solitaire)**  **(95% CI)**  **P value** | **Overall I^2^**  **(95% CI)**  **P value** |
| --- | --- | --- | --- | --- | --- | --- | --- | --- | --- | --- | --- | --- | --- |
| mRS 0-2 at 90 days | 17 | | 17 | 272/456  (56.8%)  [44.7 – 68.1] | 1141/2070  (55.1%)  [53.0 – 57.3] | 674/1576  (43.4%)  [40.1 – 46.8] | 0.398 | <0.001 | 0.029 | 67.2%  (27.0 – 85.3)  0.006 | 80.1%  (67.5 – 87.9)  <0.001 | 82.4%  (71.1 – 89.2)  <0.001 | 83.5%  (74.8 – 89.2)  <0.001 |
| mRS at 90 days | 10 | | 10 | 1.50  (0.52 – 2.48)  [397] | 2.00  (1.93 – 2.06)  [1920] | 2.82  (2.24 – 3.40)  [395] | 0.250 | 0.079 | 0.111 | 97.0%  (89.2 – 99.8) | 96.7%  (92.1 – 99.2) | 93.9%  (87.7 – 98.7) | 97.6%  (94.7 – 99.3) |
| 0 | . | | . | 72 (18.1%) | 414 (21.6%) | 58 (14.7%) | . | . | . | . | . | . | . |
| 1 | . | | . | 82 (20.7%) | 370 (19.3%) | 66 (16.7%) | . | . | . | . | . | . | . |
| 2 | . | | . | 87 (21.9%) | 275 (14.3%) | 56 (14.2%) | . | . | . | . | . | . | . |
| 3 | . | | . | 41 (10.3%) | 248 (12.9%) | 58 (14.7%) | . | . | . | . | . | . | . |
| 4 | . | | . | 54 (13.6%) | 252 (13.1%) | 40 (10.1%) | . | . | . | . | . | . | . |
| 5 | . | | . | 15 (3.8%) | 87 (4.5%) | 37 (9.4%) | . | . | . | . | . | . | . |
| 6 | . | | . | 46 (11.6%) | 274 (14.3%) | 80 (20.3%) | . | . | . | . | . | . | . |
| Mortality at 90 days | | 15 | 15 | 51/481  (11.0%)  [7.5 – 15.9] | 290/2093  (13.6%)  [8.8 – 20.3] | 152/702  (21.9%)  [19.0 – 25.2] | 0.215 | <0.001 | <0.001 | 44.2%  (0.0 – 76.5)  0.097 | 65.2%  (35.7 – 81.2)  <0.001 | 70.1%  (44.4 – 83.9)  <0.001 | 69.4%  (47.9 – 82.0)  <0.001 |
| ENT or distal emboli | | 11 | 11 | 19/428  (3.9%)  [1.2 – 12.1] | 48/2032  (2.8%)  [1.3 – 5.8] | 57/775  (5.4%)  [2.8 – 10.0] | 0.771 | 0.628 | 0.410 | 79.2%  (44.5 – 92.2)  0.002 | 75.1%  (52.0 – 87.1)  <0.001 | 88.7%  (80.8 – 93.4)  <0.001 | 86.8%  (78.3 – 92.0)  <0.001 |
| sICH | | 19 | 19 | 14/508  (2.2%)  [0.7 – 7.2] | 35/2093  (1.7%)  [1.2 – 2.3] | 183/1828  (7.3%)  [5.0 – 10.7] | 0.481 | 0.044 | 0.004 | 58.1%  (3.2 – 81.9)  0.026 | 77.9%  (64.6 – 86.2)  <0.001 | 91.4%  (87.5 – 94.1)  <0.001 | 89.8%  (85.6 – 92.8)  <0.001 |
| FPR mTICI ≥2c | | 5 | 6 | 91/227  (40.1%)  [N/A]** | 406/1493  (23.1%)  [13.9 – 36.0] | 123/381  (32.4%)  [27.9 – 37.3] | N/A | N/A | 0.220 | 89.3%  (71.0 – 96.1)  <0.001 | 46.3%  (0.0 – 82.1)  0.134 | 55.9%  (0.0 – 83.7)  0.060 | 77.5%  (50.0 – 89.9)  <0.001 |
| mFPR mTICI ≥2b | | 5 | 6 | 117/227  (51.5%)  [N/A]** | 38/105  (36.3%)  [26.3 – 47.8] | 179/381  (47.0%)  [42.0 – 52.0] | N/A | N/A | 0.055 | 74.6%  (15.6 – 92.4)  0.020 | 0.0%  (0.0 – 84.7)  0.528 | 34.5%  (0.0 – 75.3)  0.191 | 43.9%  (0.0 – 77.8)  0.112 |
| TICI 3 | | 9 | 10 | 118/227  (52.0%)  [N/A]** | 243/1634  (28.8%)  [6.1 – 71.6] | 261/596  (44.9%)  [28.5 – 62.6] | N/A | N/A | 0.371 | 98.8%  (98.1 – 99.3)  <0.001 | 91.8%  (86.2 – 95.1)  <0.001 | 97.1%  (95.9 – 98.0)  <0.001 | 97.4%  (96.5 – 98.1)  <0.001 |
| mTICI ≥2b | | 13 | 14 | 414/468  (88.1%)  [80.5 – 93.0] | 1400/1704  (86.4%)  [77.7 – 92.1] | 1227/1459  (85.7%)  [78.9 – 90.5] | 0.838 | 0.593 | 0.782 | 75.3%  (44.1 – 89.1)  0.001 | 84.3%  (73.4 – 90.7)  <0.001 | 82.0%  (68.9 – 89.5)  <0.001 | 81.6%  (70.1 – 88.6)  <0.001 |

* Dichotomous data for individual subgroups are expressed as n/N (%) [95% CI]; % calculated represents pooled random effects estimate, not fixed percentage).

**Since only one study has the reported outcome and the between-study variance component cannot be estimated, 95% CIs for pooled outcomes and pairwise comparisons between different interventions are not reported.

Ordinal data for individual subgroups are expressed as pooled median (95% CI) [N], along with raw frequency counts and percentages for each ordinal score. All pooled estimates for dichotomous data are derived from random-effects models using the DerSimonian-Laird procedure for estimation of between-study variance[15]; 95% confidence intervals of the pooled results were computed using the Jackson method.[16] Pooled medians and corresponding 95% CIs were derived via random effects models using methods described by McGrath et al.[17] P-values for each pairwise comparison are provided using separate meta-regression analyses, considering the intervention as a categorical moderator. P-values for the overall heterogeneity (i.e., statistical heterogeneity) among the included studies are obtained from Q-tests of heterogeneity. The estimated percentage of variability in effect size estimates that is due to heterogeneity rather than sampling error is given by I^2^ statistics and their corresponding 95% CIs.[18] I^2^ values are given for each subgroup comparison and for the overall study population.

Abbreviations: CI=confidence interval, ENT=embolization to new territory, FPR=first pass recanalization, mFPR = modified first pass recanalization, mRS=modified Rankin Scale, mTICI=modified Thrombolysis in Cerebral Infarction, sICH=symptomatic intracerebral hemorrhage, TICI=thrombolysis in cerebral infarction.

## Supplementary Figure 1. Outlier and influence analyses of rates of mRS 0-2 at 90 days after performing 1 million randomly selected iterations from 2^k^-1 possible study combinations. (A-G) GOSH plots were multimodal with a wide range of pooled effect estimates and statistical heterogeneity across possible meta-analysis combinations. The clustering solution and the amount of cluster imbalance pertaining to each study in each cluster are shown using a (A) Gaussian mixture model (GMM) algorithm, (B) k-means algorithm, and (C) density based spatial clustering of applications with noise (DBSCAN) algorithm. The delta percentage indicates the degree of cluster imbalance contributed by a specific study, using the difference between 1) the expected proportion of subsets containing a specific study, given that the cluster composition is purely random, and 2) the actual proportion of subsets containing a specific study within a given cluster. A corresponding Cook’s distance is obtained from a linear intercept model, with a Cook’s distance three times above the mean across the generated clusters indicating an influential case and/or outlier. (D-G) The distribution of effect sizes and statistical heterogeneity across meta-analyses that include or exclude specific outlier/influential cases identified from the 3 clustering algorithms, with combinations including outliers/influential cases shown in cyan. Combinations are shown after excluding (D) Binning et al., 2018[26], (E) He et al., 2022[47], (F) Slezak et al., 2017[38], and (G) Zaidat et al., 2018[7].


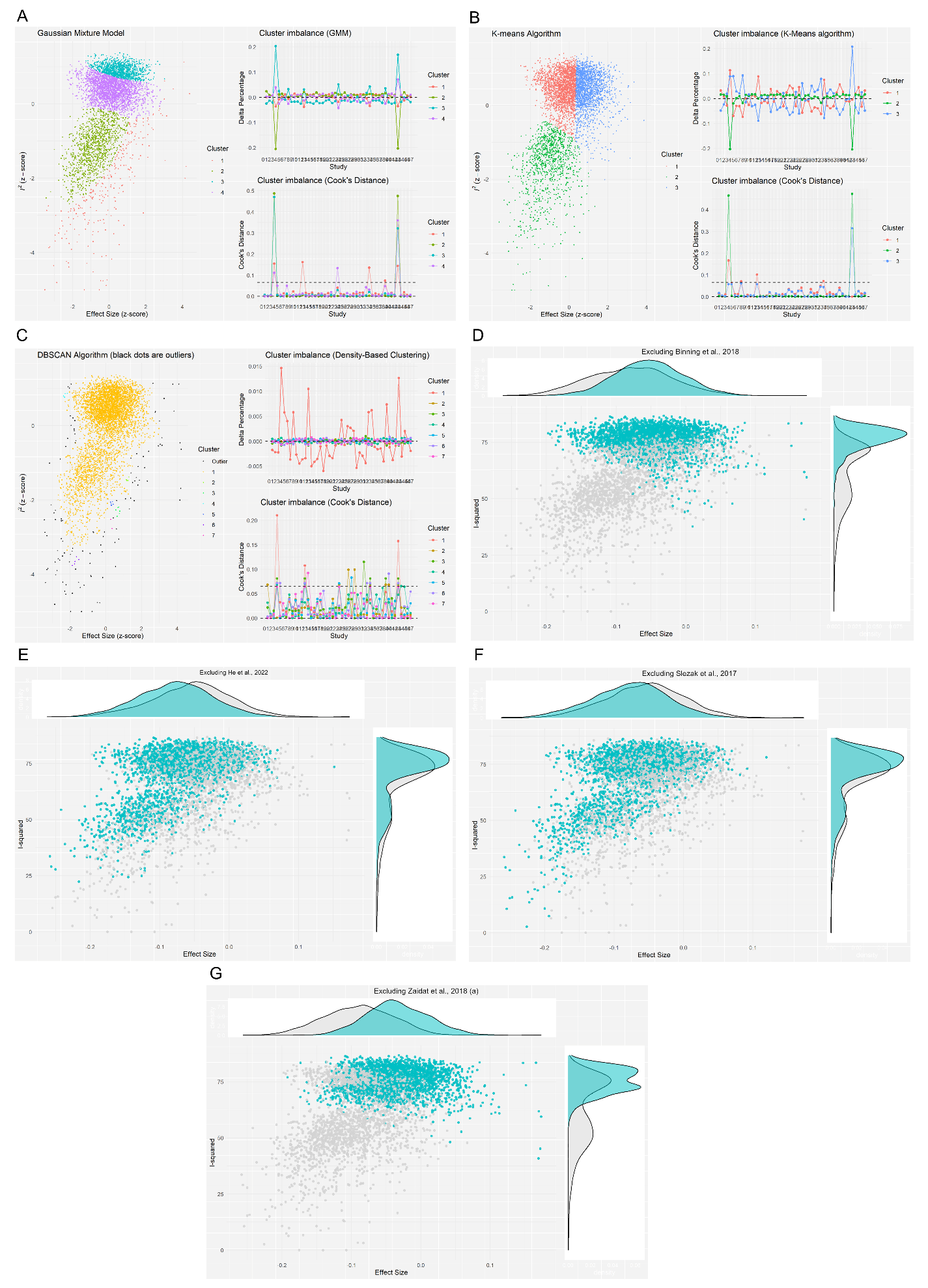


## Supplementary Figure 2. Forest plot of comparisons of mRS 0-2 at 90 days, showing the impact of outliers on the complete-case analysis. (A) Comparisons of mRS 0-2 at 90 days from the complete-case analysis, with outlier studies shown in pink. (B) Comparisons of mRS 0-2 at 90 days after removing outlier studies.
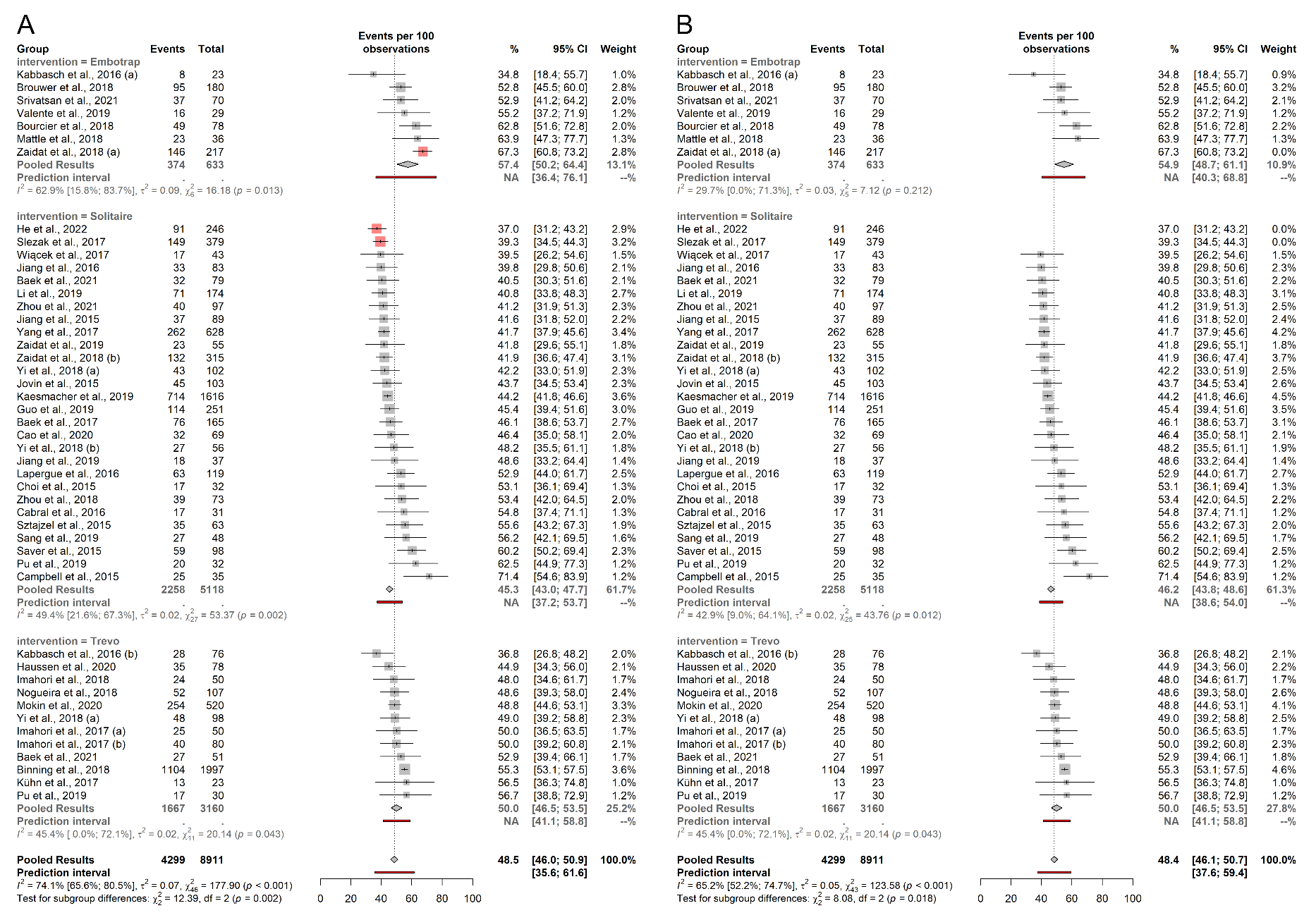


## Supplementary Figure 3. Forest plot of comparisons of mRS 0-2 at 90 days, showing the impact of non-outlier, influential studies on the complete-case analysis. (A) Comparisons of mRS 0-2 at 90 days from the complete-case analysis, and non-outlier, influential studies shown in blue. (B) Comparisons of mRS 0-2 at 90 days after removing influential studies.


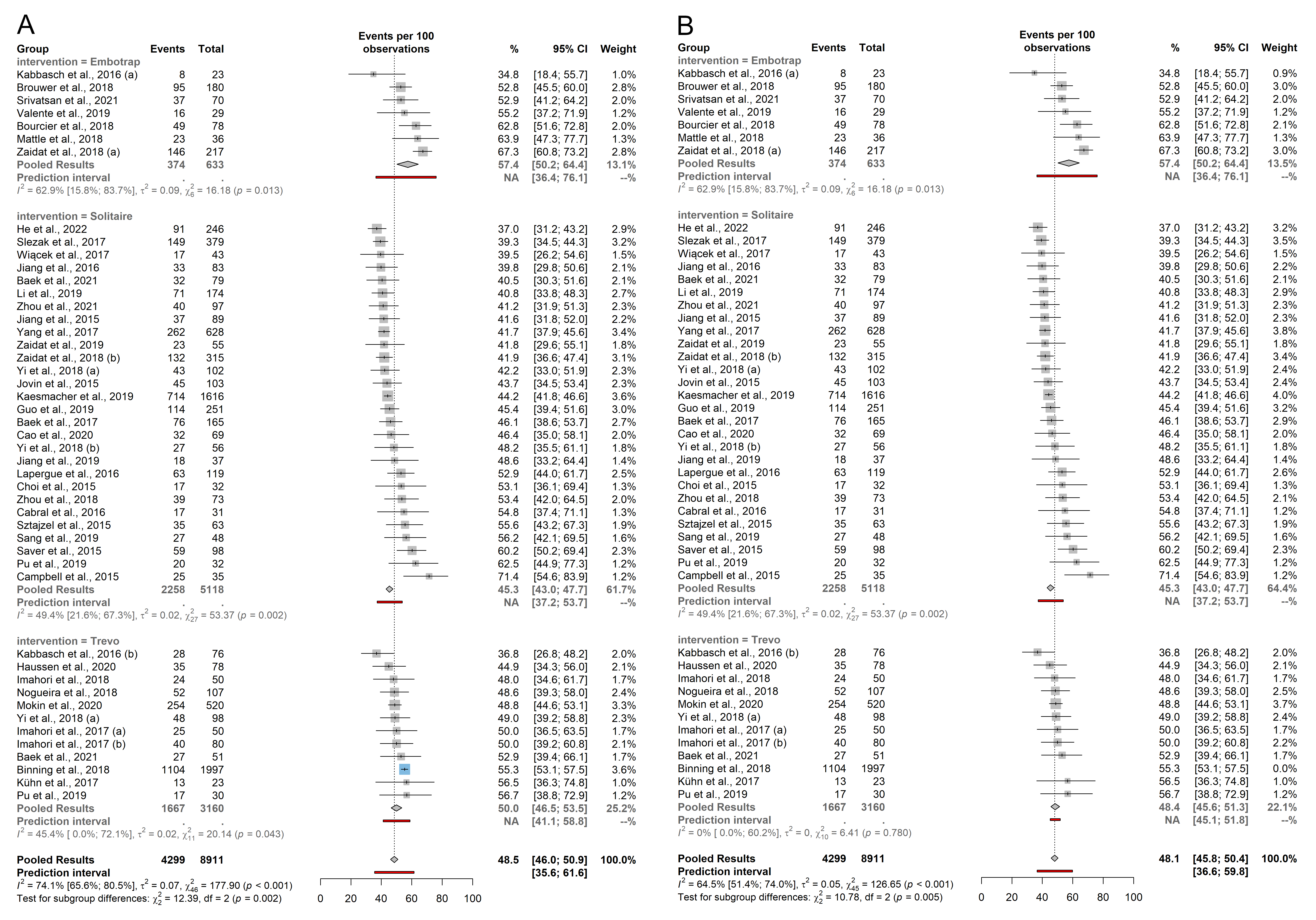


## Supplementary Figure 4. Outlier and influence analyses of rates of mortality at 90 days after performing 1 million randomly selected iterations from 2^k^-1 possible study combinations. (A-J) GOSH plots were multimodal with a wide range of pooled effect estimates and statistical heterogeneity across possible meta-analysis combinations. The clustering solution and the amount of cluster imbalance pertaining to each study in each cluster are shown using a (A) Gaussian mixture model (GMM) algorithm, (B) k-means algorithm, and (C) density based spatial clustering of applications with noise (DBSCAN) algorithm. The delta percentage indicates the degree of cluster imbalance contributed by a specific study, using the difference between 1) the expected proportion of subsets containing a specific study, given that the cluster composition is purely random, and 2) the actual proportion of subsets containing a specific study within a given cluster. A corresponding Cook’s distance is obtained from a linear intercept model, with a Cook’s distance three times above the mean across the generated clusters indicating an influential case and/or outlier. (D-J) The distribution of effect sizes and statistical heterogeneity across meta-analyses that include or exclude specific outlier/influential cases identified from the 3 clustering algorithms, with combinations including outliers/influential cases shown in cyan. Combinations are shown after excluding (D) Binning et al., 2018[26], (E) Kaesmacher et al., 2019[28], (F) Li et al., 2019[56], (G) Slezak et al., 2017[38], (H) Yi et al., 2018[67], (I) Zaidat et al., 2018a[7], and (J) Zaidat et al., 2018b.[32]


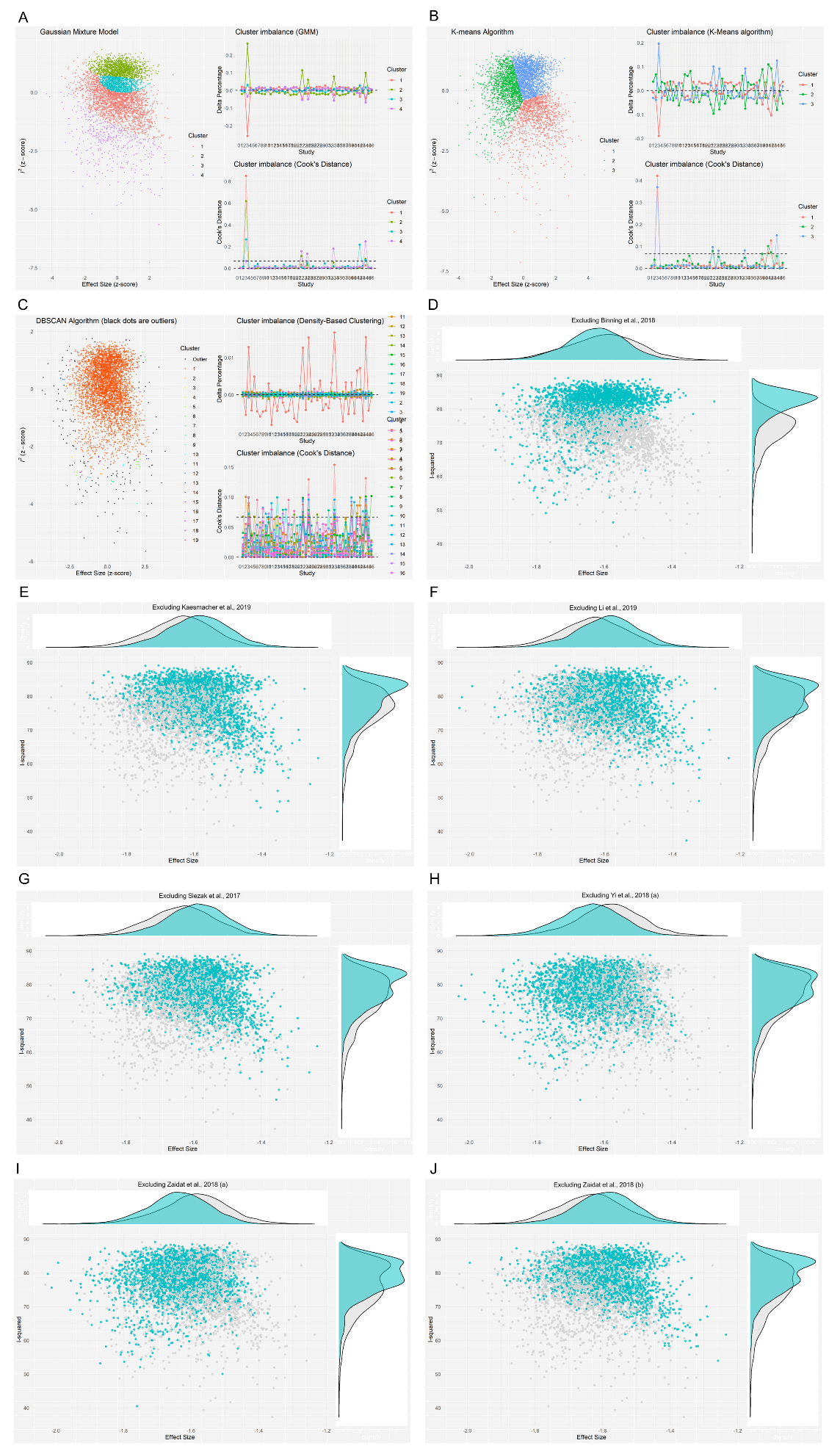


## Supplementary Figure 5. Forest plot of comparisons of mortality at 90 days, showing the impact of outliers on the complete-case analysis. (A) Comparisons of mortality at 90 days from the complete-case analysis, with outlier studies shown in pink. (B) Comparisons of mortality at 90 days after removing outlier studies.


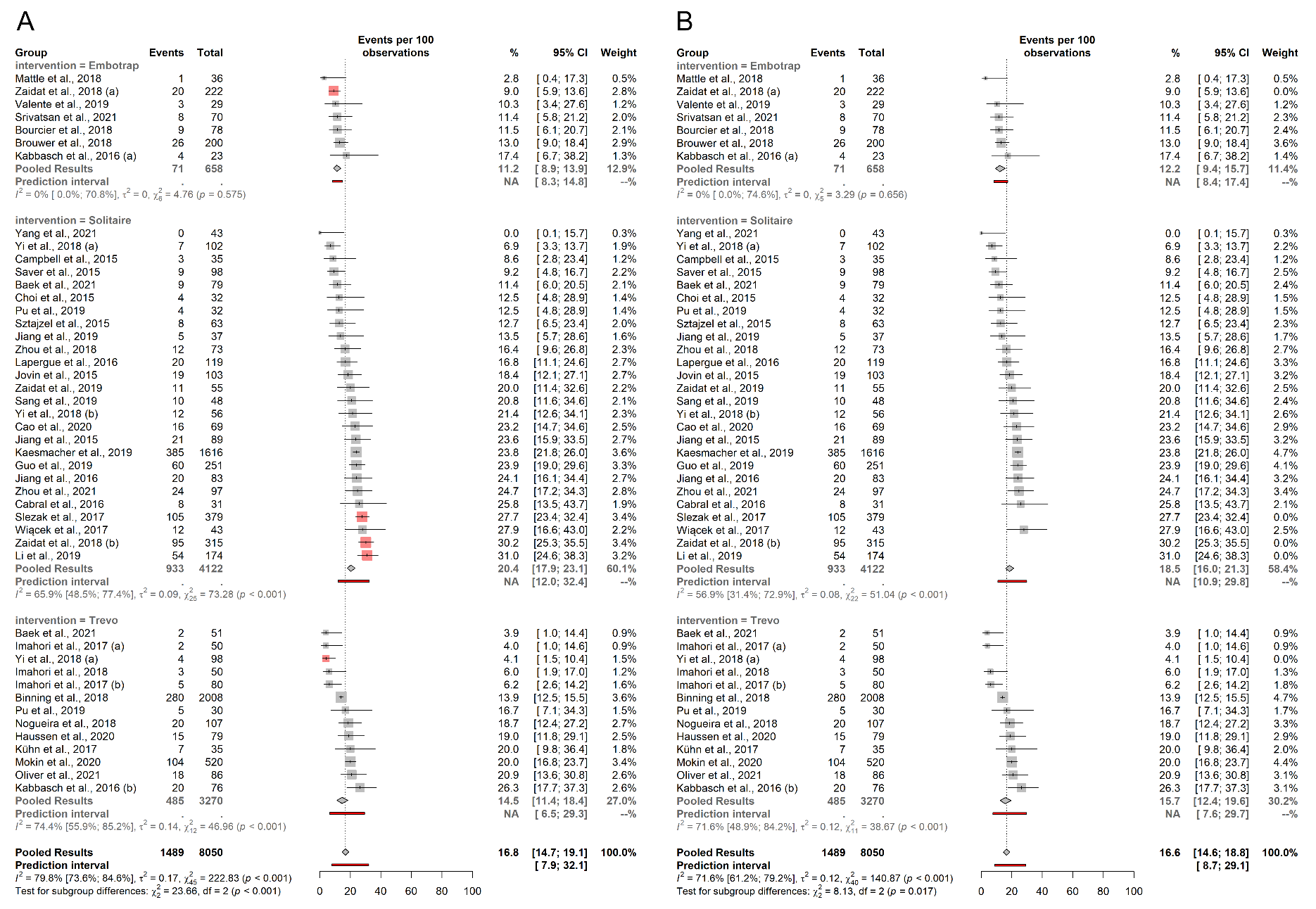


## Supplementary Figure 6. Forest plot of comparisons of mortality at 90 days, showing the impact of non-outlier, influential studies on the complete-case analysis. (A) Comparisons of mortality at 90 days from the complete-case analysis, and non-outlier, influential studies shown in blue. (B) Comparisons of mortality at 90 days after removing influential studies.


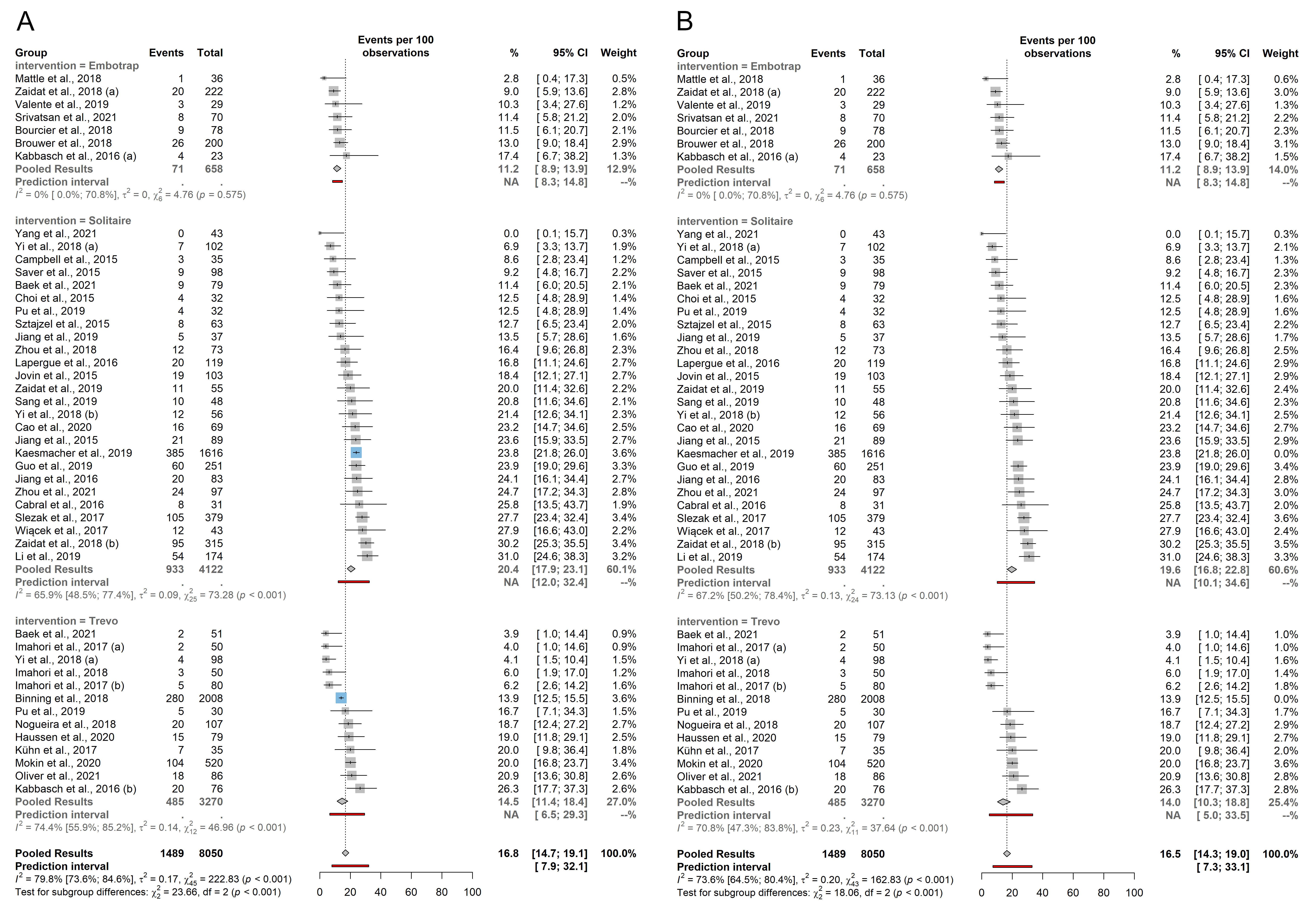


## Supplementary Figure 7. Outlier and influence analyses of ENT rates after performing 1 million randomly selected iterations from 2^k^-1 possible study combinations. (A-F) GOSH plots were multimodal with a wide range of pooled effect estimates and statistical heterogeneity across possible meta-analysis combinations. The clustering solution and the amount of cluster imbalance pertaining to each study in each cluster are shown using a (A) Gaussian mixture model (GMM) algorithm, (B) k-means algorithm, and (C) density based spatial clustering of applications with noise (DBSCAN) algorithm. The delta percentage indicates the degree of cluster imbalance contributed by a specific study, using the difference between 1) the expected proportion of subsets containing a specific study, given that the cluster composition is purely random, and 2) the actual proportion of subsets containing a specific study within a given cluster. A corresponding Cook’s distance is obtained from a linear intercept model, with a Cook’s distance three times above the mean across the generated clusters indicating an influential case and/or outlier. (D-F) The distribution of effect sizes and statistical heterogeneity across meta-analyses that include or exclude specific outlier/influential cases identified from the 3 clustering algorithms, with combinations including outliers/influential cases shown in cyan. Combinations are shown after (D) excluding Binning et al., 2018,[26] (E) excluding Kaesmacher et al., 2019,[28] and (F) excluding Srivatsan et al., 2021.[30]


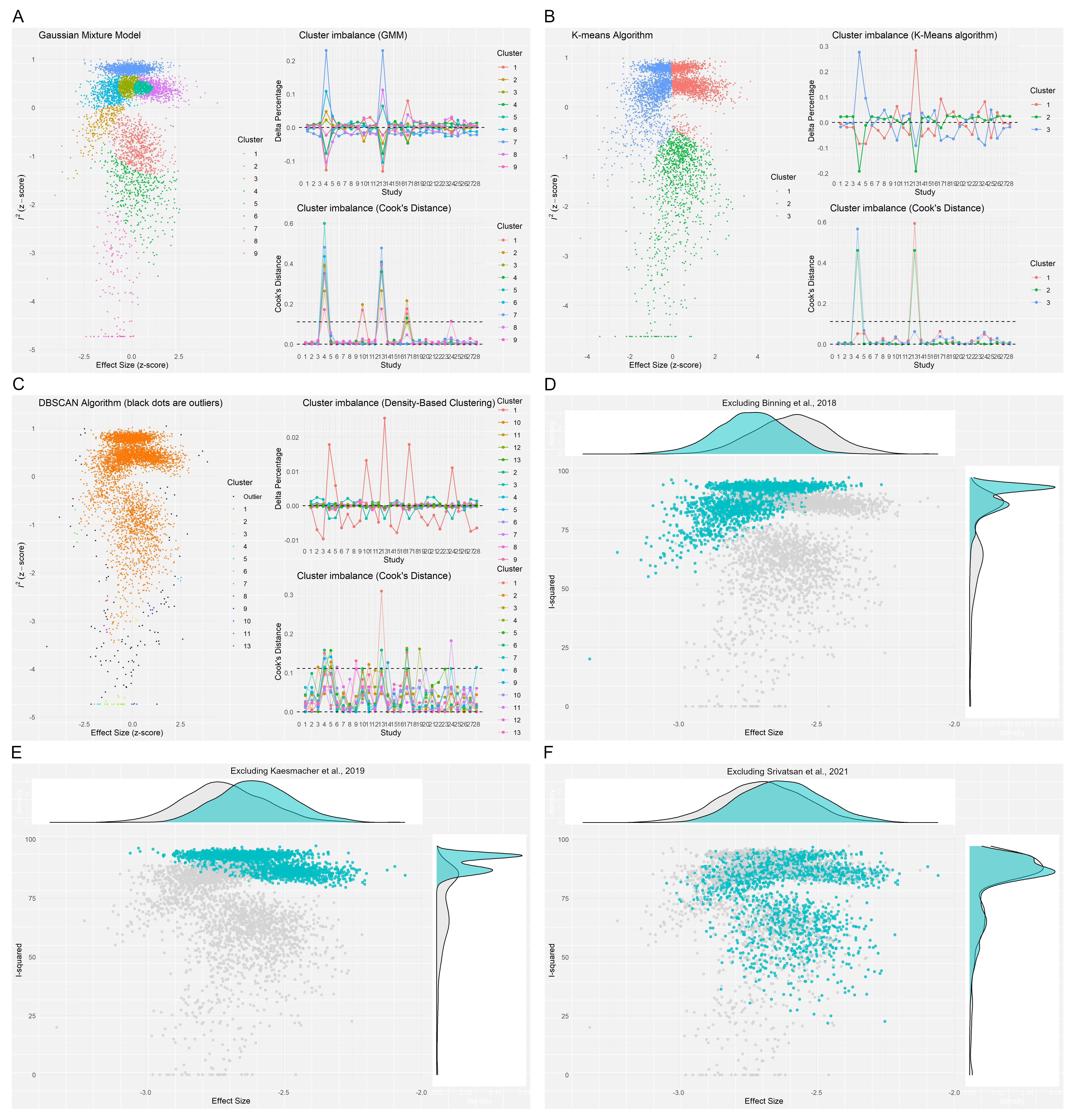


## Supplementary Figure 8. Forest plot of comparisons of ENT/distal emboli. (A) Comparisons of ENT/distal emboli from the complete-case analysis, with outlier studies shown in pink. (B) Comparisons of ENT/distal emboli after removing outlier studies.

**
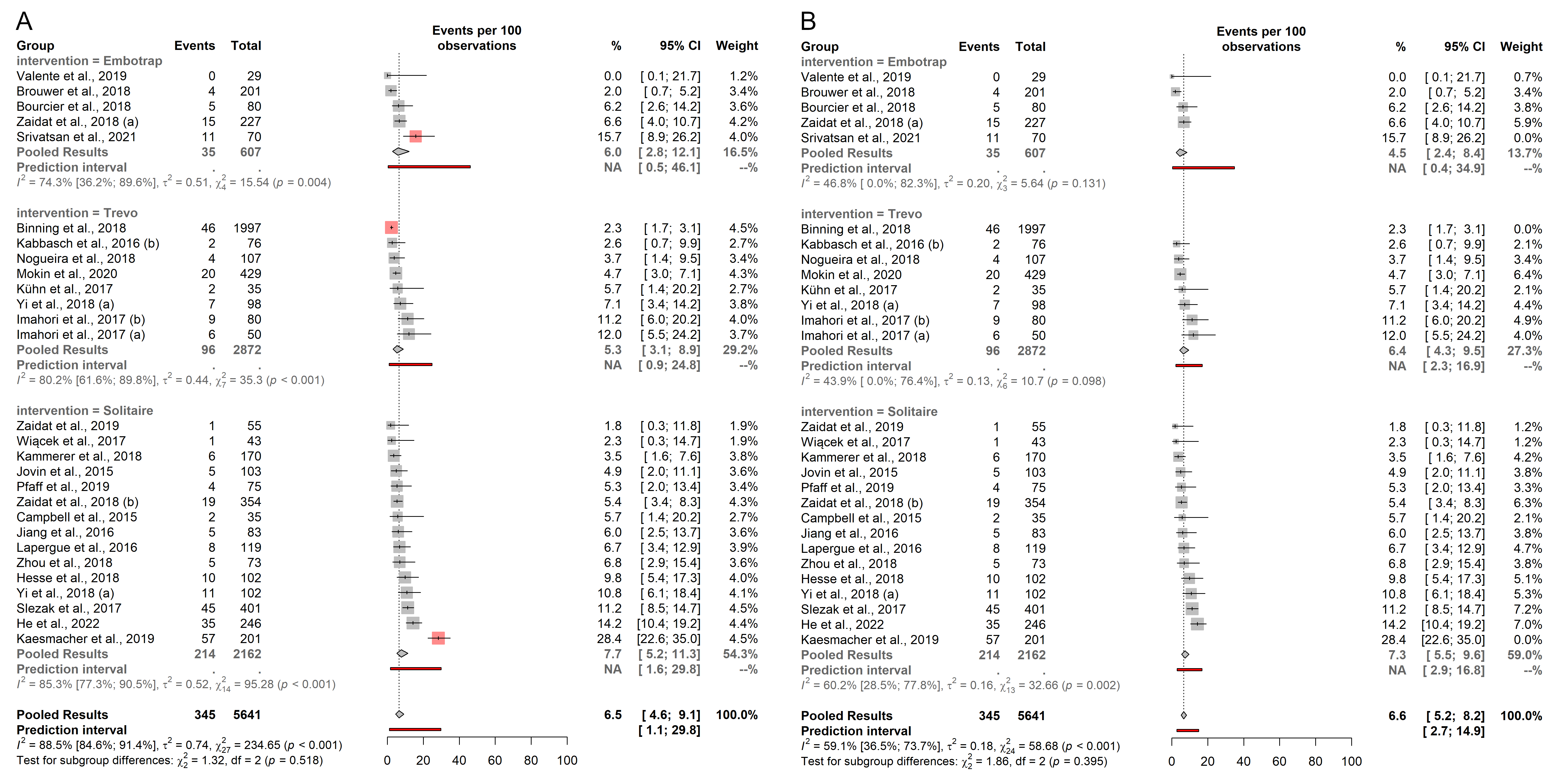
**

## Supplementary Figure 9. Outlier and influence analyses of sICH rates after performing 1 million randomly selected iterations from 2^k^-1 possible study combinations. (A-E) GOSH plots were multimodal with a wide range of pooled effect estimates and statistical heterogeneity across possible meta-analysis combinations. The clustering solution and the amount of cluster imbalance pertaining to each study in each cluster are shown using a (A) Gaussian mixture model (GMM) algorithm, (B) k-means algorithm, and (C) density based spatial clustering of applications with noise (DBSCAN) algorithm. The delta percentage indicates the degree of cluster imbalance contributed by a specific study, using the difference between 1) the expected proportion of subsets containing a specific study, given that the cluster composition is purely random, and 2) the actual proportion of subsets containing a specific study within a given cluster. A corresponding Cook’s distance is obtained from a linear intercept model, with a Cook’s distance three times above the mean across the generated clusters indicating an influential case and/or outlier. (D-E) The distribution of effect sizes and statistical heterogeneity across meta-analyses that include or exclude specific outlier/influential cases identified from the 3 clustering algorithms, with combinations including outliers/influential cases shown in cyan. Combinations are shown after (D) excluding Yang et al., 2018,[66] and (E) excluding Binning et al., 2018.[26]


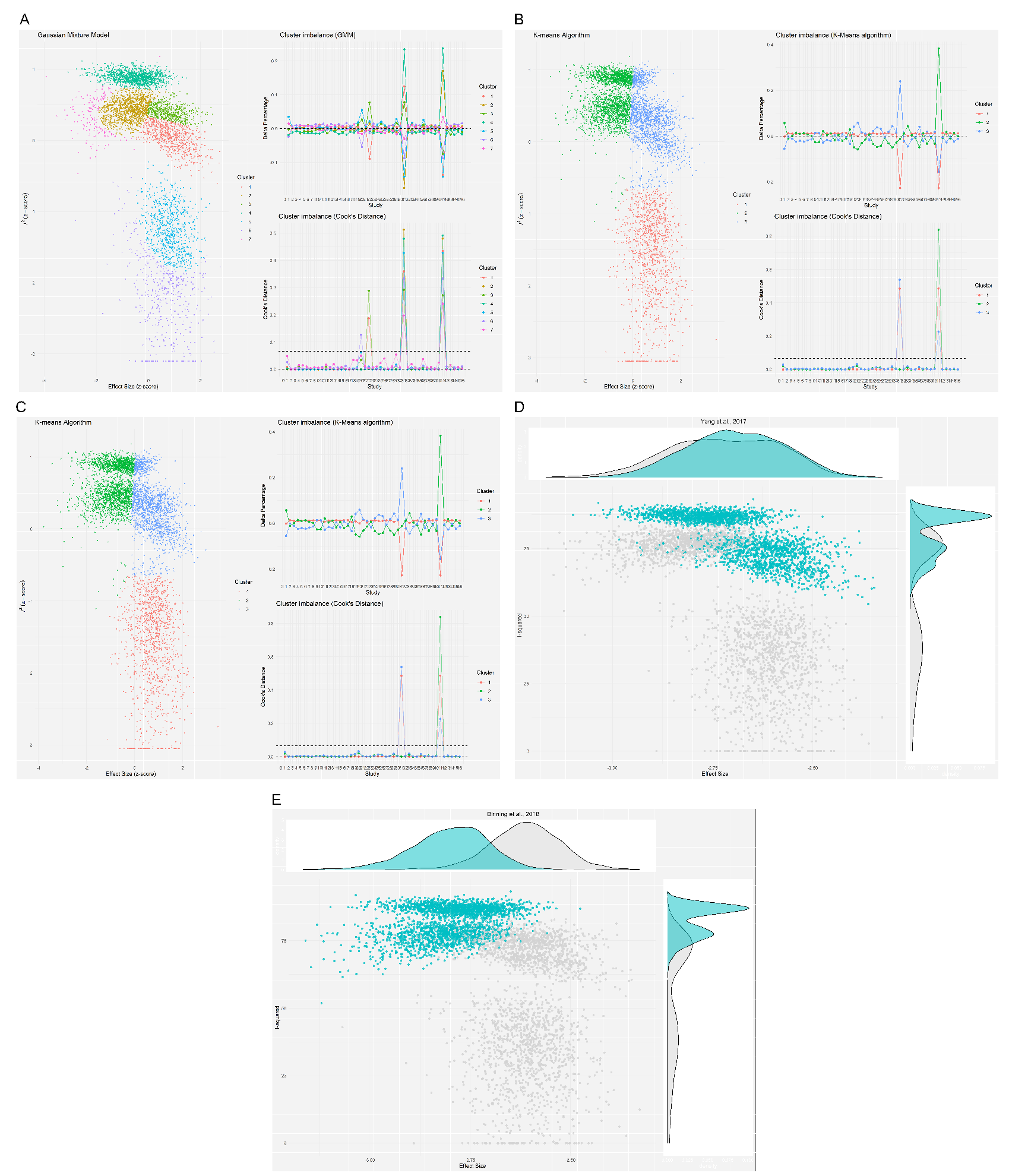


## Supplementary Figure 10. Forest plot of comparisons of sICH. (A) Comparisons of sICH from the complete-case analysis, with outlier studies shown in pink. (B) Comparisons of sICH after removing outlier studies.


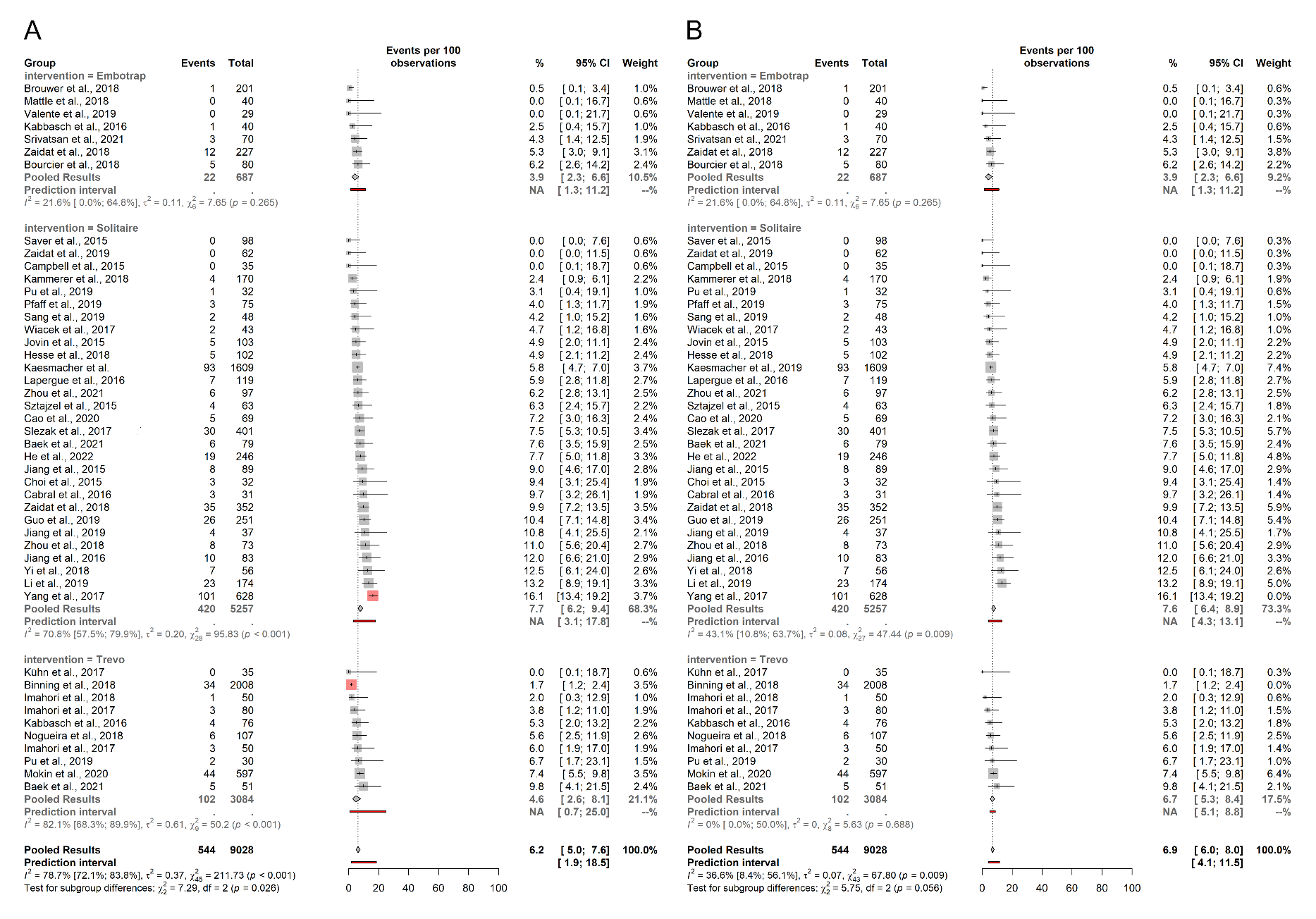


## Supplementary Figure 11. Forest plot of comparisons of complete or near-complete recanalization on first pass (FPR mTICI ≥2c).

**

**

## Supplementary Figure 12. Forest plot of comparisons of successful recanalization on first pass (mFPR mTICI ≥2b).

**

**

## Supplementary Figure 13. Forest plot of comparisons of final complete recanalization (TICI 3).

**

**

## Supplementary Figure 14. Forest plot of comparisons of final successful recanalization (mTICI ≥2b).

**

**

## Supplementary Figure 15. Forest plot of comparisons of complete or near-complete recanalization on first pass (FPR mTICI ≥2c) among core-lab adjudicated studies.



## Supplementary Figure 16. Forest plot of comparisons of successful recanalization on first pass (mFPR mTICI ≥2b) among core-lab adjudicated studies.

**

**

## Supplementary Figure 17. Forest plot of comparisons of final complete recanalization (TICI 3) among core-lab adjudicated studies.

*
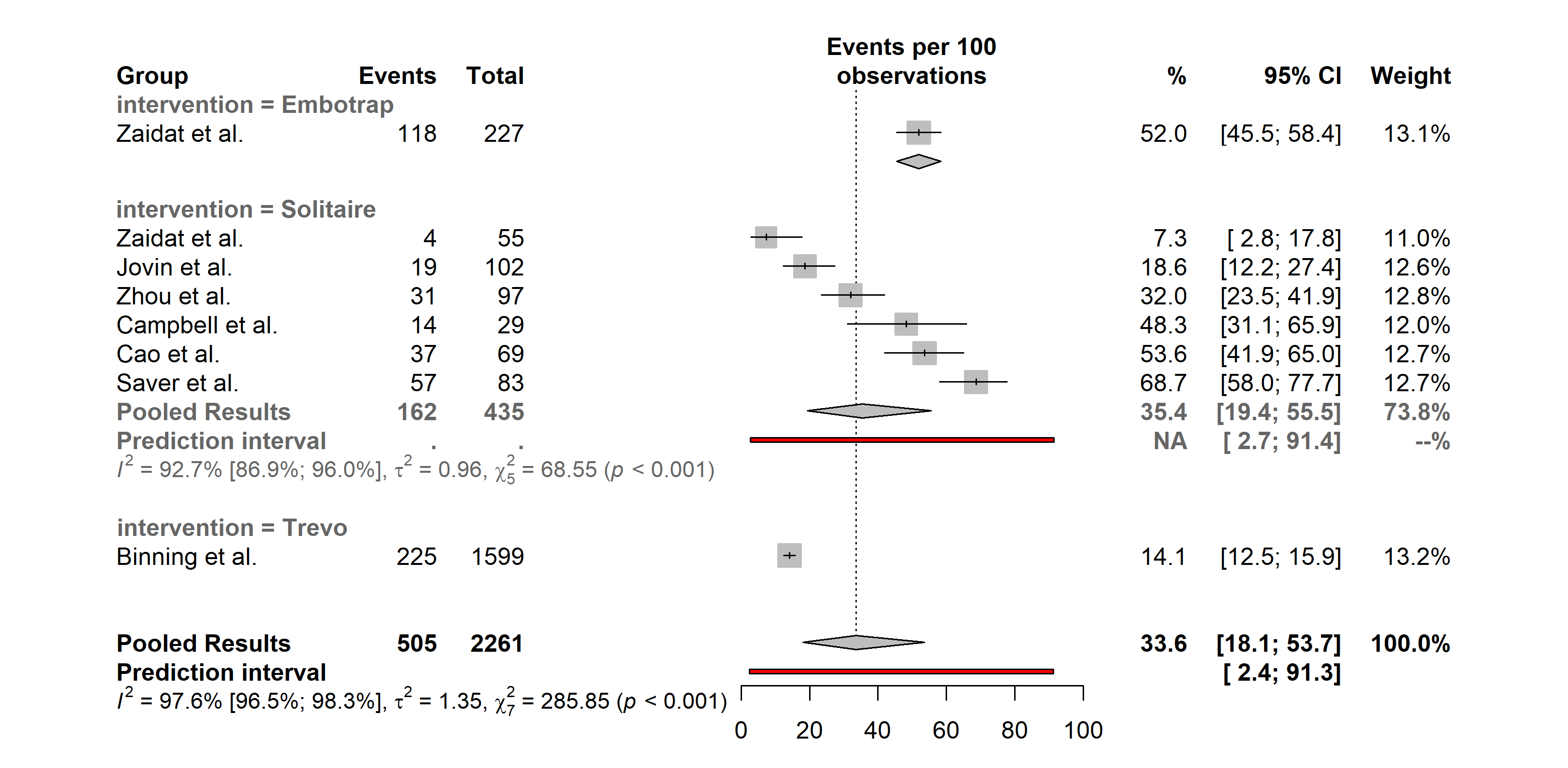
*

## Supplementary Figure 18. Forest plot of comparisons of final successful recanalization (mTICI ≥2b) among core-lab adjudicated studies
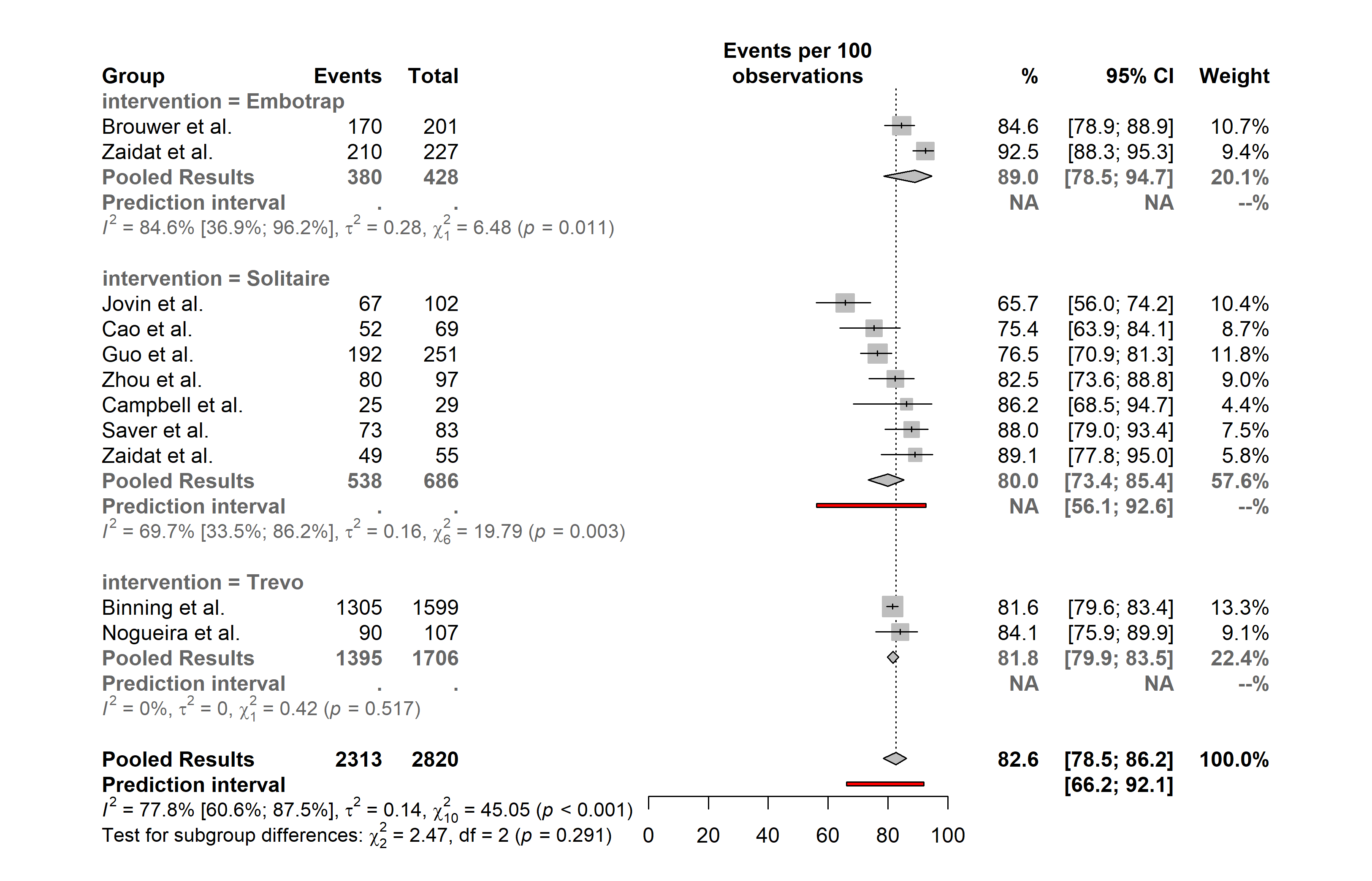


## Supplementary Figure 19. Forest plot of comparisons of successful recanalization on first pass (mFPR mTICI ≥2b) among prospective studies.

**
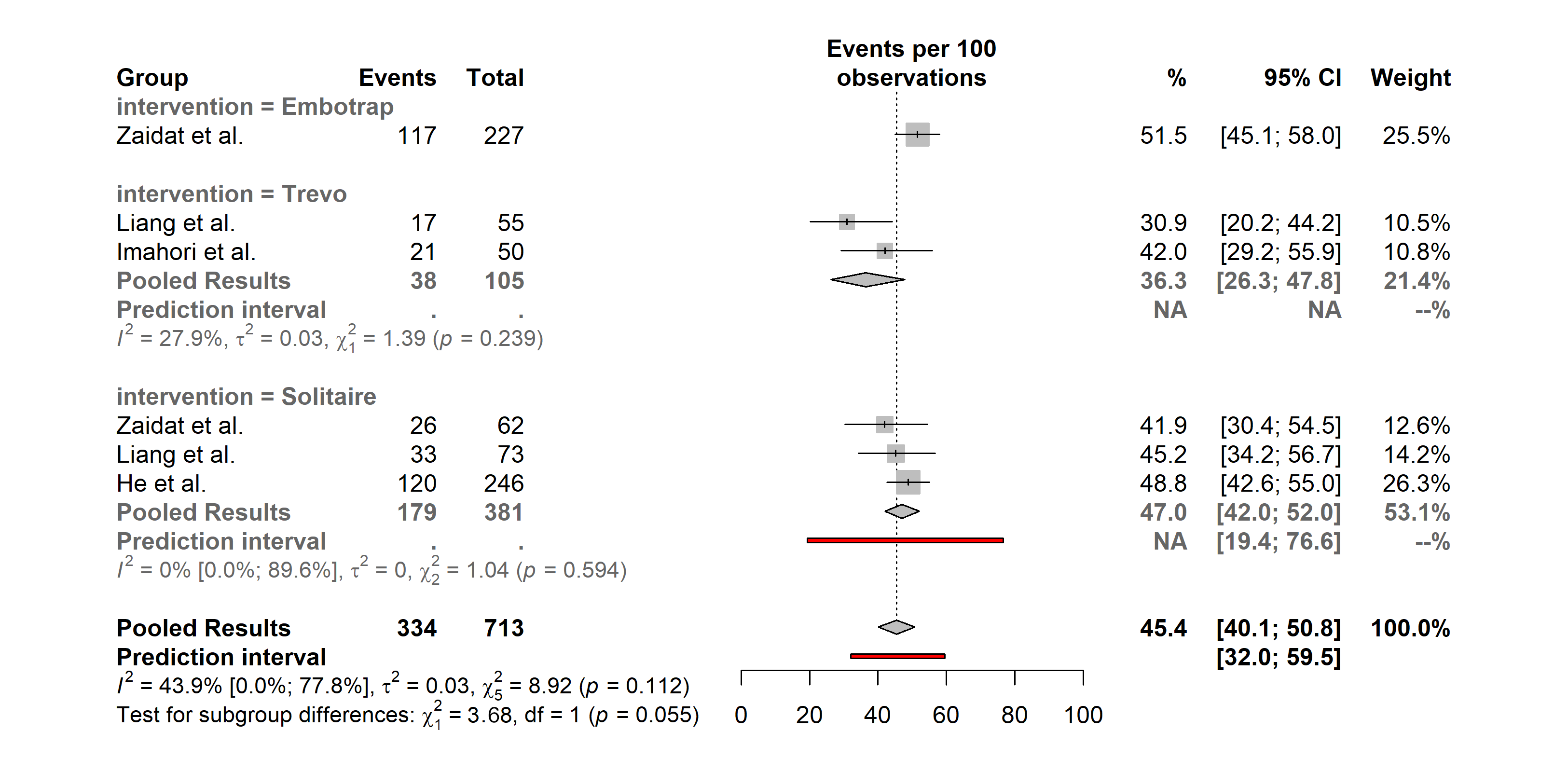
**

## Supplementary Figure 20. Forest plot of comparisons of final complete recanalization (TICI 3) among prospective studies.

*
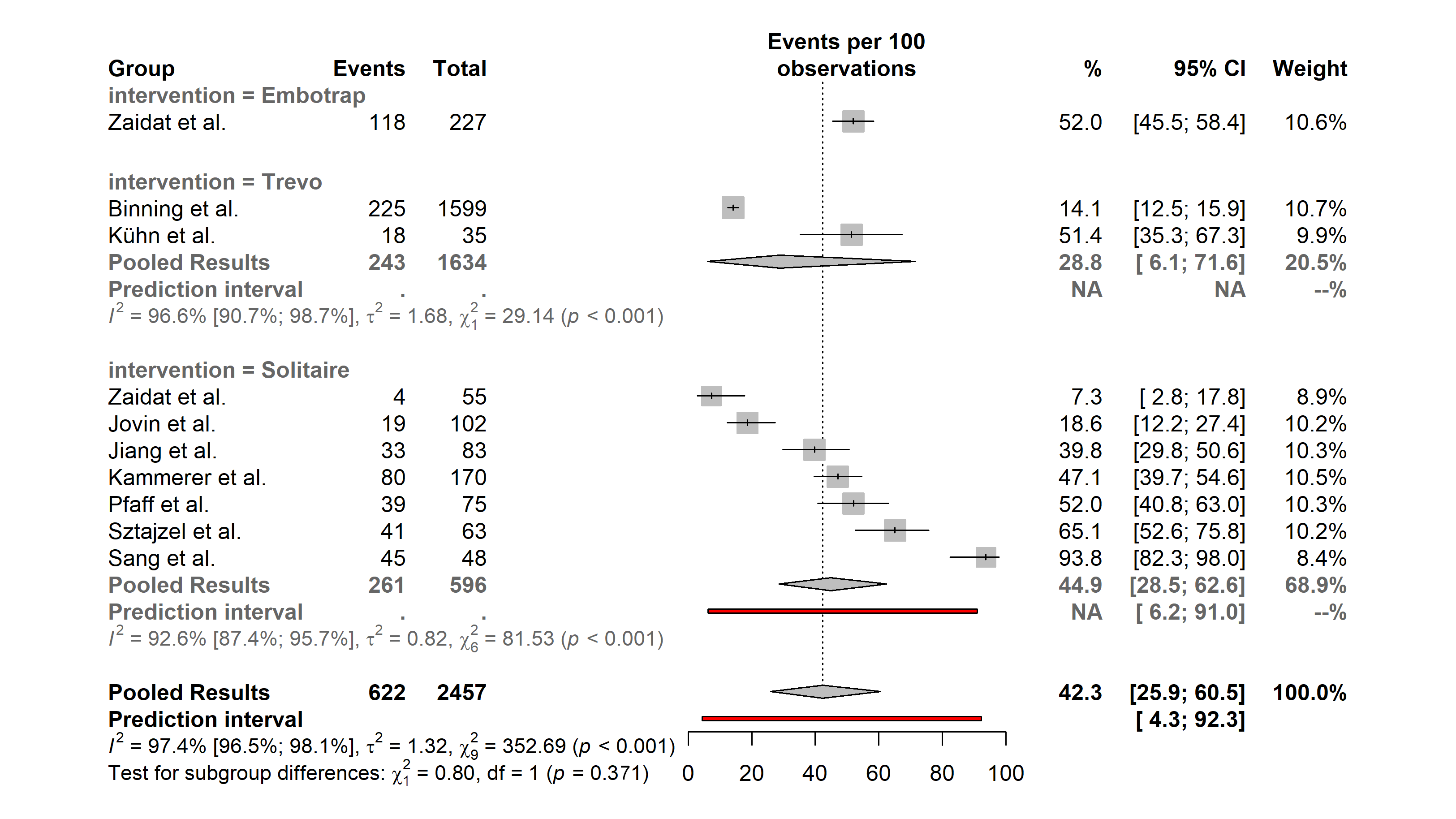
*

## Supplementary Figure 21. Forest plot of comparisons of final successful recanalization (mTICI ≥2b) among prospective studies.

**
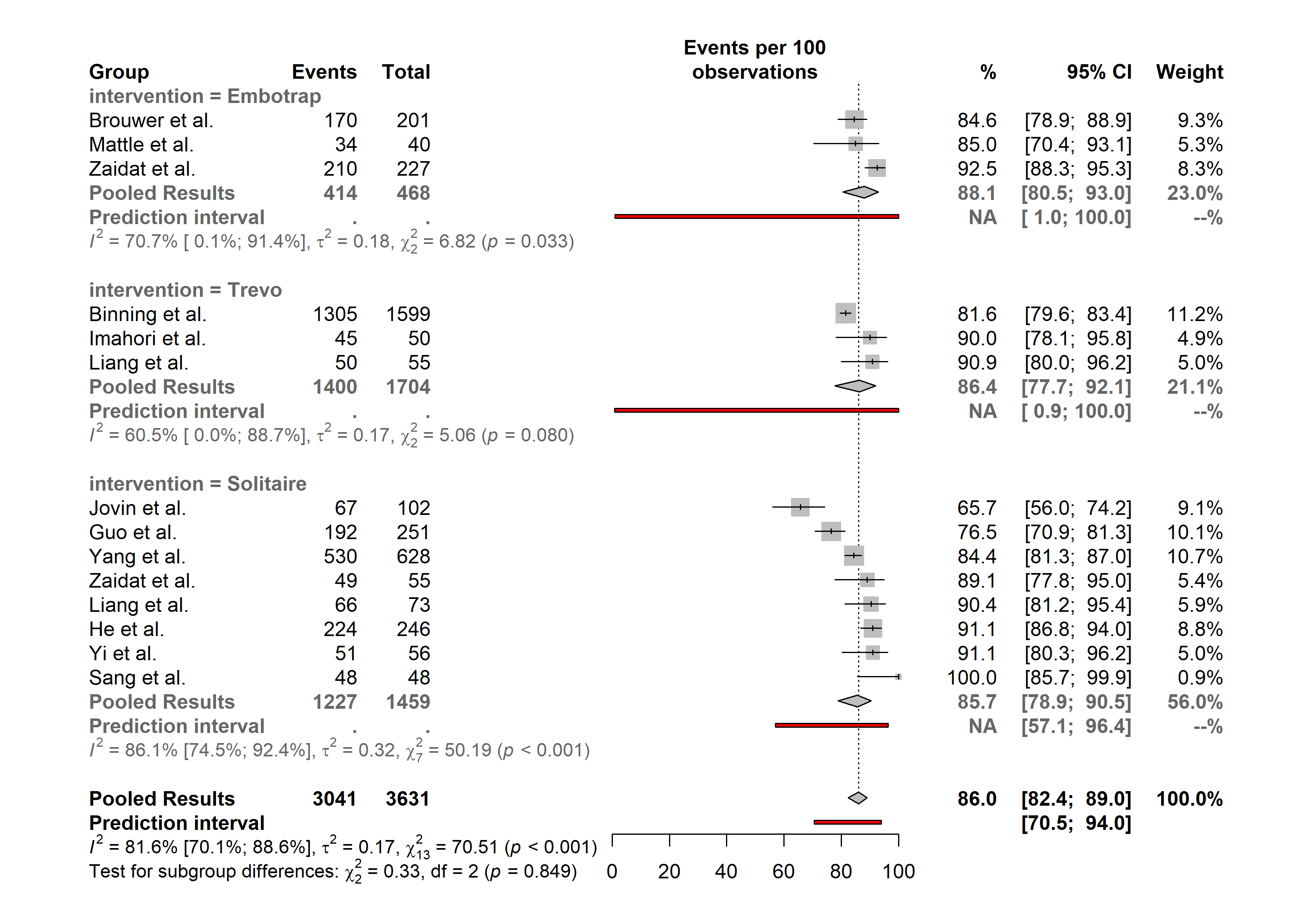
**

## Supplementary Figure 22. Forest plot of comparisons of ENT/distal emboli among prospective studies.

**
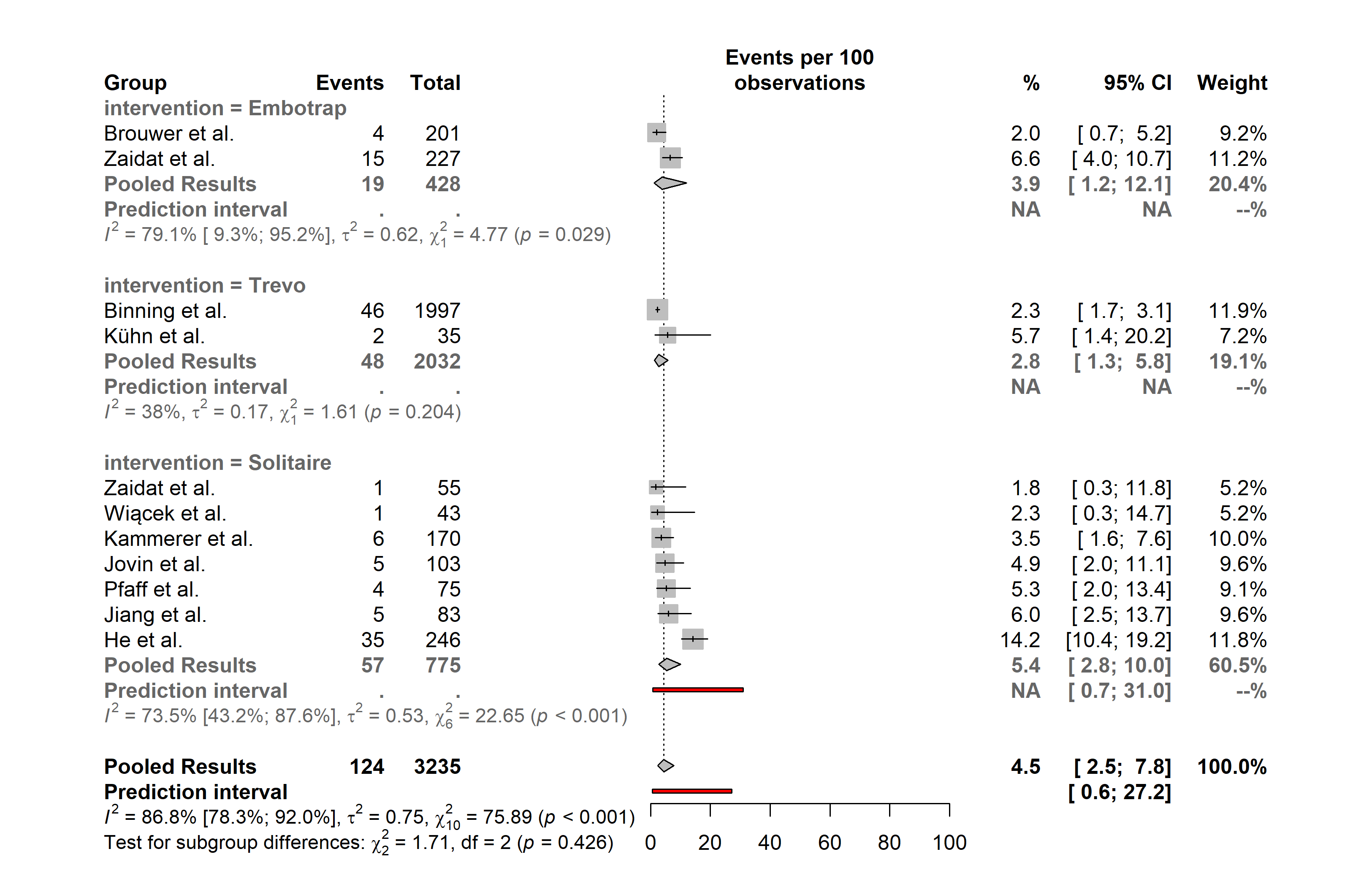
**

## Supplementary Figure 23. Forest plot of comparisons of sICH among prospective studies.


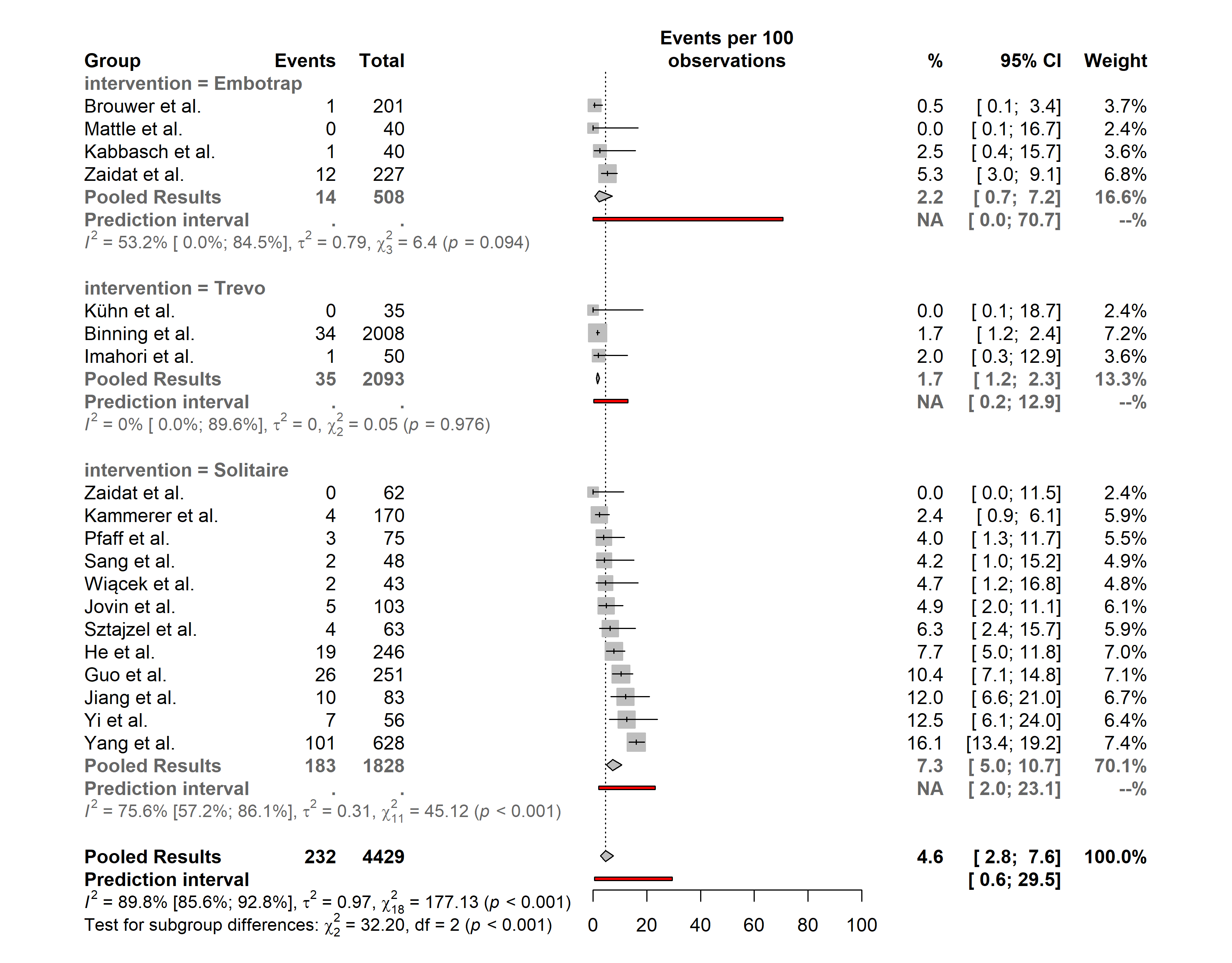


## Supplementary Figure 24. Forest plot of comparisons of mRS 0-2 at 90 days among prospective studies.


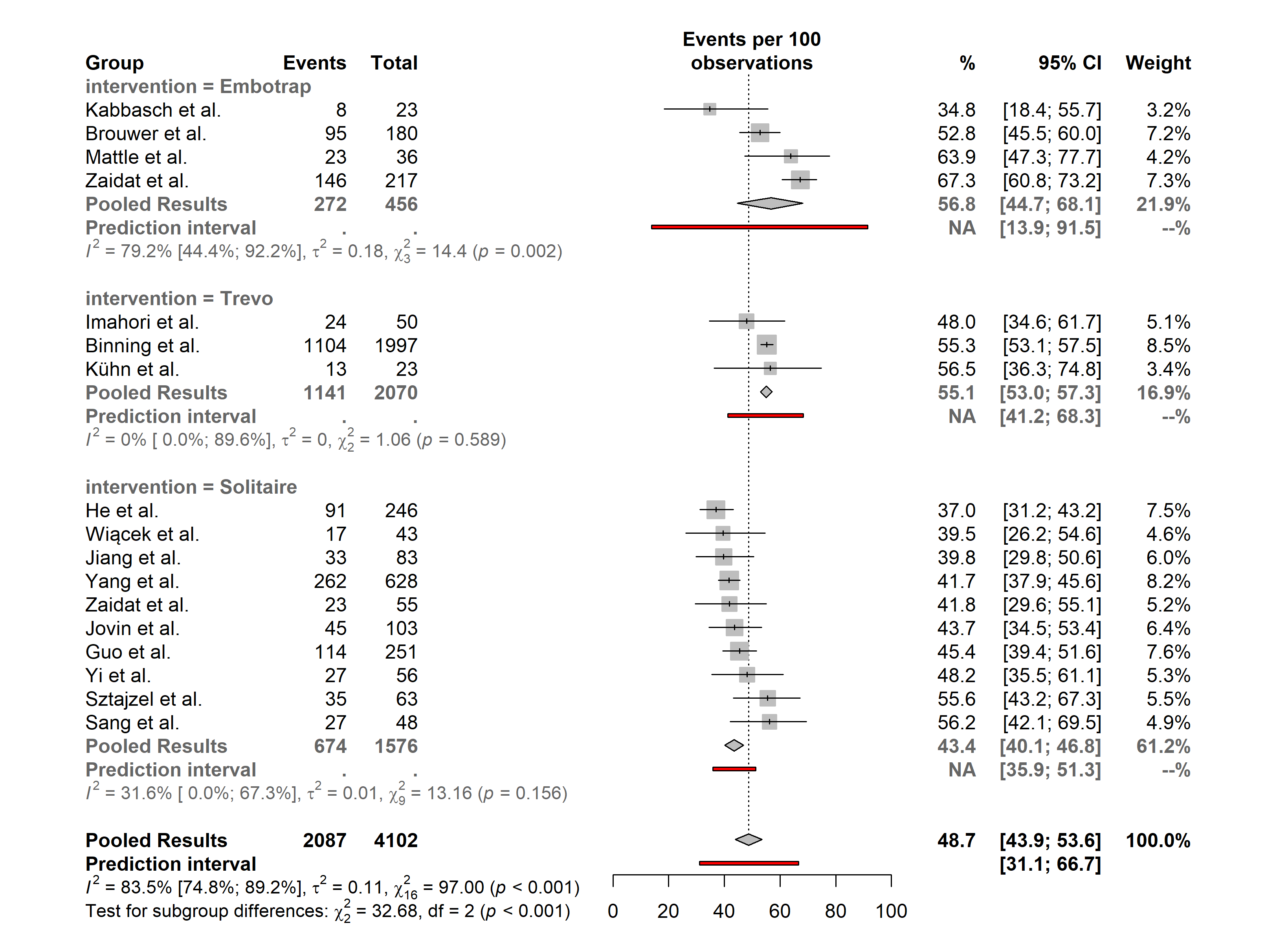


## Supplementary Figure 25. Forest plot of comparisons of mortality at 90 days among prospective studies.


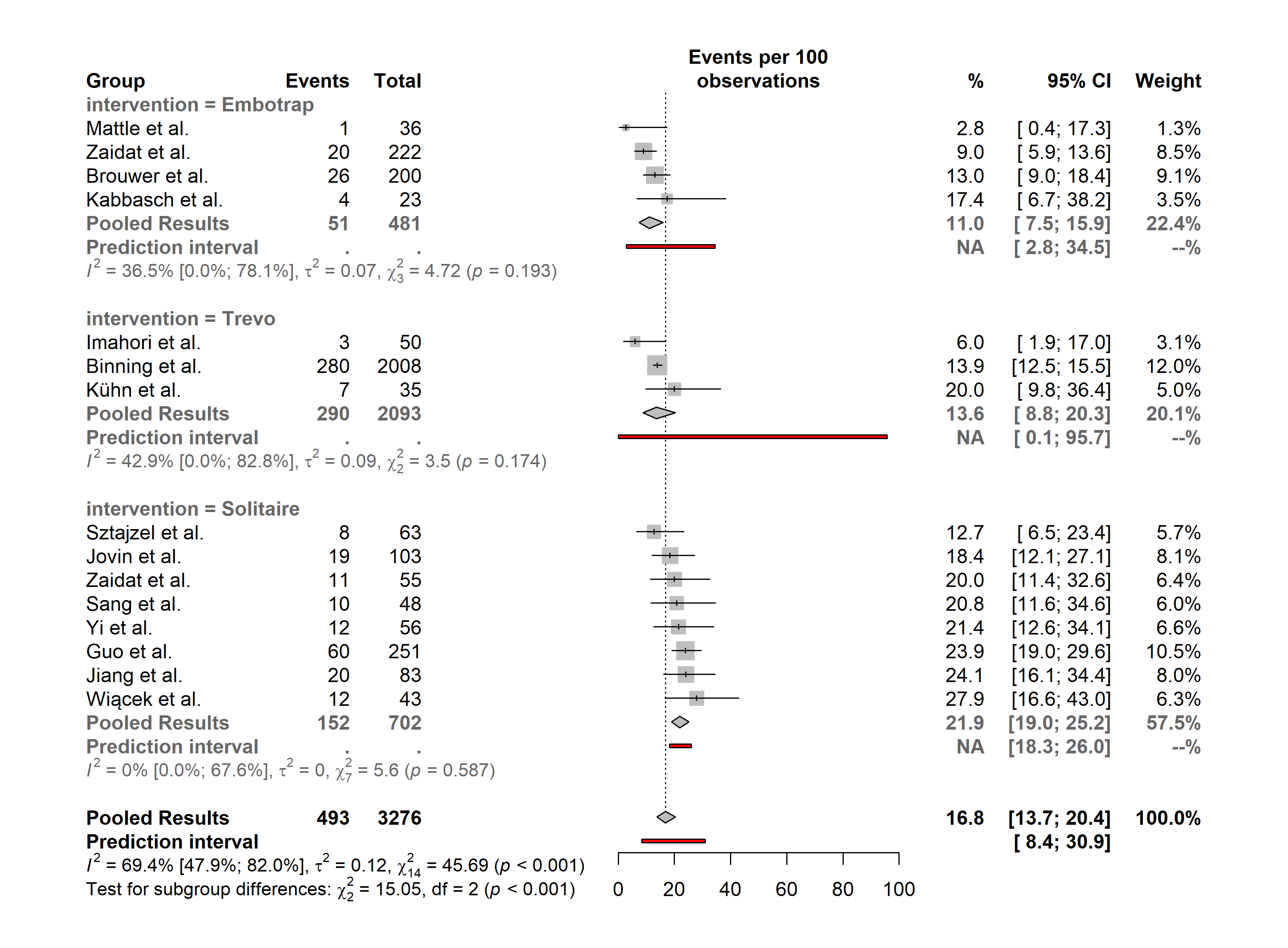

Supplement: Supplementary file 1 [file cer-12-230001-s1.docx]
