# Supplementary material for: MASTRO I: Meta-Analysis and Systematic Review of thrombectomy stent retriever outcomes: comparing functional, safety and recanalization outcomes between EmboTrap, Solitaire and Trevo in acute ischemic stroke
Source: J Comp Eff Res. 2023 Apr 11;12(5):e230001. doi: 10.57264/cer-2023-0001 (PMC10402757; doi:10.57264/cer-2023-0001)
Supplement: Supplementary file 2 [file cer-12-230001-s2.docx]

**Video abstract transcript**

**From:** Zaidat OO, Ikeme S, Sheth SA *et al*. MASTRO I: Meta-Analysis and Systematic Review of Thrombectomy Stent Retriever Outcomes: comparing functional, safety and recanalization outcomes between EmboTrap, Solitaire and Trevo in acute ischemic stroke. *J. Comp. Eff. Res.* 12(5), e230001 (2023).

**00:00:00**

Hi everyone, my name is Sam Zaidat, I’m a neurointerventionalist in Toledo, Ohio at Mercy Health System.

**00:00:07**

MASTRO I is a very important study for many good reasons [1]. In everyday practice, we need scientific evidence to compare devices in mechanical thrombectomy.

That’s not always feasible. It’s very costly to run a randomized trial in every question we have, and things evolve with our technology. So, it’s very critical to come up with technique where you can look at comparative studies without having necessarily to do a randomized trial for every question you have.

**00:00:34**

For example, in the case of stent retrievers here, we have many stent retrievers on the market, but it’s really no comparative studies comparing stent retriever A versus stent retriever B. So MASTRO I provide us those answer until other studies and scientific evidence come along. It’s a very quick and rigid and rigorous scientific way of looking at the clinical question in a procedural base to specialties, like neurointervention.

**00:01:01**

The MASTRO I study is a meta-analysis. It’s MASTRO I because it’s living meta-analysis that mean as more evidence becomes available it will be included in this meta-analysis.

**00:01:12**

It follows the PRISMA compliance and it follows the MOOSE compliance analysis for observational studies in meta-analysis to ensure there is no bias.

In this meta-analysis we excluded any patient with combination therapy beside the stent retriever. We focused on the Embotrap, Trevo and Solitaire stent retriever. We identified all the data that published on them.

**00:01:36**

More than 1500 articles were identified. From those, 51 studies were included that met the criteria – no combination therapy, so the patient has to have either Embotrap alone or Trevo alone or Solitaire. If they are using aspiration plus stent retriever, those patients were excluded. Also to avoid bias due to sample size, any study with less than 25 patients were excluded from the final analysis of the MASTRO I.

**00:02:04**

In MASTRO I, we focused on the typical outcome in ischemic stroke mechanical thrombectomy studies, which is the functional outcome as well as the recanalization or revascularization success.

So the result of the MASTRO I demonstrated at the functional outcome, which most of the clinicians really pay attention to the functional outcome and disability and reducing disability on stroke patient to undergo thrombectomy.

**00:02:27**

In this analysis what we found that when we compared the Embotrap to Trevo stent retriever in this population, we identified that Embotrap had statistically significant better clinical outcomes at 90 days when it compared to the Trevo stent retriever.

So the functional outcome and back to independent was higher – statistically higher – in the Embotrap group versus the Trevo stent retriever.

**00:02:53**

We did the same thing and we looked at the Embotrap versus Solitaire in this meta-analysis. We identified a similar pattern, that the functional outcome at 90 days was statistically higher in the Embotrap group versus the Solitaire group, with more patients who are independent at 90 days in the Embotrap mechanical thrombectomy ischemic stroke patient.

**00:03:16**

One way to explain the difference in this functional outcome is to look at the recanalization and reperfusion rate between those stent retrievers. When we evaluated the revascularization rate, whether it’s first pass reperfusion or final reperfusion rate, defined as the success TICI 2B or higher, we found that there is a higher number of revascularization rate in the Embotrap versus the Trevo group. That was not a statistically different but numerically higher in the Embotrap population.

**00:03:47**

The same trend was found in the Embotrap versus Solitaire, with a higher recanalization rate at the final evaluation of the recanalization or following the first pass. A similar trend was noted with the Solitaire, however that was also not statistically different.

**00:04:04**

When it came to the safety data in this meta-analysis, what we found that the rate of symptomatic haemorrhage was the lowest in the Embotrap group. And when it compared to the Solitaire that was statistically significant difference in the rate of symptomatic haemorrhage in the Embotrap versus the Solitaire. However, there was no difference between the Embotrap and the Trevo group.

**00:04:28**

Now, another safety outcome that we all look into and trying to identify if a stent retriever A or B or C affect the outcome in the angio suite is the embolization into new territory, or ENT outcome. When a clot goes to a different location than what we started with and usually that’s not a favourable angiographic outcome.

**00:04:48**

In that scenario we looked at the three groups, Embotrap versus Trevo and versus Solitaire and the rate of the ENT, and we found no difference between those three groups when it came to the ENT outcome.

Also, further out when we look at the mortality at 90 days and as a safety outcome in those three stent retrievers, we found that the mortality rate was lower in favour of the Embotrap over the Solitaire at 90 days. There was no difference between Embotrap and Trevo in mortality at 90 days.

**00:05:19**

To summarize the study result, in this scenario where we found that Embotrap performed better with a clinical outcome at 90 days and more patients returned to independence at 90 days versus Solitaire and Trevo, with less symptomatic haemorrhage than Solitaire and lower mortality rate. In that scenario I think you have to ask yourself: why did Embotrap perform better?

**00:05:42**

We already see one of the explanation in the study: that the recanalization rate was higher in number – although not statistically significant, but in number. There’s a higher first pass reperfusion rate and there is a higher recanalization rate which as we know that recanalization and revascularization – in particular the first pass through perfusion – translate to a functional outcome. Even though any percentage you may gain in a higher recanalization rate, at the first pass or at the final result will translate to a better functional outcome.

**00:06:15**

And the magnitude and the relationship we don’t understand fully how much you gain down the line, so that’s one simple explanation. Of course, the study has limitations and other things that could have contributed that Embotrap performed better at the clinical outcome.

**00:06:30**

One of the things that people may remember that in ARISE II [2], people were also surprised that our 90 days outcome was 67 percent – almost two out of three patients regain independence in ARISE II, which is a study with independent monitor, independent evaluator. This is a study where it confirms those findings into the same trajectory in the same trend.

Furthermore, when we looked at other data, for example from Mirza *et al*. have looked at the recanalization rate between Embotrap and Solitaire and they found similar trend – higher rate of recanalization in the Embotrap group [3].

**00:07:05**

Also a very interesting study from Grady group, Emory group that they looked at the combination: therapy aspiration plus stent retriever versus stent retriever alone [4]. This was another example why those studies are very critical, because it’s hard to do this in a randomized fashion and do another randomized trial and another randomized trial to compare combination therapy, aspiration plus stent retriever, versus stent retriever alone.

**00:07:31**

In this study they didn’t find any difference between a stent retriever or combination therapy. However, when they looked at the subgroup analysis, if you combined the aspiration catheters with an Embotrap, take that subgroup alone and the combination therapy, then the combination therapy outperformed the stent retriever alone. So, this is also another signal to indicate the direction of the high recanalization with the Embotrap.

**00:07:59**

Now you have to ask yourself why Embotrap in its design and physical attributes may be performing better in recanalization rate.

And this result also beside the data that showed the same trajectory of result may be related to the design of the Embotrap and the foundation of where this design came from.

**00:08:18**

The group at CERENOVUS have done a great job in establishing a clot research group called NTI. And this really went into the clot extensively and designed clot with a friable, a harder clot and medium hardness clot, and the design and the iteration of the Embotrap was based on clot research which may have led to a higher rate of recanalization.

**00:08:43**

Finally, you know, every study has its own limitations and in the MASTRO I we realize there is limitations, for example: including observational studies. But we all know in thrombectomy and interventional stroke studies, many studies are observational, and they have good values and they have moved the field forward. So, we included some of those studies but applied rigorous inclusion and exclusion criteria.

**00:09:08**

The second thing one may argue is the heterogeneity in our meta-analysis. We applied the outlier and influencer analysis to try to reduce the heterogeneity. For example, when we looked at the ENT outcome, three studies were identified that suffer from this limitation being outlier or influencer. When we eliminated those three studies our heterogeneity improved and reduced by 30 percent versus not removing them, so we did try to improve on the heterogeneity of our meta-analysis by applying this methodology.

**00:09:41**

Finally, people may argue that BGC or the use of IV-TPA are more common in the Embotrap and may have influenced the outcome that we saw as a result of this meta-analysis in Embotrap group. However, we could not eliminate those studies because, for example, we could not really do also a subgroup analysis because those results were not reported separately.

For example, the BGC we didn't find the haemorrhage rate or the mortality or the functional outcome separate or recanalization, so we couldn't do a sensitivity analysis by performing a subgroup study on those patients with IV-TPA for example or BGC.

**00:10:19**

So overall we believe we did everything possible in methodology to make sure our analysis is sound, up to the level of the data that we included in our meta-analysis.

So, what MASTRO I brings to the table - what does it really tell us, what's the story, you know, that we want to get out of this MASTRO I meta-analysis?

**00:10:39**

It's the first step toward creating an evidence-based decision-making when there is no randomized clinical trial. I'm sure in the next few years we may see more randomized head-to-head research comparing device A versus device B but for the current time we’re in, I think applying methodology like MASTRO I to help us on our daily practice is key for all of us.

**00:11:02**

In this case it really optimized the decision making, the clinical decision making, what to go for in the middle of the night when you are encountering a patient. So, the take home message is we need more of those studies to put all the data together to come up with some sort of a decision-making tool for us to help us making the decision in the middle of the night.

**00:11:22**

Again, we are awaiting more randomized trial, more definitive evidence but it's not always available and it takes time to establish such evidence.

Meanwhile we can use those method like MASTRO I to come up with decision-making tools. Also what we really think unique about MASTRO I, it's a living statement, meaning that in 12 to 24 months we will update the data, we'll update the results based on availability of literature and research out there and come up with – it could be the same decision, it could be different decision making, again this is a tool, it's a dynamic tool that we will use in the future for our patient and our clinical decision making.

**References**

1. Zaidat OO, Ikeme S, Sheth SA *et al*. MASTRO I: Meta-Analysis and Systematic Review of Thrombectomy Stent Retriever Outcomes: comparing functional, safety and recanalization outcomes between EmboTrap, Solitaire and Trevo in acute ischemic stroke. J. Comp. Eff. Res. 12(5), e230001 (2023).
2. Zaidat OO, Bozorgchami H, Ribo M *et al*. Primary results of the multicenter ARISE II study (Analysis of Revascularization in Ischemic Stroke With EmboTrap). *Stroke* 49(5), 1107–1115 (2018).
3. Mirza M, McCarthy R, Gilvarry M. EP64 Systematic review and analysis of pre-clinical side-by-side comparisons of EmboTrap versus Solitaire performance. *J. Neurointerv. Surg.* 13, A26 (2021).
4. Mohammaden MH, Haussen DC, Pisani L *et al*. Stent-retriever alone vs. aspiration and stent-retriever combination in large vessel occlusion stroke: a matched analysis*.* *Int. J. Stroke* 17(4), 465–473 (2022).
